# Supplementary material for: Evidence from the first Shared Medical Appointments (SMAs) randomised controlled trial in India: SMAs increase the satisfaction, knowledge, and medication compliance of patients with glaucoma
Source: PLOS Glob Public Health. 2023 Jul 20;3(7):e0001648. doi: 10.1371/journal.pgph.0001648 (PMC10358908; doi:10.1371/journal.pgph.0001648)
Supplement: S1 Appendix — (DOCX) [file pgph.0001648.s002.docx]

**S1 Appendix**

This appendix has been provided by the authors to give readers additional information about their work.

Supplement to: Evidence from the First Shared Medical Appointments (SMAs) Randomised Controlled Trial in India: SMAs Increase the Satisfaction, Knowledge, and Medication Compliance of Patients with Glaucoma

**S1 Appendix**

Supplement to “Evidence from the First Shared Medical Appointments (SMAs) Randomised Controlled Trial in India: SMAs Increase the Satisfaction, Knowledge, and Medication Compliance of Patients with Glaucoma”

TABLE OF CONTENTS:

**List of Investigators**

Trial Group Investigators**……………………………………………………………………………………………………….………....3**

**Supplementary Methods**

Review Methods**………………….……………………………………………………………………………….…………………….…3**

Intervention and Enrolment Details**………………………………………………………………………………….………………….…3**

Post Enrolment Appointments Details**………………………………………………………………………………………………….…4**

**TRIAL GROUP INVESTIGATORS** We are grateful to study co-investigators, implementation, and management teams at the Aravind Eye Hospital, Pondicherry, India.

**REVIEW METHODS**

We undertook five different searches using Medline and the Cochrane libraries to identify articles as of November 25, 2022. First, we conducted a search for shared medical appointment (SMA) related terms and we found 641 research papers, using search terms listed in (1) below. Then, we conducted a search for SMA related terms occurring in research papers where the research site was in Asia, Africa or Latin America. The search terms are listed in (2), (3) and (4) below. We chose all of the low to moderate income countries, using both their name and adjective form. We found 41 research papers in Asia, 12 research papers in Africa and no papers in Latin America. Our final search was on SMA and eye diseases related terms, and there were 6 papers in total. The search terms are listed in (5) below.

1. “group or shared” and “visit or visits or appointment or appointments”, “shared medical appointment or shared medical appointments or group medical visits”.
2. (“group or shared” and “visit or visits or appointment or appointments”, “shared medical appointment or shared medical appointments or group medical visits”) and **(China**or India or**Indonesia**or**Pakistan**or**Bangladesh**or**Japan**or**Philippines**or **Vietnam**or**Turkey**or**Iran**or**Thailand**or **Myanmar**or **South Korea**or **Iraq**or **Afghanistan**or **Saudi Arabia**or **Uzbekistan**or **Malaysia**or **Yemen**or **Nepal**or **North Korea**or **Sri Lanka**or **Kazakhstan**or **Syria**or **Cambodia**or **Jordan**or **Azerbaijan**or **United Arab Emirates**or **Tajikistan**or **Israel**or **Laos**or **Lebanon**or **Kyrgyzstan**or **Turkmenistan**or **Singapore**or **Oman**or **Palestine**or **Kuwait**or **Georgia**or **Mongolia**or **Armenia**or **Qatar**or **Bahrain**or **Timor-Leste**or **Cyprus**or **Bhutan**or **Maldives**or **Brunei)**
3. (“group or shared” and “visit or visits or appointment or appointments”, “shared medical appointment or shared medical appointments or group medical visits”) and (Algeria or Angola or Benin or Botswana or Burkina Faso or Burundi or Cameroon or Cabo Verde or Central African Republic or Chad or Comoros or Congo or Côte d'Ivoire or Democratic Republic of the Congo or Equatorial Guinea or Eritrea or Ethiopia or Gabon or Gambia or Ghana or Guinea or Guinea-Bissau or Kenya or Lesotho or Liberia or Madagascar or Malawi or Mali or Mauritania or Mauritius or Mozambique or Namibia or Niger or Nigeria or Rwanda or Sao Tome and Principe or Senegal or Seychelles or Sierra Leone or South Africa or South Sudan or Swaziland or Togo or Uganda or United Republic of Tanzania or Zambia or Zimbabwe)
4. (“group or shared” and “visit or visits or appointment or appointments”, “shared medical appointment or shared medical appointments or group medical visits”) and (Brazil or Mexico or Colombia or Argentina or Peru or Venezuela or Chile or Guatemala or Ecuador or Bolivia or Haiti or Cuba or Dominican Republic or Honduras or Paraguay or Nicaragua or El Salvador or Costa Rica or Panama or Uruguay or Jamaica or Trinidad and Tobago or Guyana or Suriname or Belize or Bahamas or Barbados or Saint Lucia or Grenada or St. Vincent & Grenadines or Antigua and Barbuda or Dominica or Saint Kitts & Nevis)
5. (“group or shared” and “visit or visits or appointment or appointments”, “shared medical appointment or shared medical appointments or group medical visits”) and (“eye diseases” or “ophthalmology” or “group medical visits”)

**INTERVENTION AND ENROLMENT DETAILS** During each week of the enrolment period, the two doctors involved in the study provided the study coordinators with their availability (days and times free) for the study in the following week. The study coordinators then scheduled appointment time slots for groups of five patients and invited patients who met the inclusion criteria in groups of five, via individual phone calls, making a reminder call three days prior and one day prior to the appointment date.

Patients first registered at the hospital front desk and then arrived at the Glaucoma Clinic. They then underwent all or some of the following steps, depending on their disease stage and requirements: history, vision check, and eye pressure check, preliminary doctor visit and investigations including Field Analysis and Optical Coherence Tomography. These steps were conducted by a mid-level ophthalmic professional or a junior doctor. During the waiting time between these tests, our study coordinators informed patients one by one about the consent material (included in the Study Protocol). Those patients who agreed to the terms were enrolled in the study. Thus, patients had another chance to decline joining the trial if they had concerns about privacy or other issues. During the enrolment period, vacancies due to patients declining to join at this point or due to no shows were filled by recruiting patients who were visiting the Glaucoma Clinic and who satisfied the inclusion criteria, to ensure groups of five, after obtaining written informed consent. Data about patient attrition during the trial is detailed in Figure 1 of the manuscript.

**POST-ENROLMENT APPOINTMENTS DETAILS** Patients in groups assigned to the treatment arm attended shared medical appointments all through the trial, while those in groups assigned to the control arm attended one-on-one appointments throughout the trial. At the end of each visit, patients were scheduled for their next visit; there were four visits in total, spaced about four months apart. Visits occurred Tuesday through Friday.

In the period prior to each appointment, the study doctors informed the study coordinators of any unforeseen schedule changes and patients were contacted via phone to reschedule their appointment if needed. One week and again 1 day prior to each appointment, the study coordinators called all patients to remind them of the upcoming appointment. Patients could drop out of the trial at any time.
