# Supplementary material for: Evidence from the first Shared Medical Appointments (SMAs) randomised controlled trial in India: SMAs increase the satisfaction, knowledge, and medication compliance of patients with glaucoma
Source: PLOS Glob Public Health. 2023 Jul 20;3(7):e0001648. doi: 10.1371/journal.pgph.0001648 (PMC10358908; doi:10.1371/journal.pgph.0001648)
Supplement: S2 Appendix — (PDF) [file pgph.0001648.s003.pdf]

S2 Appendix

This appendix has been provided by the authors to give readers additional information about their work.

Supplement to: Evidence from the First Shared Medical Appointments (SMAs) Randomised Controlled Trial in India: SMAs Increase the Satisfaction, Knowledge, and Medication Compliance of Patients with Glaucoma

This supplement contains the following items:

1. Original protocol, ethics approval letters, amendments, case report form, patient information sheet and informed consent form in English and Tamil.

Contents

Section 1 - Protocol……………………………………………......1

Section 2 - Ethics Approval Letters………………………..……....16

Section 3 - Amendments……………………………………….....26

Section 4 - Case Report Form…………………………………....39

Section 5 - Patient Information Sheet………………………………82

Section 6 - Informed Consent Form (English)……………………...87

Section 7 - Informed Consent Form (Tamil)………………………90

Section 1 – Protocol

Title of the Project
Shared Medical Appointments Randomized Trial (SMART)

Project Duration

Duration: June 1, 2016 through December 31, 2017.

Enrollment period of 6 months: June 1, 2016 to December 31, 2016

Follow-up period of 12 months: Ending December 31, 2017

(Data from 4 pre-trial and 4 post-trial follow up will be collected from the patient's case records)

Principal Investigator (with designation)
Dr S. Kavitha, Senior Glaucoma Specialist, Aravind Eye Hospital, Pondicherry

Co-investigators (with designation)
Ryan Buell, Assistant Professor of Business Administration, Harvard Business School

Kamalini Ramdas, Professor of Management Science & Operations, Deloitte Chair in Innovation & Entrepreneurship and Academic Director, Deloitte Institute of Innovation & Entrepreneurship, London Business School

Dr R. Venkatesh, Chief Medical Officer, Aravind Eye Hospital, Pondicherry

Collaborator with designation and affiliation
Nazli Sonmez, PhD student, London Business School

Funding agency
The costs for this research project will be funded through research budgets at London Business School and Harvard Business School, in addition to possible outside funding.

Budget

Funding will be used for the following activities:

New Study Administrator Position: The researchers will fund a new study administrator position at Aravind Eye Hospital to administer the study for the entire duration of the project. The salary of this administrator will total approximately £200 per month or less. This administrator will be hired by the Aravind Pondicherry Glaucoma Clinic.

Video recording: We intend to video record the final examination stage of the appointments for patients in the treatment and the control group, using wall mounted video cameras. Cost is estimated as a maximum of £500 per camera and equipment, maximum £2,000 total for four cameras.

Video transcription: As described below, we also intend to create de-identified transcripts of the videos to facilitate in-depth analysis of drivers of patient behaviour. Cost is estimated as a maximum of £14,000.

Background

The most fundamental vehicle of healthcare delivery – the doctor-patient interaction – has remained unchanged for millennia. All over the world, the most common and often the only way to meet a physician for non-urgent care is through a one-on-one appointment. An alternative to the one-on-one doctor visit has been practiced successfully by a few providers in the US for over 15 years. In a *shared medical appointment* (SMA) a group of 5-8 patients with similar chronic conditions – such as diabetes – meet with a doctor simultaneously. Each patient in turn receives full one-on-one attention including diagnosis and prescription, while the others listen.

Patients in an SMA have similar chronic conditions so the doctor need not repeat common advice – which is a large part of most one-on-one appointments. As SMAs substitute for one-on-one appointments, doctors can use the time saved in SMAs to provide better individual medical attention and forge patient relationships. Any remaining time contributes to productivity gains. Although doctors engaged in SMAs often spend less time with each patient on average due to the batch processing, patients in an SMA spend more total time with the doctor and are exposed to more information. In an SMA, patients with similar conditions learn from one another and from the doctor's responses to other patients' questions. A shared appointment in effect brings a support group into the doctor's office. Patients' realization that they are not alone in their difficulties can reduce anxiety. A desire to conform or excel in a group may spur them to adhere to the doctor's instructions. Thus SMAs for chronic diseases have the potential to serve as a catalyst for patient behavior change.

SMAs have been implemented for diabetes, cardiac preventive care, asthma and other chronic diseases. Clinics that offer shared appointments have noted improved patient outcomes, greater rates of compliance and adherence, and increases in capacity (Bronson and Maxwell 2004). SMAs often prove to be successful where adopted, but their benefits have not been rigorously and scientifically documented. In their recent review paper, Edelman et al. (2012) note that the evidence base on SMAs is very scant. Furthermore, the existing research has focused mainly on medical outcomes rather than behaviors that may impact outcomes. Even more importantly, no one has examined the underlying mechanisms by which SMAs may induce behavior change.

Major Objectives

The primary objectives of this research project are to examine how SMAs for the final examination stage of a glaucoma appointment affect the level of patient knowledge and follow-up rates in glaucoma care provision. Understanding the nature of glaucoma, the implications of following the course of treatment, and persisting with the treatment regime, are important precursors to improved medical outcomes.

In support of these primary objectives, to understand what is driving any observed change in knowledge levels and patient follow-up rates, we will measure the extent of peer-to-peer learning and exposure to information. This will improve our understanding of how the design of an SMA – such as patient mix in terms of disease progression levels, education levels, and other measures of patient diversity – impacts knowledge levels, follow-up rates, and in turn, outcomes.

We also intend to examine changes in underlying patient behaviors as well as patients' satisfaction with care provided, that may link increased knowledge levels and follow-up rates engendered by SMAs to changes in medical outcomes. Behavioral outcomes include propensity to adhere to prescribed medicines and courses of treatment. To assess the links between these behaviors and medical outcomes, we will explore how SMAs and these intermediate behaviors impact vision and intra-ocular pressure over the course of the study.

Finally, we intend to examine the efficiency of SMAs, with respect to operational and financial outcomes. Operational outcomes include the average amount of time the doctor spends per patient, and the amount of waiting time. Financial outcomes

include estimates of the potential cost savings and/or revenue gains that can accrue from being able to treat more patients in the same time.

Methodology

We propose conducting a randomized control trial (RCT) at Aravind Eye Hospital's Glaucoma Clinic, in Pondicherry, India, from June 1, 2016 through December 31, 2017. We will analyse the data collected using quantitative and qualitative research methods. We propose an enrollment period of 6 months (June 1, 2016 to December 31, 2016) and a follow-up period of 12 months for each enrolled patient, to capture changes in patient prognosis over three follow-up visits, each spaced four months apart. We will collect study data continuously over this period, so that preliminary outcomes can be examined prior to the official end of the study.

Based on prior research (Edelman, et al. 2012) and a power analysis we conducted with pre-pilot study data comparing the knowledge levels and follow-up rates among patients who experienced SMAs and traditional appointments, we propose an initial target sample size of 1,000 (with 500 patients in each condition). We calculate that such a sample size will ensure a study with 90% power (β=0.01) and the ability to calculate differences among sample means with 99% confidence (α=0.01). We propose to conduct additional power analyses based on data collected from from June 1, 2016 to June 30, 2016, to further calibrate the sample size (Ahn, et al. 2014; Altman 1980; Chow, et al. 2007; Schultz and Grimes 2005).

Flow of qualified patients during the enrolment period: During SMA clinic days (Tuesday – Friday during the period of enrolment), when qualified patients arrive who have not yet been enrolled in the study, they will follow a typical patient flow:

1. Patient is registered
2. Patient’s case record is retrieved
3. Patient is transported to the glaucoma clinic
4. The patient will undergo all or a subset of the processes below:
    a. Vision check/ Refraction
    b. Take history and check eye pressure
    c. Preliminary doctor visit
    d. Additional testing - HFA
5. During the waiting time between steps listed in #4 above, the study coordinator will review the informed consent material with the pre-qualified patient, and if the patient agrees to the terms, he or she will be enrolled in the study. De-identified data will be collected on which patients choose not to participate.
6. The patient undergoes dilatation
7. Groups of five enrolled patients will be formed in the order of their arrival at the clinic. Groups will randomly be assigned to the treatment (SMA) or control (individual appointment) conditions.
8. Patient will undergo OCT if advised in a previous visit
9. Patient will experience SMA or individual appointment
10. Patient will receive follow-up survey
11. Patient will undergo any additional typical process steps, which may include meeting the study coordinator for counseling and/or scheduling a follow-up appointment on a day when SMAs are conducted (Tuesday – Friday, at specified times during the morning).
12. Any cross reference to other departments will be done once the patient completes the above steps

Flow of enrolled patients during follow-up appointments: Several days in advance of an enrolled patient's follow-up appointment, the study coordinator will contact the patient by telephone to remind the patient about their appointment and confirm their availability. If the patient will not be available to attend their appointment at their scheduled time, the appointment will be rescheduled on a day when SMAs are conducted (Tuesday – Friday, at specified times during the morning).

During SMA clinic days (Tuesday – Friday, at specified times during the morning, throughout the period of the study), when enrolled patients arrive for follow-up appointments, they will follow a typical patient flow:

1. to 4. Same as above
5. The patient undergoes dilatation
6. The Patient will undergo OCT if advised in a previous visit.
7. Patients who have been assigned to the treatment condition will be routed to experience an SMA, and patients who have been assigned to the control condition will be routed to experience an individual appointment. Depending on the number of patients in the treatment condition who are scheduled to come to the glaucoma clinic for a follow-up appointment on a given day, the appropriate number of SMA sessions will be chosen, such that patients will be assigned to an SMA with approximately five people. However, we recognize that the number of patients per SMA will vary based on patient arrivals.
8. Patient will receive follow-up survey as detailed below

9. Patient will undergo any additional typical process steps, which may include meeting the study coordinator for counseling and/or scheduling a follow-up appointment.

Treatment Procedure: Patients in the treatment condition will experience an SMA – an appointment shared with other patients. We will target five patients per SMA during this study, though the number will vary based on patient arrivals.

Control Procedure: Patients in the control condition will experience an individual appointment – a one-on-one consultation with the physician.

Dropouts: Any patient who voluntarily drops out of the study will be dis-enrolled from the study and returned to the regular patient stream. The fact that the patient dropped out of the study will be recorded in the study data.

Measures and Sample Collection

Measures

We propose collecting a variety of measures to quantify the efficiency and efficacy of SMAs relative to individual appointments, and to understand the mechanisms through which SMAs affect outcomes.

Efficacy Measures will include medical outcomes (e.g. vision and intra ocular pressure) and behavioral outcomes (e.g. propensity to follow up and propensity to adhere to medications.)

Efficiency Measures will include actual duration of each appointment, number of patients in each appointment, and time stamp data on patient waiting time at each stage in the glaucoma clinic process.

To the extent that they are available, these efficacy measures will be collected for each patient during the four sessions included during the study period, as well as the four sessions preceding and the four sessions following the study period. Collecting data prior to the study will facilitate a pre-period trends analysis, to ascertain whether participants in the treatment and control groups were on similar trajectories in terms of their medical prognosis, engagement in their treatment, and propensity to follow-up, prior to the introduction of shared medical appointments for treated patients. Collecting data from after the study will facilitate understanding whether the introduction of shared medical appointments had lasting effects beyond the period of the study. To the extent that shared medical appointments may reframe the patients' relationship with glaucoma and with their doctor, their introduction may have a lasting effect of behavior that is measurable even after the treatment subsides.

Mechanisms measures will include assessment of patients' knowledge about glaucoma (e.g. what it is, what causes it, how to treat it, etc.), patients' perceptions about quality of care provided during an appointment, and the extent of factors such as peer-to-peer learning and exposure to new information.

Sample Collection

The study coordinator will be responsible for sample data collection. The study coordinator will create a unique identifier for each appointment (so that we can identify which patient was in which appointment), a participant ID number in the study which can be linked to Medical Record Number, and de-identified coding of which doctor conducted each appointment.

Surveys: All patients in the treatment and control groups will respond to a survey at the end of the final examination stage in each of their appointments during the trial. These surveys will contain questions to assess patient knowledge and patient perceptions about quality of care. In the final follow up appointment of the trial, patients will in addition be administered an exit survey in which they will be asked questions regarding factors that influence their propensity to follow up and their propensity to adhere to medications. Treatment group patients will in addition be asked about their interest in continuing SMAs, their propensity to recommend SMAs to others, and the reasons they may have preferred / not preferred SMAs to one-on-one appointments that they have experienced in the past.

Video recording: We propose that the individual appointments and SMAs of patients who are enrolled in the study will be video recorded, so that the sessions can be transcribed and analyzed at a later time. The video recordings will be kept securely, maintaining patient confidentiality. The recording process will be described fully in the informed consent materials. We propose taking the following steps to protect patient confidentiality with regard to video recordings:

1. Video recordings will be transcribed by a professional transcription service within India, and the transcribers will have access to no information about the patients apart from the contents of the videos.

2. Transcriptions will include the full spoken text of the session, but patient names will be removed. Transcribers will be asked to codify specified behaviors, such as: a) The degree to which patients in the session appeared attentive. b) The number of questions patients in the session asked. c) The

duration of the session. This information will help the researchers identify mechanisms of impact, such as peer-to-peer learning, and the amount of information shared in each session.

3. Transcripts will be indexed by an appointment identifier, which will link to the participant ID in a database maintained by the study coordinator at Aravind Eye Hospital. As such, the researchers will not have access to the videos, nor any identifiable information in the transcripts that will be used for the analysis.

4. Following transcription, the videotapes will be retained securely for a period of six years, to allow sufficient time for the peer-review publication process and to facilitate the transcription of additional moderators as necessary, following the procedure outlined above. After six years, the videotapes will be destroyed, to protect patient confidentiality. However, the de-identified codified behaviors, as well as the de-identified text transcriptions will be made available to the researchers for textual analysis.

Data from charts: In addition, during each appointment, the study coordinator will collect information on medical outcomes from the patients' charts, and will ensure measurement of operational outcomes such as time taken for each appointment and patient waiting times, using time stamps. Combined with hospital data on staff costs and patient revenues this information can be used to examine impact on financial outcomes.

Demographics and patient history: The study coordinator will also collect data on all relevant control variables. Demographics will include gender, age, literacy

level, income proxies, rural vs. urban, etc. History will include patient comorbidities, years of glaucoma, and family history.

Inclusion/Exclusion Criteria: Pre-qualification and Inclusion Criteria: During the enrolment period of the study, the study administrator will contact patients in advance of their appointments to confirm the date and time of their appointments, and to pre-qualify patients to participate in the study. And also patients who have come on a non-appointment basis, if they satisfy the inclusion criteria will also be recruited if they are willing to participate. De-identified data will be collected on which patients choose not to participate. Patients who are willing to participate in a Shared Medical Appointment (SMA), and who meet the inclusion criteria specified below, will be considered pre-qualified for the study. The inclusion criteria are:

1. The patient must be a primary glaucoma patient,
2. The patient must not have had more than one surgery in one eye in the past,
3. The patient must not have undergone a tube shunt surgery,
4. The patient must not be monocular,
5. The patient should not require surgical intervention in the near future,
6. The patient must not wish to interact with a specific doctor,
7. It is believed that the patient will interact effectively in a group setting,
8. The patient should not be a part of any other existing trial, and;
9. The patient should not have any vision threatening condition other than glaucoma.

Exclusion Criteria:

1 .Any primary glaucoma patient who does not meet the inclusion criteria

2. Any glaucoma other than primary glaucoma

Risk & Benefits

Patient Risks and Ethical Approval

We plan to obtain data in a de-identified form and believe that the study does not pose any direct or indirect risk to patients' health. We do not plan to publish any data on individual patients. We will apply for IRB approval through the Aravind, London Business School and Harvard Business School processes. London Business School and Harvard Business School have already granted ethical approval for this project (approval form attached).

Project Risks

We do not foresee any major risks to the completion of the project and obtaining insights as envisaged above. However, the success of the project depends heavily on the ability to obtain high quality data required to conduct the statistical analysis as highlighted earlier. This will be ensured through the study coordinator.

Benefits

The direct benefits of this project include a better understanding of how SMAs impact medical, behavioral, operational and financial outcomes. This study will also throw light on the mechanisms through which SMAs may impact outcomes – e.g. peer-to-peer learning and patients' access to greater information. This study will also lay the foundation for future research at Aravind that can examine the long term benefits of SMAs for glaucoma and other eye diseases.

Expected Outcome

As described above, we believe that this study will enable us to examine the extent to which SMAs impact medical outcomes for glaucoma (vision and intraocular pressure in particular). In addition, it will generate fundamental insights regarding patient behaviours, knowledge and satisfaction that drive the observed changes in medical outcomes. We expect that these insights will be published in top academic journals in operations management (such as Management Science) and in top medical journals.

References:

Ahn, C., Heo, M., & Zhang, S. (2014). Sample Size Calculations for Clustered and Longitudinal Outcomes in Clinical Research. CRC Press.

Altman, D. G. (1980). Statistics and ethics in medical research: III How large a sample?. Br Med J, 281(6251), 1336-1338.

Chow, S. C., Wang, H., & Shao, J. (2007). Sample size calculations in clinical research. CRC press

Edelman, D., McDuffie, J. R., Oddone, E., Gierisch, J. M., Nagi, A., & Williams, J. W. (2012). Shared medical appointments for chronic medical conditions: a systematic review.

Schulz, K. F., & Grimes, D. A. (2005). Sample size calculations in randomised trials: mandatory and mystical. The Lancet, 365(9467), 1348-1353.

Thacker, H.L., Maxwell, R., Saporito, J., & Bronson, D. (2005). Shared medical appointments: facilitating interdisciplinary care for midlife women. Journal of Women's Health, 14(9), 867-870.

Section 2 – Ethics Approval Letters

Cuddalore Main Road, Thavalakuppam, Pondicherry-605 007, India
Phone: 0413-2619100; Fax: 0413-2618848; www.aravind.org

CHIEF MEDICAL OFFICER: Dr. R. Venkatesh DO, DNB
MANAGER-GENERAL: P. Kanagaraj B.E., M.I.E
MANAGER-ADMINISTRATION: P. Poobalan B.COM

ARAVIND EYE CARE SYSTEM

CHAIRMAN & DIRECTOR - QUALITY
Dr. R.D. Ravindran MS, DO

CHAIRMAN - EMERITUS & DIRECTOR - RESEARCH
Dr. P. Namperumalsamy MS, FAMS

DIRECTORS - EMERITUS
Dr. G. Natchiar MS, DO
Dr. M. Srinivasan MS, DO

DIRECTOR - FINANCE
G. Srinivasan BE, MS

DIRECTOR - OPERATIONS
R.D. Thulasiraj MBA

DIRECTOR - HUMAN RESOURCES
Dr. S.R. Krishnadas DO, DNB

DIRECTOR - INFORMATION TECHNOLOGY
Dr. R. Kim DO, DNB

DIRECTOR - ACADEMICS
Dr. N.Venkatesh Prajna DNB, FRCO

DIRECTOR - PROJECTS
Dr. S. Aravind MS, MBA

Institutional Ethics Committee

Date 27.05.2016

To

Dr.S.Kavitha,
Medical Consultant,
Glaucoma Services,
Aravind Eye Hospital, Pondicherry.

Ref: The study No. **AEH/PDY/EC/OA/15/2016** entitled **"Shared Medical Appointments Randomized Trial (SMART)".**

Investigators

1. Dr S. Kavitha, Senior Glaucoma Specialist, Aravind Eye Hospital, Pondicherry
2. Ryan Buell, Assistant Professor of Business Administration, Harvard Business School
3. Kamalini Ramdas, Professor of Management Science & Operations, Deloitte Chair in Innovation &Entrepreneurship and Academic Director, Deloitte Institute of Innovation & Entrepreneurship, London Business School
4. Dr R. Venkatesh, Chief Medical Officer, Aravind Eye Hospital, Pondicherry
5. Nazli Sonmez, PhD student, London Business School

Dear Dr.S.Kavitha,

The Meeting of the Institutional Ethics Committee (IEC) was held on **04.05.2016** at Aravind Eye Hospital, in the Conference Hall with Dr.Tiroumourougane Serane as Chairperson.

GENERAL OPHTHALMOLOGY
Dr. S. Josephine Christy
Dr. Indeevar V. Mishra
Dr. Sahil Bhandari
Dr. Rajesh V.
Dr. Priya S.
Dr. Annamalai O.
Dr. Balamuruganandaraj D.

RETINA – VITREOUS
Dr. Pankaja Dhoble
Dr. Manavi D. Sindal
Dr. Sabyasachi Sengupta
Dr. Prabu Baskaran

CATARACT
Dr. Manas Nath
Dr. Prasanth Gireesh

CORNEA
Dr. K. Tiruvengada Krishnan
Dr. N. Shivananda
Dr. Seema Ramakrishnan
Dr. Smitha Krishnamurthy

PAEDIATRIC OPHTHALMOLOGY
Dr. K. Veena
Dr. Marie Fredrick Mouttappa
Dr. Shelke Vijaysai Apparao

GLAUCOMA
Dr. R. Venkatesh
Dr. S. Kavitha
Dr. Swati Upadhyaya
Dr. Debasish Dash
Dr. Pavan Kumar M.G.

UVEA-OPHTHALMOLOGY
Dr. S. Bala Murugan

ORBIT & OCULOPLASTY
Dr. R. Jayagayathri
Dr. Dayakar Yadalla

NEURO-OPHTHALMOLOGY
Dr. K. Nirmala Devy

ARAVIND EYE CARE SYSTEM

ARAVIND EYE CARE SYSTEM
CHAIRMAN
& DIRECTOR - QUALITY
Dr. R.D. Ravindran MS, DO
CHAIRMAN - EMERITUS
& DIRECTOR - RESEARCH
Dr. P. Namperumalsamy MS, FAMS
DIRECTORS - EMERITUS
Dr. G. Natchiar MS, DO
Dr. M. Srinivasan MS, DO
DIRECTOR - FINANCE
G. Srinivasan BE, MS
DIRECTOR - OPERATIONS
R.D. Thulasiraj MBA
DIRECTOR - HUMAN RESOURCES
Dr. S.R. Krishnadas DO, DNB
DIRECTOR - INFORMATION TECHNOLOGY
Dr. R. Kim DO, DNB
DIRECTOR - ACADEMICS
Dr. N.Venkatesh Prajna DNB, FRCO
DIRECTOR - PROJECTS
Dr. S. Aravind MS, MBA

09 members attended the meeting. The list of members who attended the meeting is as follows

Name of Members	Position in IEC	Designation	Gender
Dr.V.Tiroumourougane Serane	Chairperson	Consultant Neonatologist	M
Mr.S.Sankaralingam	Legal Expert	Advocate	M
Mr. Ram Sehgal	Lay Person	Journalist	M
Mr.V.C.Raam Sukaesh	Lay Person	C.E.O, Vinbros and Co	M
Mr. K. Senthil Kumar	Social Scientist	Director, SARVAM, Sri Aurobindo Society	M
Dr. Kartik J. Salwe	Basic Scientist (Pharmacology)	Associate Professor, Dept of Pharmacology	M
Dr. Sharbari Basu	Basic Scientist (Biochemistry)	HOD, Dept of Biochemistry	F
Dr.R.Jayagayathri	Clinician	Medical Consultant in Orbit & Oculoplasty	M
Dr.N. Shivananda	Clinician	Senior Medical Consultant in Cornea	M

ARAVIND EYE HOSPITALS
& POSTGRADUATE INSTITUTE OF OPHTHALMOLOGY

Run by Govel Trust
Affiliated to
Dr. MGR Medical University

World Health Organization
Collaborating Centre
For Prevention of Blindness

PONDICHERRY
Cuddalore Main Road, Thavalakuppam, Pondicherry-605 007, India
Phone: 0413-2619100; Fax: 0413-2618848; www.aravind.org

CHIEF MEDICAL OFFICER: Dr. R. Venkatesh DO, DNB
MANAGER-GENERAL: P. Kanagaraj B.E., M.I.E
MANAGER-ADMINISTRATION: P. Poobalan B.COM

ARAVIND EYE CARE SYSTEM

CHAIRMAN & DIRECTOR - QUALITY
Dr. R.D. Ravindran MS, DO

CHAIRMAN - EMERITUS & DIRECTOR - RESEARCH
Dr. P. Namperumalsamy MS, FAMS

DIRECTORS - EMERITUS
Dr. G. Natchiar MS, DO
Dr. M. Srinivasan MS, DO

DIRECTOR - FINANCE
G. Srinivasan BE, MS

DIRECTOR - OPERATIONS
R.D. Thulasiraj MBA

DIRECTOR - HUMAN RESOURCES
Dr. S.R. Krishnadas DO, DNB

DIRECTOR - INFORMATION TECHNOLOGY
Dr. R. Kim DO, DNB

DIRECTOR - ACADEMICS
Dr. N.Venkatesh Prajna DNB, FRCO

DIRECTOR - PROJECTS
Dr. S. Aravind MS, MBA

It is hereby confirmed that neither you nor any of the study team members have participated in the voting/decision making procedures of the committee.

The IEC has reviewed and approved the following documents submitted for the above – mentioned clinical study at the meeting.

1. Form A, Form B
2. Protocol
3. Informed Consent Documents

The IEC hereby approves the proposal entitled, **"Shared Medical Appointments Randomized Trial (SMART)"**.

It is understood that the study will be conducted under your direction, in a total of **1000** research participants, at **Dept. of Glaucoma**, Aravind Eye Hospital, Pondicherry as per the submitted protocol.

This approval is valid for the entire duration of the study.

No deviations from, or changes of the protocol and Informed Consent Document should be initiated without prior written approval by the IEC of an appropriate amendment. The IEC expects that the investigator should promptly report to the IEC any deviations from, or changes of, the protocol to eliminate immediate hazards to the research participants and about any new information that may affect adversely the safety of the research participants or the conduct of the trial.

For studies which will continue for more than a year, a continuing review report needs to be submitted (within 1 month of the due date i.e. 11 months from the date of approval) on or before

04.04.2017.

A copy of the final report should be submitted to the IEC for review.

The IEC functions in accordance with ICH GCP, Schedule Y, ICMR guidelines and other applicable regulatory requirements.

Sincerely yours,

Signature of Chairperson

Date of Approval of the study: 27.05.2016

GENERAL OPHTHALMOLOGY
Dr. S. Josephine Christy
Dr. Indeevar V. Mishra
Dr. Sahil Bhandari
Dr. Rajesh V.
Dr. Priya S.
Dr. Annamalai O.
Dr. Balamuruganandaraj D.

RETINA – VITREOUS
Dr. Pankaja Dhoble
Dr. Manavi D. Sindal
Dr. Sabyasachi Sengupta
Dr. Prabu Baskaran

CATARACT
Dr. Manas Nath
Dr. Prasanth Gireesh

CORNEA
Dr. K. Tiruvengada Krishnan
Dr. N. Shivananda
Dr. Seema Ramakrishnan
Dr. Smitha Krishnamurthy

PAEDIATRIC OPHTHALMOLOGY
Dr. K. Veena
Dr. Marie Fredrick Mouttappa
Dr. Shelke Vijaysai Apparao

GLAUCOMA
Dr. R. Venkatesh
Dr. S. Kavitha
Dr. Swati Upadhyaya
Dr. Debasish Dash
Dr. Pavan Kumar M.G.

UVEA-OPHTHALMOLOGY
Dr. S. Bala Murugan

ORBIT & OCULOPLASTY
Dr. R. Jayagayathri
Dr. Dayakar Yadalla

NEURO-OPHTHALMOLOGY
Dr. K. Nirmala Devy

ARAVIND EYE CARE SYSTEM

HARVARD
Human Research Protection Program

Harvard University-Area
Committee on the Use of Human Subjects
1414 Massachusetts Avenue, 2nd Floor
Cambridge, MA 02138
IRB Registration - IRB00000109
Federal Wide Assurance - FWA00004837

Notification of Initial Study Approval

March 16, 2016

Ryan Buell
rbuell@hbs.edu

**Protocol Title:**	Shared Medical Appointments Randomized Trial (SMART)
**Principal Investigator:**	Ryan Buell
**Protocol #:**	IRB16-0213
**Funding Source:**	None
**Review Date:**	3/16/2016
**STUDY Effective Date:**	3/16/2016
**Expiration Date:**	3/15/2017
**IRB Review Type:**	Expedited
**IRB Review Action:**	Approved

On 3/16/2016, the Institutional Review Board (IRB) of the Harvard University-Area approved this Initial Study. **Please note that the approval for this protocol will lapse on 3/15/2017.**

The documents that were finalized for this submission may be accessed through the IRB electronic submission management system at the following link: Shared Medical Appointments.

The IRB made the following determinations:
• Special Populations: None
• Waivers: None
• Risk Determination: No greater than minimal risk
• Research Information Security Level: The research is classified, using Harvard's Data Security Policy, as Level 3 Data.

Please contact me if you have any questions at 617-496-9952 or twegner@hbs.edu.

Sincerely,

Toni Wegner
Coordinator, Protection of Research Participants, HBS

Harvard University-Area
Committee on the Use of Human Subjects
1414 Massachusetts Avenue, 2nd Floor
Cambridge, MA 02138
IRB Registration - IRB00000109
Federal Wide Assurance - FWA00004837

Notification of Continuing Review Approval

February 15, 2017

Ryan Buell
rbuell@hbs.edu

**Protocol Title:**	Shared Medical Appointments Randomized Trial (SMART)
**Principal Investigator:**	Ryan Buell
**Protocol #:**	IRB16-0213
**Submission #:**	CR16-0213-01
**Funding Source:**	Check listing
**Review Date:**	2/15/2017
**CR Effective Date:**	3/16/2017
**Expiration Date:**	3/15/2018
**IRB Review Type:**	Expedited
**IRB Review Action:**	Approved

The Institutional Review Board (IRB) of the Harvard University-Area has approved this Continuing Review. **Please note that the approval for this protocol will lapse on 3/15/2018.**

The documents that were finalized for this submission may be accessed through the IRB electronic submission management system at the following link: IRB16-0213.

The IRB made the following determinations:

- Special Populations: None
- Waivers: None
- Risk Determination: No greater than minimal risk
- Research Information Security Level: The research is classified, using Harvard's Data Security Policy, as Level 3 Data.

Please contact me at 6174966348 or abonacossa@hbs.edu with any questions.

Sincerely,

Alain Bonacossa,
Director, Research Administration

HARVARD
Human Research Protection Program

Harvard University-Area
Committee on the Use of Human Subjects
Smith Campus Center, Suite 935
1350 Massachusetts Ave.
Cambridge, MA 02138
IRB Registration - IRB00000109
Federal Wide Assurance - FWA00004837

Notification of Continuing Review Approval

March 9, 2018

Ryan Buell
rbuell@hbs.edu

**Protocol Title:**	Shared Medical Appointments Randomized Trial (SMART)
**Principal Investigator:**	Ryan Buell
**Protocol #:**	IRB16-0213
**Submission #:**	CR16-0213-02
**Funding Source:**	None
**Review Date:**	3/9/2018
**CR Effective Date:**	3/16/2018
**Expiration Date:**	3/15/2019
**IRB Review Type:**	Expedited
**IRB Review Action:**	Approved

The Institutional Review Board (IRB) of the Harvard University-Area has approved this Continuing Review. **Please note that the approval for this protocol will lapse on 3/15/2019.**

The documents that were finalized for this submission may be accessed through the IRB electronic submission management system at the following link: IRB16-0213.

The IRB made the following determinations:
- Special Populations: None
- Waivers: None
- Risk Determination: No greater than minimal risk
- Research Information Security Level: The research is classified, using Harvard's Data Security Policy, as Level 3 Data.

Please contact me at 6174966348 or abonacossa@hbs.edu with any questions.

Sincerely,

Alain Bonacossa,
Senior Director for Research Administration and Behavioral Research Services

HARVARD
Human Research Protection Program

Harvard University-Area
Committee on the Use of Human Subjects
Smith Campus Center, Suite 935
1350 Massachusetts Ave.
Cambridge, MA 02138
IRB Registration - IRB00000109
Federal Wide Assurance - FWA00004837

Notification of Continuing Review Approval

March 17, 2019

Ryan Buell
rbuell@hbs.edu

**Protocol Title:**	Shared Medical Appointments Randomized Trial (SMART)
**Principal Investigator:**	Ryan Buell
**Protocol #:**	IRB16-0213
**Submission #:**	CR16-0213-03
**Funding Source:**	None
**Review Date:**	3/17/2019
**CR Effective Date:**	3/17/2019
**Expiration Date:**	3/16/2020
**IRB Review Type:**	Expedited
**IRB Review Action:**	Approved

The Institutional Review Board (IRB) of the Harvard University-Area has approved this Continuing Review. **Please note that the approval for this protocol will lapse on 3/16/2020.**

The documents that were finalized for this submission may be accessed through the IRB electronic submission management system at the following link: IRB16-0213.

The IRB made the following determinations:
- Risk Determination: No greater than minimal risk
- Research Information Security Level: The research is classified, using Harvard's Data Security Policy, as Level 3 Data.

Please contact me at 6174966348 or abonacossa@hbs.edu with any questions.

Sincerely,

Alain Bonacossa
Senior Director for Research Administration

Harvard University-Area
Committee on the Use of Human Subjects
44-R Brattle, Suite 200 (2nd floor)
Cambridge, MA 02138
IRB Registration - IRB00000109
Federal Wide Assurance - FWA00004837

Notification of Study Closure

April 17, 2020

Ryan Buell
rbuell@hbs.edu

**Protocol Title:**	Shared Medical Appointments Randomized Trial (SMART)
**Principal Investigator:**	Ryan Buell
**Protocol #:**	IRB16-0213
**Submission #:**	CR16-0213-04
**Funding Source:**	None
**IRB Review Date:**	4/17/2020
**IRB Review Action:**	**Closed**

The Institutional Review Board (IRB) of the Harvard University-Area closed this study.

As part of this action:

- The protocol is permanently closed to enrollment.
- All subjects have completed all protocol-related interventions.
- Collection of private identifiable information is completed.
- Analysis of private identifiable information is completed.

Investigators must maintain human research records, including signed and dated consent documents, for at least seven years after study closure. The IRB retains protocol files for the same duration.

If the human research is sponsored, please contact the sponsor before disposing of any records as there may be specific policies related to record retention.

Please contact me at 6174966348 or abonacossa@hbs.edu regarding this protocol or with any questions.

Sincerely,

Alain Bonacossa
Senior Director for Research Administration

London Business School

Regent's Park
London NW1 4SA
United Kingdom

Tel: +44 (0)20 7000 7000
Fax: +44 (0)20 7000 7001
www.london.edu

A Graduate School
of the University
of London

Kamalini Ramdas
Professor of Management Science & Operations
London Business School
Regent's Park
London NW1 4SA

22nd December 2015

Project Title: Shared Medical Appointments

Dear Kamalini,

Thank you for completing the attached ethical review form for the above proposed research project to the Economic and Social Sciences Research Council (ESRC). Please proceed with your application. Should your proposal be successful, given the nature of the project further ethical consideration should be given as required by the funding body. Please do submit any relevant documentation to ethics@london.edu, prior to any work commencing on the project.

A copy of the attached ethical approval application will be retained in the Research & Faculty Office for their records.

Yours sincerely,

Emre Ozdenoren
Chair, Research Ethics Committee

Section 3 – Amendments

Protocol / Protocol related documents Amendment Request and Assessment Form

**IEC Protocol Number:** AEH/PDY/EC/OA/15/2016	
**Protocol Title:**<br>Shared Medical Appointments Randomized Trial (SMART)	
**Principal Investigator and Department:** Dr.S.Kavitha, Glaucoma Services	
**Approved date:** 27.05.2016	**No. of amendment:** 01

State/describe the amendment :type of document/ part of document amended

Extension of Study Recruitment period from Jan ~~16~~ 17 to December 2017.

Reasons for the amendment

Could not complete the recruitment / Sample size not yet met.

Impact of your amendment on your present study at this site: (modifications in the ICD, re-consent of research participants, untoward effects likely to occur because of the amendment or any other)

Have the changes modifications in the amended versions been highlighted/ underlined?

Yes ☐ No ☑

If yes, please submit Annexure – AX 06/SOP 06/V1

With these amendments, do you have any conflicts of interest (financial / non-financial/others) to declare

Yes ☐ No ☑

Name of Principal Investigator: Dr.S.Kavitha

Signature with Date: [signature] 24/01/2017

**Type of review** :- (Decision by the Chairperson/ Member Secretary)<br>Review by Member Secretary/ Chairperson ☐ Review<br>by designated IEC members ☐<br>Full Board discussion and review ☐
**Comments of the reviewer :-**<br>____<br>____
Decision: ☐ Approved ☐ Suggested Recommendation(s)<br>☐ Disapproved ☐ Next full board discussion
Name of IEC Member / Member Secretary /<br>Chairperson reviewing the project:<br><br>Signature with Date:
**Final Decision:** Approved Yes ☑ No ☐<br>If disapproved, reasons for disapproval<br>____<br>Further revision or modification required:
Any Other:
**Signature of the Chairperson/Member Secretary:** [signature]<br><br>**Date:** 09/02/17

Protocol / Protocol related documents Amendment Request and Assessment Form

**IEC Protocol Number:** AEH/PDY/EC/OA/15/2016	
**Protocol Title:**<br>Shared Medical Appointments Randomized Trial (SMART)	
**Principal Investigator and Department:** Dr.S.Kavitha, Glaucoma Services	
**Approved date:** 27.05.2016	**No. of amendment:** 02

State/describe the amendment :type of document/ part of document amended

Questionnaire- Need to add few more questions to the existing exit survey

Reasons for the amendment

Adding these questions to the final survey will help us to understand the patients' perception on shared medical appointment in a better way.

Impact of your amendment on your present study at this site: (modifications in the ICD, re-consent of research participants, untoward effects likely to occur because of the amendment or any other)

Nil

Have the changes modifications in the amended versions been highlighted/ underlined?

Yes ☐ No ☐

If yes, please submit Annexure – AX 06/SOP 06/V1

With these amendments, do you have any conflicts of interest (financial / non-financial/others) to declare

Yes ☐ No ☑

Name of Principal Investigator: Dr.S.Kavitha

Signature with Date: [signature] 24/3/17

**Type of review** :- (Decision by the Chairperson/ Member Secretary)<br>Review by Member Secretary/ Chairperson ☐ Review<br>by designated IEC members ☐<br>Full Board discussion and review ☐
**Comments of the reviewer :-**<br>____<br>____
Decision: ☐ Approved ☐ Suggested Recommendation(s) ☐ Disapproved ☐ Next full board discussion
Name of IEC Member / Member Secretary /<br>Chairperson reviewing the project:<br><br>Signature with Date:
**Final Decision:** Approved Yes ☑ No ☐<br>If disapproved, reasons for disapproval<br>____<br>Further revision or modification required:
Any Other:
**Signature of the Chairperson/Member Secretary:** [signature]<br>**Date:** 01/06/17.

Additional Question We Would Like to Add to the Survey after Trial Appointments 1, 2, 3, 4:

1. (For Both SMA and One on One Patients) How satisfied were you with the length of your waiting time before the final exam stage of your appointment?

a. Very satisfied
b. Satisfied
c. Neutral
d. Dissatisfied
e. Very dissatisfied

Exit Survey

The questions below refer to your glaucoma appointments over the past year.

1. (For Both SMA and One on One Patients) How much at ease did you feel during the interactions?

1 2 3 4 5

1: Very much at ease

5: Not at all at ease

2. (For Both SMA and One on One Patients) To what extent do you agree that your doctor has been fair in his or her dealings with you.

a. Much more than expected
b. More than expected
c. As much as expected
d. Less than expected
e. Much less than expected

3. (For Both SMA and One on One Patients) How caring was the doctor towards you?

a. Much more than expected
b. More than expected
c. As much as expected
d. Less than expected
e. Much less than expected

4. (For Both SMA and One on One Patients) During your appointment, how caring was the doctor towards the other patients?

a. Much more than expected
b. More than expected
c. As much as expected
d. Less than expected
e. Much less than expected

5. (For Both SMA and One on One Patients) During your appointment, how caring was the nurse towards you?

a. Much more than expected
b. More than expected
c. As much as expected
d. Less than expected
e. Much less than expected

6. (For Both SMA and One on One Patients) Did you spend enough time with Dr. Kavitha / Dr. Venkatesh relative to your expectations?

a. Much more than expected
b. More than expected
c. As much as expected
d. Less than expected
e. Much less than expected

7. (For Both SMA and One on One Patients) How Many Minutes of Individual Attention Did You Get from the Doctor in the Final Exam Stage in Each Visit?

8. (For Both SMA and One on One Patients) (Essay) How would you describe your appointment with Dr Kavitha / Venkatesh today?

9. (For Both SMA and One on One Patients) (Essay) What were the best and worst parts of your final examination with Dr. Kavitha/Dr. Venkatesh?

10. (SMA patients only) Over the past year, how often did you interact outside the hospital with people that you met during your shared appointments?

a. Very frequently
b. Frequently
c. Occasionally
d. Rarely
e. Very rarely

11. (For Both SMA and One on One Patients) Over the past Year, to what extent did you coordinate with other patients in your SMA group to determine the date for your follow up appointments?

a. Very frequently
b. Frequently
c. Occasionally
d. Rarely
e. Very rarely

12. (SMA patients only) How satisfied were you with the level of confidentiality?

a. Very satisfied
b. Satisfied
c. Neutral
d. Dissatisfied
e. Very dissatisfied

13. (SMA patients only) How much of what you learned about glaucoma during the final examination came from the doctor?

a. All of what I learned
b. Most of what I learned
c. About half of what I learned
d. Some of what I learned
e. None of what I learned

கண்நீர் அழுத்த நோய்க்கான சிகிச்சையைப் பற்றி தங்களின் புரிதலை கண்டறியும் கேள்வித்தாள்

1. **(பகிர் மருத்துவ பரிசோதனை மற்றும் தனி மருத்துவ பரிசோதனை)** இன்றைய பரிசோதனை நாளில் மருத்துவரை சந்திக்கும் முன் இருந்த காத்திருப்பு நேரம், எந்த அளவிற்கு திருப்தியாக அமைந்தது?

- ☐ மிகவும் திருப்தி
- ☐ திருப்தி
- ☐ நடுநிலை
- ☐ திருப்திகரமாக இல்லை
- ☐ கொஞ்சம் கூட திருப்தி இல்லை

ஆய்வு முடிவில் கேட்கப்படும் வினாக்கள்

கடந்த வருடத்தில் உங்களது கண்நீர் அழுத்த நோயின் பரிசோதனைகள் எவ்வாறு அமைந்தது.

1. **(பகிர் மருத்துவ பரிசோதனை மற்றும் தனி மருத்துவ பரிசோதனை)** உங்களது கலந்து உரையாடலின்
போது எந்த அளவிற்கு தயக்க மின்றி உணர்ந்தீர்கள். 1 2 3 4 5

1: முற்றிலும் தயக்கமில்லை

5: முற்றிலும் தயக்கமாக

2. **(பகிர் மருத்துவ பரிசோதனை மற்றும் தனி மருத்துவ பரிசோதனை)** உங்களை இறுதிக்கட்ட மருத்துவ பரிசோதனை செய்த மருத்துவர் உங்களிடம் எந்த அளவிற்கு தன்மையாக நடந்துகொண்டார்?

- ☐ எதிர்பார்பை விட மிக அதிகமாக
- ☐ எதிர்பார்பை விட அதிகமாக
- ☐ எதிர்பார்த்த அளவிற்கு
- ☐ எதிர்பார்பை விட குறைவாக
- ☐ எதிர்பார்பை விட மிக குறைவாக

3. **(பகிர் மருத்துவ பரிசோதனை மற்றும் தனி மருத்துவ பரிசோதனை)** உங்களிடம் மருத்துவர் எந்த அளவிற்கு அக்கறையுடன்/பரிவாக நடந்துகொண்டார்?

- ☐ எதிர்பார்பை விட மிக அதிகமாக
- ☐ எதிர்பார்பை விட அதிகமாக
- ☐ எதிர்பார்த்த அளவிற்கு
- ☐ எதிர்பார்பை விட குறைவாக
- ☐ எதிர்பார்பை விட மிக குறைவாக

4. **(பகிர் மருத்துவ பரிசோதனை மற்றும் தனி மருத்துவ பரிசோதனை)** உங்கள் இறுதிக்கட்ட மருத்துவ பரிசோதனையின் போது, மருத்துவர் எந்த அளவிற்கு அக்கறையுடன்/பரிவாக மற்ற நோயாளியிடம் நடந்துகொண்டார்?

- ☐ எதிர்பார்பை விட மிக அதிகமாக
- ☐ எதிர்பார்பை விட அதிகமாக
- ☐ எதிர்பார்த்த அளவிற்கு
- ☐ எதிர்பார்பை விட குறைவாக
- ☐ எதிர்பார்பை விட மிக குறைவாக

5. **(பகிர் மருத்துவ பரிசோதனை மற்றும் தனி மருத்துவ பரிசோதனை)** உங்கள் இறுதிக்கட்ட மருத்துவ பரிசோதனையின் போது, எந்த அளவிற்கு செவிலியர்கள், உங்களிடம் அக்கறையுடன்/பரிவாக நடந்துகொண்டார்கள்?

- ☐ எதிர்பார்பை விட மிக அதிகமாக
- ☐ எதிர்பார்பை விட அதிகமாக
- ☐ எதிர்பார்த்த அளவிற்கு
- ☐ எதிர்பார்பை விட குறைவாக
- ☐ எதிர்பார்பை விட மிக குறைவாக

6. **(பகிர் மருத்துவ பரிசோதனை மற்றும் தனி மருத்துவ பரிசோதனை)** நீங்கள் எதிர்பார்த்ததை போல் மரு. கவிதா/மரு. வெங்கடேஷ் உங்களுடன் நேரம் செலவிட்டார்களா?

- ☐ எதிர்பார்பை விட மிக அதிகமாக
- ☐ எதிர்பார்பை விட அதிகமாக
- ☐ எதிர்பார்த்த அளவிற்கு
- ☐ எதிர்பார்பை விட குறைவாக
- ☐ எதிர்பார்பை விட மிக குறைவாக

7. **(பகிர் மருத்துவ பரிசோதனை மற்றும் தனி மருத்துவ பரிசோதனை)** உங்கள் ஒவ்வொரு வருகையின் இறுதி பரிசோதனையின் போது மருத்துவரிடம் இருந்து எத்தனை நிமிடங்கள் தனிப்பட்ட கவனம் கிடைத்தது?

8. **(பகிர் மருத்துவ பரிசோதனை மற்றும் தனி மருத்துவ பரிசோதனை)** இன்றைய பரிசோதனையில் மரு. கவிதா/மரு. வெங்கடேஷ் உடன் நடந்த இறுதி பரிசோதனையை பற்றி உங்களது கருத்து என்ன?

9. **(பகிர் மருத்துவ பரிசோதனை மற்றும் தனி மருத்துவ பரிசோதனை)** உங்களது பரிசோதனையில் மிகவும் நிறைவான (திருப்தியான) பகுதி மற்றும் நிறைவில்லாத (திருப்தியில்லாத) பகுதியை விவரிக்கவும்?

10. **(பகிர் மருத்துவ பரிசோதனை மட்டும்)** கடந்த ஒரு வருடத்தில் உங்களுடன் பகிர் மருத்துவ பரிசோதனையில் பங்கு கொண்டவர்களுடன் இந்த மருத்துவமனை அல்லாத மற்ற இடங்களில் எந்த அளவிற்கு தொடர்பில் (சந்தித்தீர்கள்/பேசினீர்கள்)

- ☐ மிகவும் அதிகமாக
- ☐ அடிக்கடி
- ☐ எப்பொழுதாவது
- ☐ அரிதாக
- ☐ மிகவும் அரிதாக

11. **(பகிர் மருத்துவ பரிசோதனை மற்றும் தனி மருத்துவ பரிசோதனை)** கடந்த ஒரு வருடத்தில் நீங்கள் மருத்துவ பரிசோதனைக்கு வரும் நாளில் உங்கள் குழுவில் உள்ள மற்ற நோயாளிகளும் எந்த அளவிற்கு நேரத்தை கடைபிடிக்க ஒத்துழைக்கீறார்கள்?

- ☐ மிகவும் அதிகமாக
- ☐ அடிக்கடி
- ☐ எப்பொழுதாவது
- ☐ அரிதாக
- ☐ மிகவும் அரிதாக

12. **(பகிர் மருத்துவ பரிசோதனை மட்டும்)** உங்களது நம்பகதன்மை எந்த அளவிற்கு திருப்தியாக அமைந்தது?

- ☐ மிகவும் திருப்தி
- ☐ திருப்தி
- ☐ நடுநிலை
- ☐ திருப்திகரமாக இல்லை
- ☐ கொஞ்சம் கூட திருப்தி இல்லை

13. **(பகிர் மருத்துவ பரிசோதனை மட்டும்)** உங்களது இறுதிகட்ட மருத்துவ பரிசோதனையில், கண்நீர் அழுத்த நோயைப்பற்றி அறிந்து கொண்ட விஷயங்கள் எந்த அளவிற்கு மருத்துவரிடம் இருந்து வந்தது?

- ☐ முற்றிலுமாக
- ☐ கிட்டத்தட்ட எனக்கு தெரிந்தவை எல்லாமே
- ☐ எனக்கு தெரிந்தவற்றில் பாதி
- ☐ ஏதோ ஒரு அளவிற்கு
- ☐ எதுவும் இல்லை

Protocol / Protocol related documents Amendment Request and Assessment Form

IEC Protocol Number: AEH/PDY/EC/OA/15/2016

Protocol Title:

Shared Medical Appointments Randomized Trial (SMART)

Principal Investigator and Department: Dr.S.Kavitha, Glaucoma Services

**Approved date:** 27.05.2016	**No. of amendment:** 03

State/describe the amendment :type of document/ part of document amended

Reasons for the amendment

Extension of study recruitment period (to July 2018) as we could not yet achieve the Sample size

Impact of your amendment on your present study at this site: (modifications in the ICD, re-consent of research participants, untoward effects likely to occur because of the amendment or any other)

Nil

Have the changes modifications in the amended versions been highlighted/ underlined?

Yes ☐ **No** ☐ NA

If yes, please submit Annexure – AX 06/SOP 06/V1

With these amendments, do you have any conflicts of interest (financial / non-financial/others) to declare

Yes ☐ **No** ☑

Name of Principal Investigator: Dr.S.Kavitha

Signature with Date: [signature] 16/3/18

**Type of review** :- (Decision by the Chairperson/ Member Secretary)<br>Review by Member Secretary/ Chairperson ☐<br>Review by designated IEC members ☐<br>Full Board discussion and review ☐
Comments of the reviewer :-<br>______________________________________<br>______________________________________
Decision: ☐ Approved ☐ Suggested Recommendation(s) ☐ Rejected ☐ Next full board discussion
**Name of IEC Member / Member Secretary / Chairperson reviewing the project**:<br>Signature with Date:________________________
**Final Decision:** Approved Yes ☑ No ☐<br>If disapproved, reasons for disapproval<br>______________________________________<br>Further revision or modification required:
Any Other:
**Signature of the Chairperson/Member Secretary:** [signature]<br>**Date:** 16/3/18

Section 4 – Case Report Form

SHARED MEDICAL APPOINTMENTS RANDOMIZED TRIAL (SMART) STUDY

CASE REPORT FORM

Aravind Eye Care System,
Cuddalore Main Road,
Thavalakuppam,
Pondicherry,India

TRIAL VISIT SCHEDULE

	Expected Date	Actual Date
Pre-Trial Visit-1		
Pre-Trial Visit-2		
Pre-Trial Visit-3		
Pre-Trial Visit-4		
Trial Visit-1		
Trial Visit-2		
Trial Visit-3		
Trial Visit-4		
Unscheduled Visit-1		
Unscheduled Visit-2		
Unscheduled Visit-3		
Unscheduled Visit-4		
Post-Trial Visit-1		
Post-Trial Visit-2		
Post-Trial Visit-3		
Post-Trial Visit-4		

ELIGIBILITY WORK SHEET

INCLUSION CRITERIA:

I01. The patient must be a primary glaucoma patient

I02. The patient must not have had more than one surgery in one eye in the past

I03. The patient must not have undergone a tube/ shunt surgery

I04. The patient must not be monocular

I05. The patient should not require surgical intervention in the near future

I06. The patient must not wish to interact with a specific doctor

I07. It is believed that the patient will interact effectively in a group setting

I08. The patient should not be a part of any other existing trial

I09. The patient should not have any vision threatening condition other than glaucoma

EXCLUSION CRITERIA:

E01. The patient who is not primary glaucoma patient.

E02. The patient who has had more than one surgery in one eye in the past

E03. The patient who has undergone a tube shunt surgery

E04. Monocular patients

E05. The patient who requires surgical intervention in the near future

E06. The patient who wishes to interact with a specific doctor

E07. The patient who won't interact effectively in a group setting

E08. The patient who is a part of another trial.

E09. The patient who has any vision threatening condition other than glaucoma.

BASIC DEMOGRAPHIC DETAILS

MR No: ☐☐☐☐☐☐

Unique Identifier for the Patient: ☐☐☐☐☐☐☐

Date of Birth: (Day/Month/Year) ☐☐☐☐☐☐

Sex: (Male-1, Female-2): ☐

Personal Address:

____

____

____

Diagnosis:

RE :____________________________________

LE : ____________________________________

Presence of Systemic Illness:	Yes	No	Duration
Diabetes	☐	☐	
Hypertension	☐	☐	
Cardiac Disease	☐	☐	
Asthma/COPD	☐	☐	

Others (Specify if any) ………………………………………..

Pre-Trial Visit-1

DATE: | | | | | | | | |

Arrival Time in the Glaucoma Clinic ……………………………

Departure Time from the Glaucoma Clinic …………………………….

OBJECTIVE MEASURES:

Number of Years the Patient Has Had Glaucoma …………………………..

Best corrected visual acuity: RE …………………... LE …………………………

Intra Ocular Pressure: RE ………………… LE ………………………..

Number of Medications: RE ……………..... LE ………………………….

Details of Medication:

RE ……………… LE........................

1.Beta blocker
2. Alpha agonist
3.Pilocarpine
4. CAI
5.PG Analogues

Has the patient complied with the prescribed protocol since the last visit?

(Yes or No): …………………………

Optic Nerve Head Examination: RE ……………………… LE ………………………

Other Ocular Co-morbidities: RE ……………….…. LE ………………………

1. Yes 2. No

If Yes, Please mention: RE ……………….. LE ………………………

CONTROL VARIABLES:

Name and Affiliation of the Examining Doctor

……………………………………

Pre-Trial Visit-2

DATE: | | | | | | | | |

Arrival Time in the Glaucoma Clinic ……………………………

Departure Time from the Glaucoma Clinic …………………………….

OBJECTIVE MEASURES:

Number of Years the Patient Has Had Glaucoma …………………………..

Best corrected visual acuity: RE …………………... LE …………………………

Intra Ocular Pressure: RE ………………… LE ………………………..

Number of Medications: RE …………….…. LE ………………………….

Details of Medication:

RE ……………… LE........................

1.Beta blocker
2. Alpha agonist
3.Pilocarpine
4. CAI
5.PG Analogues

Has the patient complied with the prescribed protocol since the last visit?

(Yes or No): …………………………

Optic Nerve Head Examination: RE ……………………… LE ………………………

Other Ocular Co-morbidities: RE ……………….…... LE ………………………

1. Yes 2. No

If Yes, Please mention: RE ……………….. LE ………………………

CONTROL VARIABLES:

Name and Affiliation of the Examining Doctor

……………………………………

Pre-Trial Visit-3

DATE: | | | | | | | | |

Arrival Time in the Glaucoma Clinic ……………………………

Departure Time from the Glaucoma Clinic …………………………….

OBJECTIVE MEASURES:

Number of Years the Patient Has Had Glaucoma …………………………..

Best corrected visual acuity: RE …………………... LE …………………………

Intra Ocular Pressure: RE ………………… LE ………………………..

Number of Medications: RE …………….... LE ………………………….

Details of Medication:

RE ……………… LE.......................

1.Beta blocker
2. Alpha agonist
3.Pilocarpine
4. CAI
5.PG Analogues

Has the patient complied with the prescribed protocol since the last visit?

(Yes or No): …………………………

Optic Nerve Head Examination: RE ……………………… LE ………………………

Other Ocular Co-morbidities: RE ……………….…. LE ………………………

1. Yes 2. No

If Yes, Please mention: RE ……………….. LE ………………………

CONTROL VARIABLES:

Name and Affiliation of the Examining Doctor

……………………………………

Pre-Trial Visit-4

DATE: | | | | | | | |

Arrival Time in the Glaucoma Clinic ……………………………

Departure Time from the Glaucoma Clinic …………………………….

OBJECTIVE MEASURES:

Number of Years the Patient Has Had Glaucoma …………………………..

Best corrected visual acuity: RE …………………... LE …………………………

Intra Ocular Pressure: RE ………………… LE ………………………..

Number of Medications: RE ……………..... LE ………………………….

Details of Medication:

RE ……………… LE.......................

1.Beta blocker
2. Alpha agonist
3.Pilocarpine
4. CAI
5.PG Analogues

Has the patient complied with the prescribed protocol since the last visit?

(Yes or No): …………………………

Optic Nerve Head Examination: RE ……………………… LE ………………………

Other Ocular Co-morbidities: RE ………………...... LE ………………………

1. Yes 2. No

If Yes, Please mention: RE ……………….. LE ………………………

CONTROL VARIABLES:

Name and Affiliation of the Examining Doctor

……………………………………

Trial Visit-1

DATE: | | | | | | | | |

Unique Identity of the Appointment: ....

One-on-one or SMA (by randomization): ....

Number of the Patients in the Appointment: ....

Arrival Time in the Glaucoma Clinic: ....

Start Time of Registration: ....

Time of Entry to the Glaucoma Department: ....

Start Time of Vision Check/Refraction: ....

End Time of Vision Check/Refraction: ....

Start Time for Taking History and Checking Eye Pressure: ....

End Time for Taking History and Checking Eye Pressure: ....

Start Time for the Preliminary Examination: ....

End Time for the Preliminary Examination: ....

Start Time for HFA: ....

End Time for HFA: ....

Start Time for Dilatation: ....

End Time for Dilatation: ....

Start Time of OCT: ....

End Time of OCT: ....

Start Time for Final Examination: ....

End Time for Final Examination: ....

Duration of the Entire Appointment: ....
(From Start of Registration to the End of Final Examination)

Start and End Times for Follow-Up Steps

Start Time for Meeting with Study Coordinator
For Counselling and/or Scheduling a Follow-up Appointment : ....

End Time for Meeting with Study Coordinator
For Counselling and/or scheduling a Follow- up Appointment : ....

Next Appointment On: ....

EFFICACY MEASURES

Patient Perceptions: Administer a brief survey following each appointment, which includes the questions below. Knowledge assessment questions will be used during the pilot period to conduct a sample size calculation, as described in the protocol, and will also be asked throughout the study:

1. Knowledge assessment: Glaucoma is:

a. Contagious disease

b. Hereditary

c. Both

d. None of the above

2. Knowledge assessment: What causes glaucoma?

a. Diabetes

b. Age related

c. Trauma

d. All of the above

3. Knowledge assessment: Glaucoma causes:

a. Loss of visual field/side vision

b. Loss of central vision

c. Eye pain

d. Headache

4. Knowledge assessment: Treatment for glaucoma:

a. Eye drops

b. Laser therapy

c. Surgery

d. All of the above.

5. Knowledge assessment: Glaucoma leads to:

a. High intra-ocular pressure

b. Loss of visual field

c. Optic nerve damage

d. All of the above

6. How satisfied were you with today's appointment:

a. Very satisfied

b. Satisfied

c. Neutral

d. Dissatisfied

e. Very dissatisfied

7. To what extent were your doubts addressed during today's appointment?

a. Fully

b. Almost fully

c. Somewhat

d. Not very well

e. Not at all

8. Relative to your expectations, how much did you learn about glaucoma during today's appointment?

a. Much more than expected

b. More than expected

c. As much as expected

d. Less than expected

e. Much less than expected

9. How well did you understand the doctor's instructions?

a. Fully

b. Almost fully

c. Somewhat

d. Not very well

e. Not at all

10. How likely are you to return for your next appointment?

a. Very likely

b. Likely

c. Somewhat likely

d. Not likely

e. Very unlikely

OBJECTIVE MEASURES:

Best corrected visual acuity: RE …………………...   LE …………………………

Intra Ocular Pressure: RE …………………   LE ………………………..

Number of Medications: RE ……………......   LE ………………………….

Details of Medication:

RE ………………   LE.........................

1.Beta blocker
2. Alpha agonist
3.Pilocarpine
4. CAI
5.PG Analogues

Has the patient complied with the prescribed protocol since the last visit?

(Yes or No): …………………………

Optic Nerve Head Examination: RE ……………………… LE ………………………

Other Ocular Co-morbidities: RE ………………...... LE ………………………

1. Yes 2. No

If Yes, Please mention: RE ……………….. LE ………………………

CONTROL VARIABLES:

Doctor ID for the Appointment

……………………………………

Signature of the Examining Doctor:

……………………………………

Trial Visit-2

DATE:

Unique Identity of the Appointment: ....

One-on-one or SMA : ....

On Trial Time (Yes/No): ....

Number of the Patients in the Appointment: ....

Arrival Time in the Glaucoma Clinic: ....

Start Time of Registration: ....

Time of Entry to the Glaucoma Department: ....

Start Time of Vision Check/Refraction: ....

End Time of Vision Check/Refraction: ....

Start Time for Taking History and Checking Eye Pressure: ....

End Time for Taking History and Checking Eye Pressure: ....

Start Time for the Preliminary Examination: ....

End Time for the Preliminary Examination: ....

Start Time for HFA: ....

End Time for HFA: ....

Start Time for Dilatation: ....

End Time for Dilatation: ....

Start Time of OCT: ....

End Time of OCT: ....

Start Time for Final Examination: ....

End Time for Final Examination: ....

Duration of the Entire Appointment: ....

(From Start of Registration to the End of Final Examination)

Start and End Times for Follow-Up Steps

Start Time for Meeting with Study Coordinator

For Counselling and/or Scheduling a Follow-up Appointment : ....

End Time for Meeting with Study Coordinator

For Counselling and/or Scheduling a Follow- up Appointment : ....

Next Appointment On: ....

EFFICACY MEASURES

Patient Perceptions: Administer a brief survey following each appointment, which includes the questions below. Knowledge assessment questions will be used during the pilot period to conduct a sample size calculation, as described in the protocol, and will also be asked throughout the study:

1. Knowledge assessment: Glaucoma is:

a. Contagious disease

b. Hereditary

c. Both

d. None of the above

2. Knowledge assessment: What causes glaucoma?

a. Diabetes

b. Age related

c. Trauma

d. All of the above

3. Knowledge assessment: Glaucoma causes:

a. Loss of visual field/side vision

b. Loss of central vision

c. Eye pain

d. Headache

4. Knowledge assessment: Treatment for glaucoma:

a. Eye drops

b. Laser therapy

c. Surgery

d. All of the above.

5. Knowledge assessment: Glaucoma leads to:

a. High intra-ocular pressure

b. Loss of visual field

c. Optic nerve damage

d. All of the above

6. How satisfied were you with today's appointment:

a. Very satisfied

b. Satisfied

c. Neutral

d. Dissatisfied

e. Very dissatisfied

7. To what extent were your doubts addressed during today's appointment?

a. Fully

b. Almost fully

c. Somewhat

d. Not very well

e. Not at all

8. Relative to your expectations, how much did you learn about glaucoma during today's appointment?

a. Much more than expected

b. More than expected

c. As much as expected

d. Less than expected

e. Much less than expected

9. How well did you understand the doctor's instructions?

a. Fully

b. Almost fully

c. Somewhat

d. Not very well

e. Not at all

10. How likely are you to return for your next appointment?

a. Very likely

b. Likely

c. Somewhat likely

d. Not likely

e. Very unlikely

OBJECTIVE MEASURES:

Best corrected visual acuity: RE …………………...   LE …………………………

Intra Ocular Pressure: RE …………………   LE ………………………..

Number of Medications: RE …………….....   LE ………………………….

Details of Medication:

RE ………………   LE........................

1.Beta blocker
2. Alpha agonist
3.Pilocarpine
4. CAI
5.PG Analogues

Has the patient complied with the prescribed protocol since the last visit?

(Yes or No): …………………………

Optic Nerve Head Examination: RE ……………………… LE ………………………

Other Ocular Co-morbidities: RE ………………...... LE ………………………

1. Yes 2. No

If Yes, Please mention: RE ……………….. LE ………………………

CONTROL VARIABLES:

Doctor ID for the Appointment

……………………………………

Signature of the Examining Doctor:

……………………………………

Trial Visit-3

DATE: | | | | | | | | |

Unique Identity of the Appointment: ....

One-on-one or SMA: ....

On Trial Time (Yes/No): ....

Number of the Patients in the Appointment: ....

Arrival Time in the Glaucoma Clinic: ....

Start Time of Registration: ....

Time of Entry to the Glaucoma Department: ....

Start Time of Vision Check/Refraction: ....

End Time of Vision Check/Refraction: ....

Start Time for Taking History and Checking Eye Pressure: ....

End Time for Taking History and Checking Eye Pressure: ....

Start Time for the Preliminary Examination: ....

End Time for the Preliminary Examination: ....

Start Time for HFA: ....

End Time for HFA: ....

Start Time for Dilatation: ....

End Time for Dilatation: ....

Start Time of OCT: ....

End Time of OCT: ....

Start Time for Final Examination: ....

End Time for Final Examination: ....

Duration of the Entire Appointment: ....
(From Start of Registration to the End of Final Examination)

Start and End Times for Follow-Up Steps

Start Time for Meeting with Study Coordinator
For Counselling and/or Scheduling a Follow-up Appointment : ....

End Time for Meeting with Study Coordinator
For Counselling and/or scheduling a follow- up Appointment : ....

Next Appointment On: ....

EFFICACY MEASURES

Patient Perceptions: Administer a brief survey following each appointment, which includes the questions below. Knowledge assessment questions will be used during the pilot period to conduct a sample size calculation, as described in the protocol, and will also be asked throughout the study:

1. Knowledge assessment: Glaucoma is:

a. Contagious disease

b. Hereditary

c. Both

d. None of the above

2. Knowledge assessment: What causes glaucoma?

a. Diabetes

b. Age related

c. Trauma

d. All of the above

3. Knowledge assessment: Glaucoma causes:

a. Loss of visual field/side vision

b. Loss of central vision

c. Eye pain

d. Headache

4. Knowledge assessment: Treatment for glaucoma:

a. Eye drops

b. Laser therapy

c. Surgery

d. All of the above.

5. Knowledge assessment: Glaucoma leads to:

a. High intra-ocular pressure

b. Loss of visual field

c. Optic nerve damage

d. All of the above

6. How satisfied were you with today's appointment:

a. Very satisfied

b. Satisfied

c. Neutral

d. Dissatisfied

e. Very dissatisfied

7. To what extent were your doubts addressed during today's appointment?

a. Fully

b. Almost fully

c. Somewhat

d. Not very well

e. Not at all

8. Relative to your expectations, how much did you learn about glaucoma during today's appointment?

a. Much more than expected

b. More than expected

c. As much as expected

d. Less than expected

e. Much less than expected

9. How well did you understand the doctor's instructions?

a. Fully

b. Almost fully

c. Somewhat

d. Not very well

e. Not at all

10. How likely are you to return for your next appointment?

a. Very likely

b. Likely

c. Somewhat likely

d. Not likely

e. Very unlikely

OBJECTIVE MEASURES:

Best corrected visual acuity: RE …………………... LE …………………………

Intra Ocular Pressure: RE ………………… LE ………………………..

Number of Medications: RE ……………..... LE ………………………….

Details of Medication:

RE ……………… LE........................

1.Beta blocker
2. Alpha agonist
3.Pilocarpine
4. CAI
5.PG Analogues

Has the patient complied with the prescribed protocol since the last visit?

(Yes or No): …………………………

Optic Nerve Head Examination: RE ……………………… LE ………………………

Other Ocular Co-morbidities: RE ……………….…. LE ………………………

1. Yes 2. No

If Yes, Please mention: RE ……………….. LE ………………………

CONTROL VARIABLES:

Doctor ID for the Appointment

……………………………………

Signature of the Examining Doctor:

……………………………………

Trial Visit-4

DATE: | | | | | | | | |

One-on-one or SMA: ....

Unique Identity of the Appointment: ....

On Trial Time (Yes/No): ....

Number of the Patients in the Appointment: ....

Arrival Time in the Glaucoma Clinic: ....

Start Time of Registration: ....

Time of Entry to the Glaucoma Department: ....

Start Time of Vision Check/Refraction: ....

End Time of Vision Check/Refraction: ....

Start Time for Taking History and Checking Eye Pressure: ....

End Time for Taking History and Checking Eye Pressure: ....

Start Time for the Preliminary Examination: ....

End Time for the Preliminary Examination: ....

Start Time for HFA: ....

End Time for HFA: ....

Start Time for Dilatation: ....

End Time for Dilatation: ....

Start Time of OCT: ....

End Time of OCT: ....

Start Time for Final Examination: ....

End Time for Final Examination: ....

Duration of the Entire Appointment: ....

(From Start of Registration to the End of Final Examination)

Start and End Times for Follow-Up Steps

Start Time for Meeting with Study Coordinator

For Counselling and/or Scheduling a Follow-up Appointment : ....

End Time for Meeting with Study Coordinator

For Counselling and/or scheduling a follow- up Appointment : ....

EFFICACY MEASURES

Patient Perceptions: Administer a brief survey following each appointment, which includes the questions below. Knowledge assessment questions will be used during the pilot period to conduct a sample size calculation, as described in the protocol, and will also be asked throughout the study. We also have additional questions to assess patient's perceptions on glaucoma care.

1. Knowledge assessment: Glaucoma is:

a. Contagious disease

b. Hereditary

c. Both

d. None of the above

2. Knowledge assessment: What causes glaucoma?

a. Diabetes

b. Age related

c. Trauma

d. All of the above

3. Knowledge assessment: Glaucoma causes:

a. Loss of visual field/side vision

b. Loss of central vision

c. Eye pain

d. Headache

4. Knowledge assessment: Treatment for glaucoma:

a. Eye drops

b. Laser therapy

c. Surgery

d. All of the above.

5. Knowledge assessment: Glaucoma leads to:

a. High intra-ocular pressure

b. Loss of visual field

c. Optic nerve damage

d. All of the above

6. How satisfied were you with today’s appointment:

a. Very satisfied

b. Satisfied

c. Neutral

d. Dissatisfied

e. Very dissatisfied

7. To what extent were your doubts addressed during today’s appointment?

a. Fully

b. Almost fully

c. Somewhat

d. Not very well

e. Not at all

8. Relative to your expectations, how much did you learn about glaucoma during today’s appointment?

a. Much more than expected

b. More than expected

c. As much as expected

d. Less than expected

e. Much less than expected

9. How well did you understand the doctor’s instructions?

a. Fully

b. Almost fully

c. Somewhat

d. Not very well

e. Not at all

10. How likely are you to return for your next appointment?

a. Very likely

b. Likely

c. Somewhat likely

d. Not likely

e. Very unlikely

Additional questions for final patient survey: After the patients fourth visit during the study, the patient will also be asked the following questions:

11. How frequently did you discuss your appointments with others (family members, friends, etc.)?

a. Very frequently

b. Frequently

c. Occasionally

d. Not frequently

e. Never

Patients in the treatment (SMA) condition will additionally be asked:

12. How often did you call other patients that you met during your shared appointments?

a. Very frequently

b. Frequently

c. Occasionally

d. Not frequently

e. Never

13. If they were made available in the future, how likely is it that you would choose to continue using shared medical appointments?

a. Very likely

b. Likely

c. Somewhat likely

d. Not likely

e. Very unlikely

14. How likely would you be to recommend shared medical appointments to a friend, colleague, or relative?

a. Very likely

b. Likely

c. Somewhat likely

d. Not likely

e. Very unlikely

OBJECTIVE MEASURES:

Best corrected visual acuity: RE …………………... LE …………………………

Intra Ocular Pressure: RE ………………… LE ………………………..

Number of Medications: RE ……………..... LE ………………………….

Details of Medication:

RE ……………… LE........................

1.Beta blocker
2. Alpha agonist
3.Pilocarpine
4. CAI
5.PG Analogues

Has the patient complied with the prescribed protocol since the last visit?

(Yes or No): …………………………

Optic Nerve Head Examination: RE ……………………… LE ………………………

Other Ocular Co-morbidities: RE ……………….….. LE ………………………

1. Yes 2. No

If Yes, Please mention: RE ……………….. LE ………………………

CONTROL VARIABLES:

Doctor ID for the Appointment

……………………………………

Signature of the Examining Doctor:

……………………………………

Unscheduled Visit-1

DATE: | | | | | | | | |

Arrival Time in the Glaucoma Clinic ……………………………

Departure Time from the Glaucoma Clinic …………………………….

Reason for Visit...

OBJECTIVE MEASURES:

Best corrected visual acuity: RE …………………... LE …………………………

Intra Ocular Pressure: RE ………………… LE ………………………..

Number of Medications: RE ……………..... LE ………………………….

Details of Medication:

RE ……………… LE.........................

1.Beta blocker
2. Alpha agonist
3.Pilocarpine
4. CAI
5.PG Analogues

Has the patient complied with the prescribed protocol since the last visit?

(Yes or No): …………………………

Optic Nerve Head Examination: RE ……………………… LE ………………………

Other Ocular Co-morbidities: RE ………………..... LE ………………………

1. Yes 2. No

If Yes, Please mention: RE ……………….. LE ………………………

To be continued in the trial (Yes/No): ....

CONTROL VARIABLES:

Name and Affiliation of the Examining Doctor

……………………………………

Unscheduled Visit-2

DATE: | | | | | | | | |

Arrival Time in the Glaucoma Clinic ……………………………

Departure Time from the Glaucoma Clinic …………………………….

Reason for Visit...

OBJECTIVE MEASURES:

Best corrected visual acuity: RE …………………... LE …………………………

Intra Ocular Pressure: RE ………………… LE ………………………..

Number of Medications: RE ……………..... LE ………………………….

Details of Medication:

RE ……………… LE........................

1.Beta blocker
2. Alpha agonist
3.Pilocarpine
4. CAI
5.PG Analogues

Has the patient complied with the prescribed protocol since the last visit?

(Yes or No): …………………………

Optic Nerve Head Examination: RE ……………………… LE ………………………

Other Ocular Co-morbidities: RE ………………...... LE ………………………

1. Yes 2. No

If Yes, Please mention: RE ……………….. LE ………………………

To be continued in the trial (Yes/No): ....

CONTROL VARIABLES:

Name and Affiliation of the Examining Doctor

……………………………………

Unscheduled Visit-3

DATE:								

Arrival Time in the Glaucoma Clinic ……………………………

Departure Time from the Glaucoma Clinic …………………………….

Reason for Visit...

OBJECTIVE MEASURES:

Best corrected visual acuity: RE …………………... LE …………………………

Intra Ocular Pressure: RE ………………… LE ………………………..

Number of Medications: RE …………….... LE ………………………….

Details of Medication:

RE ……………… LE........................

1.Beta blocker
2. Alpha agonist
3.Pilocarpine
4. CAI
5.PG Analogues

Has the patient complied with the prescribed protocol since the last visit?

(Yes or No): …………………………

Optic Nerve Head Examination: RE ……………………… LE ………………………

Other Ocular Co-morbidities: RE ………………..... LE ………………………

1. Yes 2. No

If Yes, Please mention: RE ……………….. LE ………………………

To be continued in the trial (Yes/No): ....

CONTROL VARIABLES:

Name and Affiliation of the Examining Doctor

……………………………………

Unscheduled Visit-4

DATE: | | | | | | | | |

Arrival Time in the Glaucoma Clinic ……………………………

Departure Time from the Glaucoma Clinic …………………………….

Reason for Visit..

OBJECTIVE MEASURES:

Best corrected visual acuity: RE …………………... LE …………………………

Intra Ocular Pressure: RE ………………… LE ………………………..

Number of Medications: RE ……………..... LE ………………………….

Details of Medication:

RE ……………… LE........................

1.Beta blocker
2. Alpha agonist
3.Pilocarpine
4. CAI
5.PG Analogues

Has the patient complied with the prescribed protocol since the last visit?

(Yes or No): …………………………

Optic Nerve Head Examination: RE ……………………… LE ………………………

Other Ocular Co-morbidities: RE ………………...... LE ………………………

1. Yes 2. No

If Yes, Please mention: RE ……………….. LE ………………………

To be continued in the trial (Yes/No): ....

CONTROL VARIABLES:

Name and Affiliation of the Examining Doctor

……………………………………

Post-Trial Visit-1

DATE: | | | | | | | | |

Arrival Time in the Glaucoma Clinic ……………………………

Departure Time from the Glaucoma Clinic …………………………….

OBJECTIVE MEASURES:

Best corrected visual acuity: RE …………………... LE …………………………

Intra Ocular Pressure: RE ………………… LE ………………………..

Number of Medications: RE ……………..... LE ………………………….

Details of Medication:

RE ……………… LE........................

1.Beta blocker
2. Alpha agonist
3.Pilocarpine
4. CAI
5.PG Analogues

Has the patient complied with the prescribed protocol since the last visit?

(Yes or No): …………………………

Optic Nerve Head Examination: RE ……………………… LE ………………………

Other Ocular Co-morbidities: RE ………………...... LE ………………………

1. Yes 2. No

If Yes, Please mention: RE ……………….. LE ………………………

CONTROL VARIABLES:

Name and Affiliation of the Examining Doctor

……………………………………

Post-Trial Visit-2

DATE: | | | | | | | |

Arrival Time in the Glaucoma Clinic ……………………………

Departure Time from the Glaucoma Clinic …………………………….

OBJECTIVE MEASURES:

Best corrected visual acuity: RE …………………... LE …………………………

Intra Ocular Pressure: RE ………………… LE ………………………..

Number of Medications: RE ……………..... LE ………………………….

Details of Medication:

RE ……………… LE........................

1.Beta blocker
2. Alpha agonist
3.Pilocarpine
4. CAI
5.PG Analogues

Has the patient complied with the prescribed protocol since the last visit?

(Yes or No): …………………………

Optic Nerve Head Examination: RE ……………………… LE ………………………

Other Ocular Co-morbidities: RE ………………...... LE ………………………

1. Yes 2. No

If Yes, Please mention: RE ……………….. LE ………………………

CONTROL VARIABLES:

Name and Affiliation of the Examining Doctor

……………………………………

Post-Trial Visit-3

DATE: | | | | | | | | |

Arrival Time in the Glaucoma Clinic ……………………………

Departure Time from the Glaucoma Clinic …………………………….

OBJECTIVE MEASURES:

Best corrected visual acuity: RE …………………... LE …………………………

Intra Ocular Pressure: RE ………………… LE ………………………..

Number of Medications: RE ……………..... LE ………………………….

Details of Medication:

RE ……………… LE........................

1.Beta blocker
2. Alpha agonist
3.Pilocarpine
4. CAI
5.PG Analogues

Has the patient complied with the prescribed protocol since the last visit?

(Yes or No): …………………………

Optic Nerve Head Examination: RE ……………………… LE ………………………

Other Ocular Co-morbidities: RE ………………....... LE ………………………

1. Yes 2. No

If Yes, Please mention: RE ……………….. LE ………………………

CONTROL VARIABLES:

Name and Affiliation of the Examining Doctor

……………………………………

Post-Trial Visit-4

DATE: | | | | | | | | |

Arrival Time in the Glaucoma Clinic ……………………………

Departure Time from the Glaucoma Clinic …………………………….

OBJECTIVE MEASURES:

Best corrected visual acuity: RE …………………... LE …………………………

Intra Ocular Pressure: RE ………………… LE ………………………..

Number of Medications: RE ……………..... LE ………………………….

Details of Medication:

RE ……………… LE........................

1.Beta blocker
2. Alpha agonist
3.Pilocarpine
4. CAI
5.PG Analogues

Has the patient complied with the prescribed protocol since the last visit?

(Yes or No): …………………………

Optic Nerve Head Examination: RE ……………………… LE ………………………

Other Ocular Co-morbidities: RE ………………...... LE ………………………

1. Yes 2. No

If Yes, Please mention: RE ……………….. LE ………………………

CONTROL VARIABLES:

Name and Affiliation of the Examining Doctor

……………………………………

GENERAL COMMENTS

Record any additional relevant information which can not be captured elsewhere in the casebook.

Was there any additional information? NO YES - If yes please specify below:

DATE: ..../................/............................

Details: ....

Signature: ....

DATE: ..../................/............................

Details: ....

Signature: ....

DATE: ..../................/............................

Details: ....

Signature: ....

Section 5 – Patient Information Sheet

PATIENT INFORMATION SHEET

Shared Medical Appointments Randomized Trial (SMART)

This study is conducted at Aravind Eye Hospital, Pondicherry by Dr. S. Kavitha, Glaucoma Consultant and Dr R. Venkatesh, Chief Medical Officer and Head of the Department of Glaucoma Services, in collaboration with Professor Kamilini Ramdas from London Business School and Ryan Buell from Harvard Business School.

You are being invited to take part in this screening study. Before you make a decision, it is important for you to understand why this screening is being done and what it will involve. Please do not hesitate to ask us if there is anything that is not clear or if you would like more information. Take time to decide whether or not you wish to take part.

What is the purpose of the study?

Glaucoma is the second most common cause of blindness and is an important cause of irreversible visual loss in India and the world. Glaucoma is a lifetime disease and taking appropriate medications and treatment, and following up regularly with a glaucoma specialist can slow its progression. The concept of Shared Medical Appointments (SMAs) has been introduced in leading hospitals in the US as a way to improve patient outcomes for lifetime diseases such as diabetes and cardiac disease. The aim of this study is to estimate the impact of using an SMA rather than a traditional one on one appointment during the final examination stage routine follow up appointments at the glaucoma clinic at the Aravind Eye Hospital, Pondicherry. The study will examine how SMAs impact patient outcomes, and also why the effect of SMAs may be different from that of traditional one on one appointments.

What is an Shared Medical Appointment(SMA)?

In an SMA, a patient meets the doctor for the final examination stage of a routine glaucoma follow up appointment in a group, with a target of four other glaucoma patients, rather than one on one. During the SMA, each patient in the group is

examined by the doctor one on one just as in a traditional appointment, while the other patients in the group are watching and listening. After that there is time for question and answer in the group.

Why have I been chosen?

We are asking you to take part in this study because you are a patient of Primary Open Angle Glaucoma or Primary Angle Closure Glaucoma needing review follow-up visits.

Do I have to take part?

It is up to you to decide whether or not to take part. If you do decide to take part you will be given this information sheet to keep and be asked to sign a consent form. If you decide to take part you are still free to withdraw at any time and without giving reason. A decision to withdraw at any time or a decision not to take part, will not affect the standard of care you receive.

What will happen to me if I take part?

If you take part in this screening, your demographic data like age, gender, address, occupation, family history of glaucoma, previous treatment history and history of previous ophthalmic check up will be obtained. Your medical history will be recorded, which includes history of hypertension, cardiac, renal, gastrointestinal, asthma and any other systemic illness along with their treatment history.

Both eyes will be examined for visual acuity, best corrected visual acuity (BCVA), slit lamp bio microscopy of anterior segment, IOP with Goldmann applanation tonometry, undilated fundus evaluation for optic disc assessment, gonioscopy, dilated fundus examination and cases requiring Humphrey field analysis (HFA) and Optical coherence tomography (OCT) will be done whenever it is ordered by the glaucoma consultant. All these procedures are usually done during your normal review visit.

Once you consent to take part in this trial, you will be included in a small group of 4 other patients who have also consented to take part in this trial. Then your group

will be randomized to either one on one or shared medical appointment for your final examination. After the final examination you are requested to answer a questionnaire. This is to assess your understanding about glaucoma. Following this you will need to fix up an appointment for review with the study co-ordinator. If you consent to take part in the study you need to come for 3 additional visits, spaced approximately four months apart. That is, the study duration will be 1 year from your recruitment. The data described above, as well as data from your four most recent visits, as well as the four visits after the end of this study will be shared with the researchers to facilitate the analysis.

During each follow up visit you will undergo the same procedures as described above, and you will experience one on one or SMA final examination stages, depending upon the randomization during your enrolment. During your final visit you will be requested to complete an additional questionnaire to assess your perception of glaucoma care.

The final examination stage of each appointment will also be videotaped, which will be transcribed, and only the coded transcript of your appointments will be retained by researchers. To protect your confidentiality, your name and identity will not be made available to researchers, and after transcription, and a six-year waiting period to accommodate the research publication process, the videotapes will be destroyed.

What are the benefits of taking part?

We hope that all the investigation and examination will add to improvement in your care. Also, the information about SMAs, we get from this study may help us to treat future patients with this condition better .

Will my taking part in this study be kept confidential?

All information that is collected about you during the course of the research will be kept strictly confidential. Any information about you that leaves the hospital will have your name and address removed so that you cannot get recognized from it. The videotapes will also be transcribed from which your name and identity will be removed and only the transcribed text will be used by the researchers. Following transcription,

the videotapes will be destroyed. Until then it will be maintained by the study co-ordinator and will not be made available to the researchers. Although researchers will only receive de-identified data about you, other study participants who may be joining you in a shared medical appointment will be able to identify you and receive information about you during a specific session.

What are all the risks involved in this study?

The study does not pose any risk to the participants. At every step, measures are taken to maintain the confidentiality. Except for the questionnaire, the tests you undergo are part of your routine glaucoma care.

Whom should I contact if I need more information or help?

If you need any more information about the study you can contact Dr. S. Kavitha at: Aravind Eye Hospital, Cuddalore Main Road, Thavalakuppam Pondicherry - 605007. Tel no. 0413 2619100. Additionally, if you have complaints you can report to Institutional Ethics Committee, Aravind Eye Hospital, Thavalakuppam, Pondicherry or you can mail to pdy.irb-office@pondy.aravind.org

Section 6 – Informed Consent Form (English)

INFORMED CONSENT FORM

Subject identification number for this trial

____

Title of the Project:

Shared Medical Appointments Randomized Trial (SMART)

Name of the Principal Investigator: Dr. S. Kavitha

Tel. No: 0413 2619100

I have received the information sheet on the above study and have read / or read to me, and understood the written information.

I have been given the chance to discuss the study and ask questions.

I consent to take part in the study and I am aware that my participation is voluntary.

I understand that I may withdraw at any time without this affecting my future care.

I understand that information collected about me from my participation in this research and sections of any of my medical notes may be looked at by responsible persons (ethics Committee members / regulatory authorities). I give access to these individuals to have access to my records.

I understand that my final examination stage will be videotaped which will be transcribed and used by the investigators. I consent for the same.

I understand that my data will be shared with the researchers identified above from London Business School and Harvard Business School in a way that my identity will not be revealed. I consent for the same.

I understand I will receive a copy of the patient information sheet and the informed consent form.

____________________________________ ____________

Signature / Thumb Impression of subject **Date**

____

Printed name of the subject in capitals

Signature of the Impartial witness Date

Name of the Impartial witness

Signature of the Investigator Date

Name of the Investigator

Section 7 – Informed Consent Form (Tamil)

நோயாளியின் தகவல் படிவம்

பகிர் மருத்துவ பரிசோதனைமுறை -சமவாய்ப்பளிக்கும் ஆய்வு

இந்த ஆய்வானது அரவிந்த் கண் மருத்துவமனைஇ பாண்டிச்சேரியின் கண்நீர் அழுத்த நோய் பிரிவின் மருத்துவரான டாக்டர். எஸ். கவிதா, கண்நீர் அழுத்த பிரிவின் தலைமை அதிகாரி மற்றும் அரவிந்த் கண் மருத்துவமனையின் தலைமை அதிகாரியுமான டாக்டர். ஆர். வெங்கடேஷ், மற்றும் லண்டன் வணிக பள்ளியின் பேராசிரியரான கமாலினி ராமதாஸ் மற்றும் ஹார்வேர்டு வணிக பள்ளியின் ரயன் புல் ஆகியோரின் கூட்டு முயற்ச்சியால் நடத்தப்படுகிறது.

தங்களை இந்த ஆய்வில் பங்கேற்க அழைக்கின்றோம். தாங்கள் பங்கேற்பது குறித்து முடிவு செய்யுமுன் இந்த ஆய்வை பற்றிய விவரங்களை தாங்கள் அறிந்து கொள்வது இன்றியமையாததாகும்.

இந்த தகவல் படிவத்தில் கொடுக்கப்பட்டுள்ள விவரங்கள் தங்களுக்கு போதுமானதாக இல்லை என்றாலோ, தங்களுக்கு மேலும் சில விவரங்கள் தேவவைப்பட்டாலோ ஆய்வாளரிடம் உங்கள் சந்தேகங்களை கேட்டு தெளிவுப்படுத்திக்கொள்ளலாம். நீங்கள் போதுமான அளவு நேரத்தை எடுத்து யோசித்து இந்த ஆய்வில் பங்குபெறுவதை குறித்து முடிவுக்கு வரலாம்.

இந்த ஆய்வின் நோக்கம் என்ன?

கண்நீர் அழுத்த நோயானது உலக அளவில் பார்வை இழப்பிற்கு இரண்டாவது காரணமாக அமைகிறது. இந்தியா மற்றும் உலக அளவில் திரும்பப்பெற இயலாத பார்வை இழப்பை ஏற்படுத்துவதில் கண்நீர் அழுத்தநோய்க்கு முக்கிய பங்கு உண்டு. கண்நீர் அழுத்த நோயானது வாழ்நாள் முழுவதும் தொடரக்கூடிய நோயாகும். எனவே அதற்கு பரிந்துரைக்கப்பட்ட மருந்துகளை மற்றும் சிகிச்சை முறைகளை பின்பற்றுவதால் கண்நீர் அழுத்த நோய் முற்றிய நிலைக்கு செல்லாமல் பாதுகாக்கலாம். பகிர் மருத்துவ பரிசோதனை முறையானது அமெரிக்காவின் சில முன் மாதிரி மருத்துவமனைகளால் வாழ்நாள் முழுவதும் தொடரக்கூடிய நோய்களான நீரிழிவு நோய் மற்றும் இதய நோய்களில் சிகிச்சை விளைவுகளை மேம்படுத்துவதற்காக மேற்கொள்ளப்படுகிறது. இந்த ஆய்வின் முக்கிய குறிக்கோலானது கண்நீர் அழுத்த நோய்க்கான இறுதி பரிசோதனை கட்டத்தில் பகிர் மருத்துவ பரிசோதனையை நாம் காலம் காலமாக பின்பற்றிவரும் ஒருவர் பின் ஒருவராக மருத்துவரை சந்திக்கும் முறையோடு ஒப்பிட்டு பார்ப்பதே ஆகும். இந்த ஆய்வு அரவிந்த் கண் மருத்துவமனை, பாண்டிச்சேரி கண்நீர் அழுத்த நோய் பிரிவில் நடத்தப்படும். இந்த ஆய்வு, பகிர் மருத்துவ பரிசோதனையால் சிகிச்சை விளைவுகளில் ஏற்படும் தாக்கத்தை ஒவ்வொருவராக மருத்துவரை சந்திக்கும் முறையோடு ஒப்பிட்டுப்பார்க்கும்.

பகிர் மருத்துவ பரிசோதனை என்றால் என்ன?

இந்த பகிர் மருத்துவ பரிசோதனை முறையில் கண்நீர் அழுத்த நோய்க்கான இறுதிகட்ட பரிசோதனையின்போது கண்நீர் அழுத்த நோயாளி அவரை போன்றே கண்நீர் அழுத்த நோயால் பாதிக்கப்பட்ட 4 அல்லது 5 பேர் கொண்ட சிறிய குழுவாக மருத்துவரை சந்திப்பார். இவர்கள் குழுவாக இருந்தாலும் மருத்துவர், நோயாளியை ஒவ்வொருவராகவே பரிசோதித்து சிகிச்சை அளிப்பார். ஒருவரை பரிசோதிக்கும்போது குழுவில் உள்ள மற்றவர்கள் அதை பார்க்கவும், கேட்கப்படும்

கேள்விகளையும் அதன் விடைகளையும் கேட்டு அறிந்துகொள்ளவும் இயலும். அனைவரையும் பரிசோதித்த பின்னர் நோயாளி தன் சந்தேகங்களை தனியாகவோ குழுவாகவோ மருத்துவரிடம் கேட்டு நிவர்த்தி செய்து கொள்ளலாம்.

நான் ஏன் இந்த ஆய்விற்காக தேர்ந்தெடுக்கப்பட்டுள்ளேன்?

தாங்கள் திறந்த கோண கண்நீர் அழுத்த நோய் அல்லது குறுகிய கோண கண்நீர் அழுத்த நோய் ஆகிய நோயினால் பாதிக்கப்பட்டு தங்களுக்கு தொடர் சிகிச்சை தேவைப்படுவதால் உங்களை இந்த ஆய்விற்காக தேர்ந்தெடுத்துள்ளோம்.

நான் இந்த ஆய்வில் பங்குபெற வேண்டுமா?

இந்த ஆய்வில் பங்குபெறுவதை குறித்து நீங்கள் தன்னிச்சையாக முடிவு செய்யலாம். நீங்கள் பங்குபெற நினைத்தால் உங்களிடம் இந்த தகவல் படிவம் கொடுக்கப்படும். இதை படித்து ஆய்வை பற்றி முற்றிலும் அறிந்தபின் நீங்கள் ஒப்புதல் படிவத்தில் கையெழுத்திடவேண்டும். நீங்கள் இந்த ஆய்வில் பங்கேற்க முடிவு செய்தபின்னரும் எந்த ஒரு நேரத்திலும் இந்த ஆய்வில் இருந்து விளகிக்கொள்ள உங்களுக்கு உரிமை உண்டு. அதற்கான காரணத்தை நீங்கள் எங்களுக்கு தெரிவிக்கவேண்டிய அவசியம் இல்லை. நீங்கள் இந்த ஆய்வில் இருந்து விலகிக்கொன்டாலும் தங்களுக்கு அளிக்கப்படும் சிகிச்சை முறையில் எந்த ஒரு பாதிப்பும் ஏற்படாது என்பதை தெரிவித்துக்கொள்கிறோம்.

நான் இந்த ஆய்வில் பங்கேற்க முடிவு செய்தால் மேற்கொண்டு எனக்கு என்ன நடக்கும்?

நீங்கள் இந்த ஆய்வில் பங்கேற்றால் தங்களின் வயது, பாலினம், முகவரிஇ தொழில், கண்நீர் அழுத்த நோய் பற்றிய குடும்ப வரலாறு, முந்தய கண் சிகிச்சை விவரங்கள் ஆகியவை கேட்டரியப்படும். உங்கள் மருத்துவ வரலாறும் குறிப்பாக இரத்த அழுத்தம், இருதய நோய், ஆஸ்துமா, சிறுநீரக நோய் போன்ற நோய்களின் விவரங்களும் அதற்குறிய சிகிச்சை விவரங்களும் கேட்டு அறியப்படும். அதை தொடர்ந்து பொதுவாக செய்யப்படும் கண் பரிசோதனைகளான பார்வை அளவு பரிசோதனை, கண்நீர் அழுத்தம், ஸ்லிட் லேம்பு கொண்டு கண்ணின் முற்பகுதி பரிசோதனை, கண்ணின் கோணப்பகுதியை கோனியோஸ்கோப்பி என்ற கருவி கொண்டு பரிசோதனை பின்னர் கண்களின் பாப்பாவை மருந்துமூலம் விரித்து கண் பார்வை நரம்பு பரிசோதனை ஆகியவை மேற்கொள்ளப்படும். மருத்துவரின் பரிந்துரைப்படி தேவைப்பட்டால் பக்க பார்வை பரிசோதனை மற்றும் ஓ.சி.டி எனப்படும் பார்வை நரம்பு ஸ்கேனும் எடுக்கப்படும். மேற்சொல்லப்பட்ட எல்லா பரிசோதனைகளையும் தாங்கள் ஒவ்வொரு முறையும் மறுபரிசோதனைக்கு வரும்போது வழக்கமாக செய்யப்படும் பரிசோதனைகளே ஆகும்.

நீங்கள் இந்த ஆய்வில் பங்குபெற ஒப்புதல் அளித்தபின் உங்களைப் போன்றே ஒப்புதல் அளித்த மேலும் 4 கண்நீர் அழுத்த நோயாளிகளுடன் சேர்த்து சிறு குழு ஏற்படுத்தப்படும். பின்னர் உங்கள் குழுவானது இறுதிகட்ட பரிசோதனையின்போது பகிர் மருத்துவ முறைக்கோ அல்லது ஒவ்வொருவராக மருத்துவரை சந்திக்கும் முறைக்கோ சமவாய்ப்பு அளிக்கப்படும் முறையில் தேர்ந்தெடுக்கப்படும். இறுதிகட்ட பரிசோதனைக்கு பின்னர் தாங்கள் ஒரு கேள்வி தாளுக்கு விடை அளிக்கும்படி கேட்டுக்கொள்ளப்படுவர். இந்த கேள்வித்தாளில் உள்ள கேள்விகள் கண்நீர் அழுத்த

நோய் பற்றிய உங்களின் புரிதலை கண்டறிவதற்காக கொடுக்கப்படுகிறது. அதன் பின்னர் தாங்கள் மறுபரிசோதனைக்கான பதிவை ஆய்வு ஒருங்கினைப்பாளரிடம் பெற்று கொள்ள வேண்டும். இந்த ஆய்வு காலமானது தங்கள் நுழைவிலிருந்து ஒரு வருடம் வரை மேற்கொள்ளப்படும். நீங்கள் இந்த ஒரு வருடத்தில் நான்கு மாத இடைவேளையில் மூன்று முறை மறுபரிசோதனைக்கு வர அழைக்கப்படுவீர்கள். மேலே குறிப்பிட்ட புள்ளி விவரங்கள் தவிர, ஆய்வில் பங்கெடுப்பதற்கு முந்தய நான்கு பரிசோதனைகளின் விவரங்களும் ஆய்விற்கு பின் நடக்கும் நான்கு பரிசோதனைகளின் விவரங்களும் உங்கள் மருத்துவப் பதிவேட்டிலிருந்து எடுக்கப்படும்.

ஒவ்வொரு மறுபரிசோதனையின்போதும் நீங்கள் 4 அல்லது 5 பேர் கொண்ட குழுவில் தனித்தனியாகவோ அல்லது பகிர் மருத்துவ முறையிலோ இறுதிக்கட்ட பரிசோதனையை மேற்கொள்வீர்கள். முதன் பரிசோதனையின்போது உங்களுக்கு எந்த முறை பரிந்துரைக்கப்பட்டதோ ஒவ்வொரு மறுபரிசோதனையின்போதும் அதே முறையே பின்பற்றப்படும். ஆய்வின் இறுதி மறுபரிசோதனையின்போது கண்நீர் அழுத்த நோய்க்கான சிகிச்சையை பற்றிய தங்களின் புரிதலை கண்டறிய ஒரு சிறிய கேள்வித்தாள் கொடுக்கப்படும். தாங்கள் அதை பூர்த்தி செய்யவேண்டும்.

ஒவ்வொரு பரிசோதனையின்போதும் இறுதிகட்ட பரிசோதனை மட்டும் வீடியோ பதிவு செய்யப்படும். அந்த வீடியோ பதிவு நாடாக்களில் உள்ள விவரங்கள் மட்டும் பெயர்த்தெழுதப்படும். அந்த பெயர்த்தெழுதப்பட்ட விவரங்கள் மட்டுமே ஆய்வாளரிடம் அளிக்கப்படும். அதுவரை ஒளிநாடாக்கள் ஆய்வு ஒருங்கினைப்பாளரிடம் பாதுகாப்பாக கொடுத்துவைக்கப்படும். ஆய்வு முற்றிலுமாக முடிந்தபின் ஒளிநாடாக்களின் விவரங்கள் பெயர்த்தெழுதியப்பின்னரும் ஆறு வருடம் வரை அதாவது இறுதி ஆய்வு அறிக்கை வெளியாகும் வரை ஒளிநாடாக்கள் அரவிந்த் கண் மருத்துவமனையில் பாதுகாப்பாக வைக்கப்படும், அதன் பின்னர் அவை அழிக்கப்படும். இதன் மூலம் உங்கள் பெயரோடு உங்களை கண்டரியப்படும் எந்த ஒரு விவரமோ ஆய்வளர்களை சென்றடையாது, உங்களின் இரகசியத்தன்மை பாதுகாக்கப்படும். பெயர்த்தெழுதுதல் அந்த துறையில் தேர்ந்தவர்களிடம் கொடுக்கப்படும்.

இந்த ஆய்வில் பங்குபெறுவதால் என்ன பயன்கள் கிடைக்கும்?

உங்களுக்கு அளிக்கப்படும் பரிசோதனை முறைகளில் தேர்ச்சி அடைய இந்த ஆய்வு பயன்படும் என நம்புகிறோம். மேலும் பகிர் மருத்துவ பரிசோதனை முறையை பற்றி இந்த ஆய்வில் நாம் கண்டறியும் விவரங்கள் எதிர்காலத்தில் கண்நீர் அழுத்த நோயாளிகளுக்கு அளிக்கப்படும், சிகிச்சை முறைகளை மேம்படுத்த பயன்படும் என நம்புகிறோம்.

இந்த ஆய்வில் பங்குபெறுவதால் என்னை பற்றிய விவரங்கள் எவ்வாறு நம்பகமாக பாதுகாக்கப்படும்?

இந்த ஆய்வில் உங்களைப்பற்றி சேகரிக்கப்படும் அனைத்து விவரங்களும் இரகசியமாக பாதுகாக்கப்படும். மருத்துவமனையில் இருந்து வெளியே அனுப்பப்படும் எந்த ஒரு தகவலில் இருந்தும் உங்களுடைய பெயரும் முகவரியும் நீங்கப்படும், ஆகையால் அந்த விவரங்கள் உங்களுடையது என யாராலும் கண்டுபிடிக்க இயலாது. வீடியோ பதிவிலிருந்தும் கூட உங்களது பெயரும் உங்களை கண்டறியும் மற்ற விவரங்களும் முற்றிலும் நீங்கப்படும். வீடியோ பதிவுகளை ஆய்வு காலம் முடியும்வரை ஆய்வு ஒருங்கினைப்பாளர் பாதுகாத்து வைத்திருப்பார், மேலும்

அது ஆய்வளர்களிடம் ஒப்படைக்கபடமாட்டாது. ஆய்வளர்களை சென்றடையும் தகவல்களில் உங்களை கண்டறியும் விவரங்கள் முற்றிலுமாக நீங்கப்பட்டாலும் பகிர் மருத்துவ பரிசோதனையின் போது உங்கள் குழுவில் இருக்கும் மற்றவர்கள் உங்களைப்பற்றிய விவரங்களை அறிய வாய்ப்புள்ளது.

இந்த ஆய்வில் பங்கேற்பதால் எனக்கு ஆபத்து எதுவும் நிகழ வாய்ப்புள்ளதா?

இந்த ஆய்வில் பங்கெடுப்பதால் உங்களுக்கு எந்த ஒரு ஆபத்தும் ஏற்படாது. ஒவ்வொரு நிலையிலும் உங்களைப்பற்றிய விவரங்கள் நம்பகமாக, இரகசியமாக பாதுகாக்கப்படும். இந்த ஆய்வில் கொடுக்கப்படும் கேள்வித்தாள் தவிற மற்ற பரிசோதனைகள் உங்களுடைய கண்நீர் அழுத்த நோய் பரிசோதனையில் வழக்கமாக செய்யப்படும் பரிசோதனைகளே ஆகும்.

எனக்கு மேலும் சில தகவல்கள் தேவைபட்டால் அல்லது உதவி தேவைபட்டால் நான் யாரை தொடர்பு கொள்ள வேண்டும்?

நீங்கள் அரவிந்த் கண் மருத்துவமனை கடலூர் மெயின்ரோடு தவளக்குப்பம் புதுச்சேரி – 605007 -> இங்கு உள்ள கண்நீர் அழுத்த நோய் பிரிவு மருத்துவர் டாக்டர். எஸ். கவிதா அவர்களை தொடர்பு கொள்ளலாம். தொலைப்பேசி எண்- 0413 2619100. மேலும் தேவைப்பட்டால் அரவிந்த் கண் மருத்துவமனையில், மருத்துவ ஆய்வுகளில் முறைப்பாடுகளை மேற்பார்க்கும் தனி குழுவிடம் (எதிக்ஸ் கமிட்டி) உங்களுக்கு ஏதெனும் பிரச்சனை இருந்தால் முறையீடு செய்யலாம் அல்லது pdy.irb-office@pondy.aravind.org என்ற மின் அஞ்சல் வழியாக தொடர்பு கொள்ளலாம்.

ஒப்புதல் படிவம்

ஆய்வுஎண் : ____________________

ஆய்வின் பெயர் :

பகிர் மருத்துவ பரிசோதனைமுறை -சமவாய்ப்பளிக்கும் ஆய்வு

ஆய்வாளரின் பெயர் : டாக்டர் எஸ். கவிதா

தொலைபேசி எண் : 0413 261 9100

நான் இந்த ஆய்வைப் பற்றியதகவல் படிவத்தைப் பெற்றுக் கொண்டேன். தகவல் படிவத்திலுள்ள அனைத்தையும் படித்தேன் அல்லது புரிந்துக் கொண்டேன்.

எனக்கு ஆய்வை விவாதிக்க மற்றும் கேள்விகள் கேட்க வாய்ப்பு அளிக்கப்பட்டது.

1. இந்த ஆய்வில் பங்குகொள்ள யாருடைய வற்புறுத்தலும் இல்லாமல் எனது மனப்பூர்வமான சம்மதத்தினை தெரிவித்துக்கொள்கிறேன்.

எந்த நேரத்திலும், எக்காரணத்தினாலும் என்னுடைய சிகிச்சை தடைபெறாதவகையில் நான் இந்த ஆய்விலிருந்து விலகலாம் என்பதை தெரிந்துக்கொண்டேன்.

எனது மருத்துவக் குறிப்புகள் மற்றும் சிகிச்சை சம்பந்தமான செய்திகள் அனைத்தையும் இந்த ஆராய்ச்சிக்கு பயன்படுத்திக்கொள்ள எனது முழு சம்மதத்தினை தெரிவித்துக்கொள்கிறேன்.

என்னுடைய இறுதிக்கட்ட பரிசோதனையானது ஒளிப்பதிவு செய்யப்படும் என்பதை அறிவேன். அந்த ஒளிப்பதிவிலிருந்து பெயர்த்தெழுதப்பட்ட விவரங்கள் மட்டுமே ஆய்வாளர்களை சென்றடையும் என அறிவேன். இதற்கு நான் ஒப்புதல் அளிக்கிறேன்.

இந்த ஆய்வின் மூலம் பெறப்படும் என்னுடைய விவரங்களில் இருந்து என்னுடைய பெயரும் என்னை கண்டறியும் விவரங்களும் நீக்கப்பட்ட பின்னரே அவை லண்டன் வணிகப் பள்ளி மற்றும் ஹார்வேர்டு வணிகப் பள்ளிக்கு அனுப்பப்படும் என அறிவேன். இதற்கு நான் ஒப்புதல் அளிக்கிறேன்.

எனக்கு நோயாளியின் தவகல் படிவத்தின் நகல் கொடுக்கப்படும்இ என்பதைபுரிந்துக் கொண்டேன்.

நோயாளியின் கையெழுத்து / நோயாளியின் கைரேகை | தேதி

நோயாளியின் பெயர்

ஆய்வாளரின் கையெழுத்து | தேதி

ஆய்வாளரின் பெயர்

சாட்சியாளர் கையெழுத்து | தேதி

சாட்சியாளர் பெயர்
