# Supplementary material for: Evidence from the first Shared Medical Appointments (SMAs) randomised controlled trial in India: SMAs increase the satisfaction, knowledge, and medication compliance of patients with glaucoma
Source: PLOS Glob Public Health. 2023 Jul 20;3(7):e0001648. doi: 10.1371/journal.pgph.0001648 (PMC10358908; doi:10.1371/journal.pgph.0001648)
Supplement: S1 Fig — (PDF) [file pgph.0001648.s005.pdf]

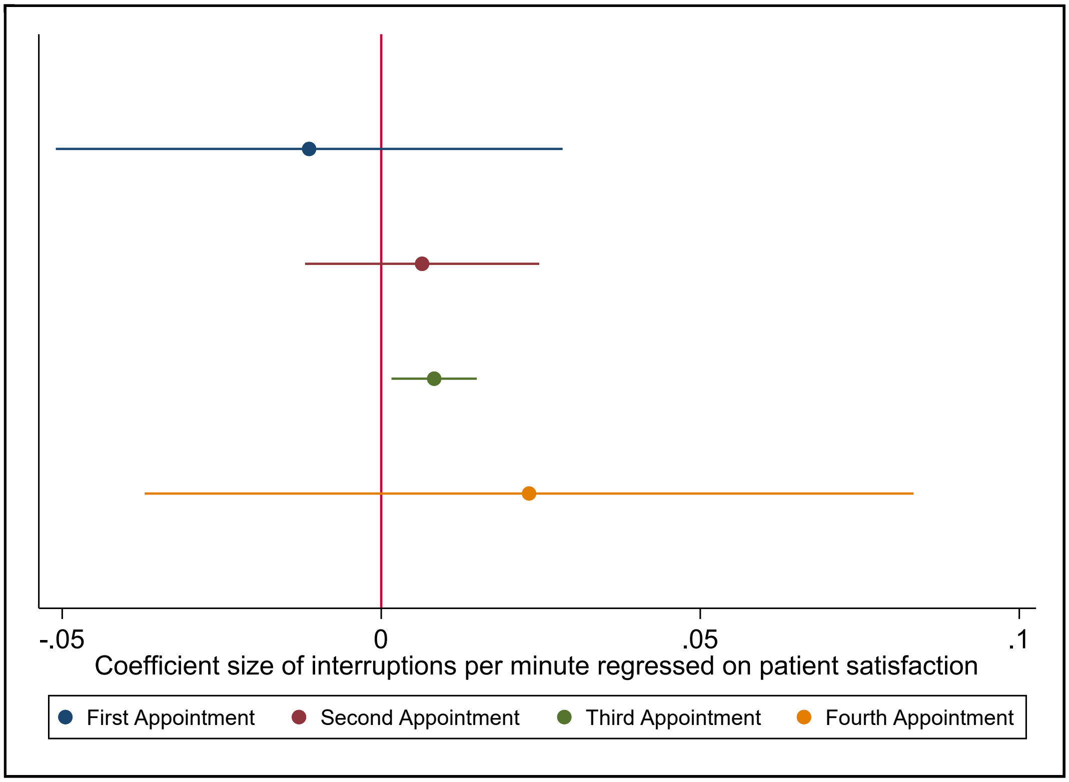

S1 Fig: Coefficient size of interruptions per minute regressed on patient satisfaction

For each trial appointment, lines represent the coefficient size for interruptions per minute when patient satisfaction is modelled and estimated as a function of gestures per minute, demographic variables, and doctor specific differences.
