# Supplementary material for: Evidence from the first Shared Medical Appointments (SMAs) randomised controlled trial in India: SMAs increase the satisfaction, knowledge, and medication compliance of patients with glaucoma
Source: PLOS Glob Public Health. 2023 Jul 20;3(7):e0001648. doi: 10.1371/journal.pgph.0001648 (PMC10358908; doi:10.1371/journal.pgph.0001648)
Supplement: S1 Table — (PDF) [file pgph.0001648.s007.pdf]

	Attended (N = 1000)	Declined to Participate (N = 34)	Difference
**Demographic Variables:**			
Age	62.039 (9.335)	62.971 (9.762)	-0.932
Proportion of Male Patients	0.602 (0.490)	0.676 (0.475)	-0.074
Urban	0.612 (0.488)	0.676 (0.475)	-0.064
Education Level†	2.568 (1.210)	2.441 (1.050)	0.127
**Medical Variables:**			
**Proportion of Glaucoma Types**			
Primary Open Angle Glaucoma (POAG)	0.740 (0.438)	0.764 (0.431)	-0.024
Primary Angle Closure Disease (PACD)	0.224 (0.417)	0.205 (0.410)	0.018
Ocular Hypertension (OHT)	0.010 (0.100)	0.000 (0.000)	0.010
Pseudoexfoliation Glaucoma (PXF Glaucoma)	0.026 (0.159)	0.029 (0.171)	-0.003
**Proportion of Comorbidities**			
Diabetes	0.373 (0.484)	0.353 (0.485)	0.020
Hypertension	0.365 (0.482)	0.265 (0.448)	0.100
Cardiac Disease	0.037 (0.189)	0.059 (0.239)	-0.022
Asthma / Chronic Obstructive Pulmonary Disease (COPD)	0.019 (0.137)	0.000 (0.000)	0.019
Other Chronic Diseases	0.007 (0.083)	0.000 (0.000)	0.007

Data are mean (SD). There were significant differences between both groups in Proportion of Ocular Hypertension, Asthma COPD and Other Chronic Diseases, allowing for differences in variances across groups (all p values <0.001). † Education is scaled as: Illiterate (1), Primary School Education (2), Secondary School Education (3), Undergraduate Education (4), Postgraduate Education (5).

S1 Table: Characteristics of patients who attended versus declined to participate
