# Supplementary material for: Evidence from the first Shared Medical Appointments (SMAs) randomised controlled trial in India: SMAs increase the satisfaction, knowledge, and medication compliance of patients with glaucoma
Source: PLOS Glob Public Health. 2023 Jul 20;3(7):e0001648. doi: 10.1371/journal.pgph.0001648 (PMC10358908; doi:10.1371/journal.pgph.0001648)
Supplement: S2 Table — (PDF) [file pgph.0001648.s008.pdf]

Knowledge Assessment	Satisfaction Assessment
**1. Glaucoma is:**<br><br>a. A contagious disease<br>b. Hereditary<br>c. Both<br>d. None of the above	**1. How satisfied were you with today's appointment?**<br><br>a. Very satisfied<br>b. Satisfied<br>c. Neutral<br>d. Dissatisfied<br>e. Very dissatisfied
**2. What causes glaucoma?**<br><br>a. Diabetes<br>b. Age related<br>c. Trauma<br>d. All of the above	**2. To what extent were your doubts addressed during today's appointment?**<br><br>a. Fully<br>b. Almost fully<br>c. Somewhat<br>d. Not very well<br>e. Not at all
**3. Glaucoma causes:**<br><br>a. Loss of visual field/side vision<br>b. Loss of central vision<br>c. Eye pain<br>d. Headache	**3. Relative to your expectations, how much did you learn about glaucoma during today's appointment?**<br><br>a. Much more than expected<br>b. More than expected<br>c. As much as expected<br>d. Less than expected<br>e. Much less than expected
**4. Treatment for glaucoma:**<br><br>a. Eye drops<br>b. Laser therapy<br>c. Surgery<br>d. All of the above	**4. How well did you understand the doctor's instructions?**<br><br>a. Fully<br>b. Almost fully<br>c. Somewhat<br>d. Not very well<br>e. Not at all
**5. Glaucoma leads to:**<br><br>a. High intraocular pressure<br>b. Loss of visual field<br>c. Optic nerve damage<br>d. All of the above	**5. How likely are you to return for your next appointment?**<br><br>a. Very likely<br>b. Likely<br>c. Somewhat likely<br>d. Unlikely<br>e. Very unlikely

Answers to the survey questions which measure knowledge of the patients are coded by giving a score of 1 if the answer is true and 0 otherwise. However, for the satisfaction level, a continuum is preferable. Therefore, for the questions related to the satisfaction level of patients, we use a Likert-scale. We describe each level of the scale so as to obtain precise estimates. For example, how satisfied a patient is with today's appointment is pre-specified as: Very Dissatisfied (1); Dissatisfied (2); Neutral (3); Satisfied (4); Very Satisfied (5). Other questions about satisfaction are coded in a similar manner.

S2 Table: Knowledge and satisfaction assessment
