# Supplementary material for: Evidence from the first Shared Medical Appointments (SMAs) randomised controlled trial in India: SMAs increase the satisfaction, knowledge, and medication compliance of patients with glaucoma
Source: PLOS Glob Public Health. 2023 Jul 20;3(7):e0001648. doi: 10.1371/journal.pgph.0001648 (PMC10358908; doi:10.1371/journal.pgph.0001648)
Supplement: S3 Table — (PDF) [file pgph.0001648.s009.pdf]

Primary Outcomes	SMA	One-On-One	Difference in Means (95% CI) ¶	p value
Satisfaction with the Appointment	4.956 (0.242)	4.919 (0.328)	0.037 (0.018–0.055)	0.0001
Satisfaction with Doubts Addressed	4.976 (0.191)	4.939 (0.331)	0.037 (0.019–0.055)	<0.0001
Satisfaction with Learning	4.901 (0.355)	4.808 (0.579)	0.092 (0.061–0.124)	<0.0001
Satisfaction with Understanding Instructions	4.980 (0.174)	4.973 (0.207)	0.007 (-0.005–0.020)	0.266
Knowledge Level	3.418 (1.301)	3.264 (1.423)	0.154 (0.065–0.243)	0.0007
Medication Compliance Rate†	0.968 (0.188)	0.951 (0.230)	0.017 (0.001–0.033)	0.037
Intention to Return	4.989 (0.118)	4.985 (0.154)	0.004 (-0.005–0.013)	0.387
Probability of Returning within 30 Days†	0.876 (0.375)	0.886 (0.337)	-0.010 (-0.037–0.017)	0.472

Data are mean (SD). ¶ Continuous outcomes were analysed by means of linear regression. Binary outcomes were analysed by means of logistic regression. We controlled for the patient's biological sex, age, urbanity, education level, and the presence of comorbidities as well as an indicator variable denoting the identity of the doctor. 95% confidence intervals were constructed with errors clustered at the patient level. † We examined data from 2nd, 3th and 4th appointment of the trial for Medication Compliance Rate and Probability of Returning within 30 Days outcomes. For these outcomes, values in the 1st appointment are treatment independent. For other measures, we use data from all four trial appointments.

S3 Table: Primary outcomes with controls
