# Supplementary material for: Evidence from the first Shared Medical Appointments (SMAs) randomised controlled trial in India: SMAs increase the satisfaction, knowledge, and medication compliance of patients with glaucoma
Source: PLOS Glob Public Health. 2023 Jul 20;3(7):e0001648. doi: 10.1371/journal.pgph.0001648 (PMC10358908; doi:10.1371/journal.pgph.0001648)
Supplement: S5 Table — (PDF) [file pgph.0001648.s011.pdf]

Prespecified Subgroup‡	SMA	One-On-One	Difference (95% CI) ¶	p value for Interaction
**Gender**				
Female (N^{SMA} = 166, N^{1-1} = 148)	-0.539 (3.037)	-0.537 (2.979)	-0.002 (-0.671–0.667)	0.903
Male (N^{SMA} = 239, N^{1-1} = 266)	-0.274 (3.417)	-0.327 (3.332)	0.053 (-0.538–0.644)	
**Location**				
Rural (N^{SMA} = 160, N^{1-1} = 168)	-0.266 (3.482)	-0.012 (3.186)	-0.254 (-0.980–0.472)	0.320
Urban (N^{SMA} = 245, N^{1-1} = 246)	-0.459 (3.120)	-0.669 (3.203)	0.210 (-0.351–0.770)	
**Education Level**				
Illiterate (N^{SMA} = 44, N^{1-1} = 48)	-0.693 (2.880)	-0.427 (3.210)	-0.266 (-1.528–0.995)	0.029
Primary School (N^{SMA}= 238, N^{1-1} = 230)	-0.532 (3.366)	-0.415 (3.190)	-0.116 (-0.712–0.479)	
Secondary School (N^{SMA}= 16, N^{1-1} = 26)	0.031 (2.918)	0.308 (2.867)	-0.276 (-2.136–1.584)	
Undergraduate (N^{SMA}= 66, N^{1-1} = 51)	0.583 (3.304)	-1.225 (3.711)	1.809 (0.502–3.116)***	
Postgraduate (N^{SMA}= 41, N^{1-1} = 59)	-0.902 (2.916)	0.068 (2.889)	-0.970 (-2.142–0.202)	
**Age**				
≤65 (N^{SMA}= 253, N^{1-1} = 246)	-0.411 (3.427)	-0.278 (3.341)	-0.133 (-0.728–0.463)	0.399
>65 (N^{SMA}= 152, N^{1-1} = 168)	-0.336 (2.987)	-0.583 (3.005)	0.248 (-0.412–0.908)	
**Comorbidities**				
Diabetes (N^{SMA}= 150, N^{1-1} = 158)	-0.170 (3.064)	-0.547 (3.026)	0.377 (-0.306–1.061)	0.883
Hypertension (N^{SMA}= 138, N^{1-1} = 158)	-0.290 (3.199)	-0.459 (3.203)	0.169 (-0.565–0.903)	
Cardiac Disease (N^{SMA}= 16, N^{1-1} = 15)	-0.031 (3.006)	-1.033 (3.384)	1.002 (-1.352–3.356)	
Asthma / Chronic Obstructive Pulmonary Disease (COPD) (N^{SMA}= 6, N^{1-1} = 7)	-0.500 (1.803)	-0.357 (3.637)	-0.143 (-3.540–3.255)	
Other Chronic Diseases (N^{SMA}= 2 , N^{1-1} = 4)	1.250 (1.531)	0.125 (3.528)	1.125 (-4.195–6.445)	
**Overall** (N^{SMA}= 405, N^{1-1} = 414)	-0.383 (3.265)	-0.402 (3.208)	0.019 (-0.425–0.464)	

Data are mean (SD). There was a significant difference between both groups in Undergraduate (p value <0.001). As documented in Table S14, baseline intraocular pressure level was significantly higher among undergraduate patients in the one-on-one condition. When baseline level differences are controlled in the undergraduate subgroup analysis, the differences observed above reduce to insignificance. ‡ In each row, the sample sizes N^{SMA} and N^{1-1} denote the number of observations – across all relevant appointments – at the subgroup level in question (e.g., Female or Male), in SMAs and 1-1s respectively. ¶ Change in Intraocular Pressure was analysed by means of linear regression. 95% confidence intervals were constructed using the errors clustered at patient level. *** p<0.01, ** p<0.05, * p<0.1 – these p values are associated with the treatment effect within each subgroup.

S5 Table: Change in intraocular pressure (ΔIOP), in prespecified subgroups
