# Supplementary material for: Evidence from the first Shared Medical Appointments (SMAs) randomised controlled trial in India: SMAs increase the satisfaction, knowledge, and medication compliance of patients with glaucoma
Source: PLOS Glob Public Health. 2023 Jul 20;3(7):e0001648. doi: 10.1371/journal.pgph.0001648 (PMC10358908; doi:10.1371/journal.pgph.0001648)
Supplement: S6 Table — (PDF) [file pgph.0001648.s012.pdf]

Prespecified Subgroup‡	SMA	One-On-One	Difference (95% CI) ¶	p value for Interaction
**Gender**				
Female (N^{SMA} = 166, $N^{1\text{-}1}$ = 148)	-0.530 (3.096)	-0.547 (3.008)	0.017 (-0.669–0.703)	0.003
Male (N^{SMA} = 239, $N^{1\text{-}1}$ = 266)	-0.282 (3.405)	-0.320 (3.455)	0.038 (-0.572–0.647)	
**Location**				
Rural (N^{SMA} = 160, $N^{1\text{-}1}$ = 168)	-0.239 (3.473)	-0.037 (3.222)	-0.202 (-0.932–0.528)	0.256
Urban (N^{SMA} = 245, $N^{1\text{-}1}$ = 246)	-0.489 (3.108)	-0.639 (3.273)	0.150 (-0.419–0.719)	
**Education Level**				
Illiterate (N^{SMA} = 44, $N^{1\text{-}1}$ = 48)	-0.722 (3.069)	-0.400 (3.113)	-0.322 (-1.629–0.985)	0.001
Primary School (N^{SMA}= 238, $N^{1\text{-}1}$ = 230)	-0.541 (3.298)	-0.406 (3.176)	-0.135 (-0.722–0.452)	
Secondary School (N^{SMA}= 16, $N^{1\text{-}1}$ = 26)	0.275 (2.934)	0.158 (3.225)	0.117 (-1.936–2.170)	
Undergraduate (N^{SMA}= 66, $N^{1\text{-}1}$ = 51)	0.562 (3.643)	-1.197 (3.729)	1.759 (0.324–3.194)**	
Postgraduate (N^{SMA}= 41, $N^{1\text{-}1}$ = 59)	-0.887 (3.371)	0.057 (2.836)	-0.944 (-2.273–0.386)	
**Age**				
≤65 (N^{SMA}= 253, $N^{1\text{-}1}$ = 246)	-0.435 (3.407)	-0.254 (3.406)	-0.180 (-0.784–0.423)	0.015
>65 (N^{SMA}= 152, $N^{1\text{-}1}$ = 168)	-0.314 (3.054)	-0.602 (2.995)	0.288 (-0.385–0.961)	
**Comorbidities**				
Diabetes (N^{SMA}= 150, $N^{1\text{-}1}$ = 158)	-0.101 (3.129)	-0.613 (3.037)	0.511 (-0.189–1.212)	0.000†
Hypertension (N^{SMA}= 138, $N^{1\text{-}1}$ = 158)	-0.265 (3.265)	-0.481 (3.227)	0.216 (-0.530–0.963)	
Cardiac Disease (N^{SMA}= 16, $N^{1\text{-}1}$ = 15)	0.110 (3.723)	-1.184 (4.297)	1.294 (-2.058–4.646)	
Asthma / Chronic Obstructive Pulmonary Disease (COPD) (N^{SMA}= 6, $N^{1\text{-}1}$ = 7)	-0.333 (1.228)	-0.500 (3.870)	0.167 (-2.677–3.011)	
Other Chronic Diseases† (N^{SMA}= 2 , $N^{1\text{-}1}$ = 4)	1.250 (1.767)	0.125 (3.326)	n/a	
**Overall** (N^{SMA}= 405, $N^{1\text{-}1}$ = 414)	-0.379 (3.255)	-0.405 (3.270)	0.026 (-0.424–0.476)	

Data are mean (SD). There was a significant difference between both groups in Undergraduate (p value <0.001). As documented in Table S14, baseline intraocular pressure level was significantly higher among undergraduate patients in the one-on-one condition. When baseline level differences are controlled in the undergraduate subgroup analysis, the differences observed above reduce to insignificance. ‡ In each row, the sample sizes N^{SMA} and $N^{1\text{-}1}$ denote the number of observations – across all relevant appointments – at the subgroup level in question (e.g., Female or Male), in SMAs and 1-1s respectively. ¶ Change in Intraocular Pressure was analysed by means of linear regression. 95% confidence intervals were constructed using the errors clustered at patient level. We controlled for the patient's biological sex, age, urbanity, education level, and the presence of comorbidities as well as an indicator variable denoting the identity of the doctor. *** p<0.01, ** p<0.05, * p<0.1 – these p values are associated with the treatment effect within each subgroup. † Due to lack of outcome variation in some of the subgroups, it was only possible to calculate the chi-square p value for the interaction using the subgroups for which we could derive difference and confidence intervals from regression models. Mean (SD) derived from summary statistics when the model could not have been estimated due to lack of variation in one or two arms of one subgroup and resulted in n/a as the difference in means.

S6 Table: Change in intraocular pressure (ΔIOP), in prespecified subgroups with controls
