# Supplementary material for: Evidence from the first Shared Medical Appointments (SMAs) randomised controlled trial in India: SMAs increase the satisfaction, knowledge, and medication compliance of patients with glaucoma
Source: PLOS Glob Public Health. 2023 Jul 20;3(7):e0001648. doi: 10.1371/journal.pgph.0001648 (PMC10358908; doi:10.1371/journal.pgph.0001648)
Supplement: S7 Table — (PDF) [file pgph.0001648.s013.pdf]

Prespecified Subgroup‡	SMA	One-On-One	Difference (95% CI) ¶	p value for Interaction
**Gender**				
Female (N^{SMA}= 179, N^{1-1} = 154)	0.011 (0.062)	0.009 (0.034)	0.002 (-0.009–0.012)	0.784
Male (N^{SMA}= 246, N^{1-1} = 275)	0.002 (0.058)	0.002 (0.036)	0.000 (-0.009–0.008)	
**Location**				
Rural (N^{SMA}= 167, N^{1-1} = 174)	0.003 (0.047)	0.003 (0.036)	0.000 (-0.009–0.009)	0.766
Urban (N^{SMA}= 258, N^{1-1} = 255)	0.008 (0.067)	0.006 (0.035)	0.002 (-0.008–0.011)	
**Education Level**				
Illiterate (N^{SMA}= 46, N^{1-1} = 50)	0.030 (0.094)	0.008 (0.025)	0.022 (-0.007–0.050)	0.253
Primary School (N^{SMA}= 250, N^{1-1} = 239)	0.000 (0.051)	0.004 (0.038)	-0.005 (-0.013–0.003)	
Secondary School (N^{SMA}= 18, N^{1-1} = 27)	0.004 (0.024)	0.007 (0.034)	-0.003 (-0.021–0.014)	
Undergraduate (N^{SMA} = 69, N^{1-1} = 54)	0.006 (0.066)	0.001 (0.029)	0.004 (-0.013–0.022)	
Postgraduate (N^{SMA}= 42, N^{1-1} = 59)	0.018 (0.057)	0.006 (0.036)	0.012 (-0.008–0.031)	
**Age**				
≤65 (N^{SMA}= 267, N^{1-1} = 254)	0.003 (0.049)	0.007 (0.032)	-0.003 (-0.010–0.004)	0.165
>65 (N^{SMA}= 158, N^{1-1} = 175)	0.010 (0.075)	0.002 (0.039)	0.007 (-0.006–0.021)	
**Comorbidities**				
Diabetes (N^{SMA}= 159, N^{1-1} = 164)	-0.002 (0.056)	0.011 (0.037)	-0.013 (-0.024–-0.003)**	0.016†
Hypertension (N^{SMA}= 149, N^{1-1} = 165)	0.011 (0.048)	0.008 (0.037)	0.003 (-0.007–0.013)	
Cardiac Disease (N^{SMA}= 16, N^{1-1} = 17)	-0.011 (0.053)	0.012 (0.022)	-0.023 (-0.052–0.006)	
Asthma / Chronic Obstructive Pulmonary Disease (COPD) (N^{SMA}= 6, N^{1-1} = 7)	-0.021 (0.051)	-0.014 (0.020)	-0.007 (-0.054–0.041)	
Other Chronic Diseases† (N^{SMA}= 2 , N^{1-1} = 4)	0.000 (0.000)	0.000 (0.020)	n/a	
**Overall** (N^{SMA}= 425, N^{1-1} = 429)	0.006 (0.060)	0.005 (0.035)	0.001 (-0.006–0.008)	

Data are mean (SD). The effect of shared medical appointments in reducing optic nerve head cup-to-disk ratio was significant among patients with Diabetes (P value = 0.014). In Table S15, we compare starting optic nerve head cup-to-disk ratio, and see no significant differences among patients with diabetes who were randomly assigned to experience one-on-one and shared medical appointments. ‡ In each row, the sample sizes N^{SMA} and N^{1-1} denote the number of observations – across all relevant appointments – at the subgroup level in question (e.g., Female or Male), in SMAs and 1-1s respectively. ¶ Change in Optic Nerve Head Cup-to-Disk Ratio was analysed by means of linear regression. 95% confidence intervals were constructed, clustering errors at the patient level. *** p<0.01, ** p<0.05, * p<0.1 – these p values are associated with the treatment effect within each subgroup. † Due to lack of outcome variation in some of the subgroups, it was only possible to calculate the chi-square p value for the interaction using the subgroups for which we could derive difference and confidence intervals from regression models. Mean (SD) derived from summary statistics when the model could not have been estimated due to lack of variation in one or two arms of one subgroup and resulted in n/a as the difference in means.

S7 Table: Change in optic nerve head cup-to-disk ratio (ΔONH), in prespecified subgroups
