# Supplementary material for: Evidence from the first Shared Medical Appointments (SMAs) randomised controlled trial in India: SMAs increase the satisfaction, knowledge, and medication compliance of patients with glaucoma
Source: PLOS Glob Public Health. 2023 Jul 20;3(7):e0001648. doi: 10.1371/journal.pgph.0001648 (PMC10358908; doi:10.1371/journal.pgph.0001648)
Supplement: S10 Table — (PDF) [file pgph.0001648.s016.pdf]

Prespecified Subgroup‡	SMA	One-On-One	Difference (95% CI) ¶	p value for Interaction
**Gender**				
Female (N^{SMA}= 766, N^{1-1} = 677)	4.965 (0.201)	4.918 (0.353)	0.047 (0.016–0.078)***	0.034
Male (N^{SMA}= 1051, N^{1-1} = 1162)	4.949 (0.270)	4.921 (0.302)	0.028 (0.004–0.052)**	
**Location**				
Rural (N^{SMA}= 709, N^{1-1} = 735)	4.946 (0.266)	4.913 (0.317)	0.033 (0.003–0.064)**	0.025
Urban (N^{SMA}= 1108, N^{1-1} = 1104)	4.961 (0.223)	4.925 (0.327)	0.036 (0.012–0.059)***	
**Education Level**				
Illiterate (N^{SMA}= 191, N^{1-1} = 229)	4.976 (0.176)	4.911 (0.369)	0.065 (0.008–0.121)**	0.362
Primary School (N^{SMA}= 1082, N^{1-1} = 1018)	4.951 (0.260)	4.917 (0.329)	0.034 (0.009–0.060)***	
Secondary School (N^{SMA}= 75, N^{1-1} = 108)	4.987 (0.148)	4.926 (0.309)	0.062 (-0.014–0.137)	
Undergraduate (N^{SMA}= 292, N^{1-1} = 232)	4.944 (0.233)	4.937 (0.275)	0.007 (-0.037–0.051)	
Postgraduate (N^{SMA}= 177, N^{1-1} = 252)	4.958 (0.276)	4.926 (0.316)	0.032 (-0.026–0.091)	
**Age**				
≤65 (N^{SMA}= 1140, N^{1-1} = 1095)	4.963 (0.204)	4.929 (0.317)	0.033 (0.011–0.056)***	0.053
>65 (N^{SMA}= 677, N^{1-1} = 744)	4.946 (0.295)	4.904 (0.339)	0.042 (0.008–0.075)**	
**Comorbidities**				
Diabetes (N^{SMA}= 680, N^{1-1} = 701)	4.951 (0.266)	4.904 (0.348)	0.047 (0.014–0.080)***	0.202†
Hypertension (N^{SMA}= 632, N^{1-1} = 702)	4.968 (0.217)	4.913 (0.320)	0.055 (0.026–0.084)***	
Cardiac Disease (N^{SMA}= 71, N^{1-1} = 66)	4.926 (0.358)	4.898 (0.221)	0.029 (-0.093–0.150)	
Asthma / Chronic Obstructive Pulmonary Disease (COPD) (N^{SMA}= 37, N^{1-1} = 29)	4.967 (0.228)	4.835 (0.369)	0.133 (-0.050–0.315)	
Other Chronic Diseases† (N^{SMA}= 8 , N^{1-1} = 19)	5.000 (0.000)	5.000 (0.000)	n/a	
**Overall** (N^{SMA}= 1817, N^{1-1} = 1839)	4.956 (0.242)	4.919 (0.328)	0.037 (0.018–0.055)***	

Data are mean (SD). ‡ In each row, the sample sizes N^{SMA} and N^{1-1} denote the number of observations – across all relevant appointments – at the subgroup level in question (e.g., Female or Male), in SMAs and 1-1s respectively. † Due to lack of outcome variation in some of the subgroups, it was only possible to calculate the chi-square p value for the interaction using the subgroups for which we could derive difference and confidence intervals from regression models. Mean (SD) derived from summary statistics when the model could not have been estimated due to lack of variation in one or two arms of one subgroup and resulted in n/a as the difference in means. ¶ Satisfaction with the Appointment was analysed by means of linear regression. 95% confidence intervals were constructed, clustering errors at the patient level. We controlled for the patient's biological sex, age, urbanity, education level, and the presence of comorbidities as well as an indicator variable denoting the identity of the doctor. *** p<0.01, ** p<0.05, *p<0.1– these p values are associated with the treatment effect within each subgroup.

S10 Table: Satisfaction with the appointment, in prespecified subgroups with controls
