# Supplementary material for: Evidence from the first Shared Medical Appointments (SMAs) randomised controlled trial in India: SMAs increase the satisfaction, knowledge, and medication compliance of patients with glaucoma
Source: PLOS Glob Public Health. 2023 Jul 20;3(7):e0001648. doi: 10.1371/journal.pgph.0001648 (PMC10358908; doi:10.1371/journal.pgph.0001648)
Supplement: S13 Table — (PDF) [file pgph.0001648.s019.pdf]

Prespecified Subgroup‡	SMA	One-On-One	Difference (95% CI) ¶	p value for Interaction
**Gender**				
Female (N^{SMA}= 766, N^{1-1} = 676)	4.914 (0.338)	4.839 (0.545)	0.075 (0.027–0.123)***	0.512
Male (N^{SMA}= 1051, N^{1-1} = 1162)	4.889 (0.370)	4.793 (0.597)	0.096 (0.055–0.137)***	
**Location**				
Rural (N^{SMA}= 709, N^{1-1} = 735)	4.907 (0.326)	4.812 (0.565)	0.095 (0.047–0.142)***	0.801
Urban (N^{SMA}= 1108, N^{1-1} = 1103)	4.894 (0.376)	4.808 (0.589)	0.087 (0.045–0.128)***	
**Education Level**				
Illiterate (N^{SMA}= 191, N^{1-1} = 229)	4.953 (0.246)	4.913 (0.328)	0.040 (-0.015–0.096)	0.392
Primary School (N^{SMA}= 1082, N^{1-1} = 1017)	4.894 (0.349)	4.793 (0.627)	0.101 (0.057–0.145)***	
Secondary School (N^{SMA}= 75, N^{1-1} = 108)	4.867 (0.444)	4.824 (0.561)	0.043 (-0.107–0.192)	
Undergraduate (N^{SMA}= 292, N^{1-1} = 232)	4.880 (0.424)	4.780 (0.606)	0.100 (0.007–0.193)**	
Postgraduate (N^{SMA}= 177, N^{1-1} = 252)	4.921 (0.325)	4.806 (0.507)	0.115 (0.036–0.195)***	
**Age**				
≤65 (N^{SMA}= 1140, N^{1-1} = 1094)	4.911 (0.328)	4.820 (0.585)	0.091 (0.052–0.131)***	0.832
>65*** (N^{SMA}= 677, N^{1-1} = 744)	4.879 (0.398)	4.794 (0.570)	0.085 (0.034–0.135)***	
**Comorbidities**				
Diabetes (N^{SMA} = 680, N^{1-1} = 700)	4.904 (0.350)	4.817 (0.602)	0.087 (0.035–0.139)***	0.273
Hypertension (N^{SMA}= 632, N^{1-1} = 701)	4.908 (0.358)	4.790 (0.600)	0.118 (0.065–0.171)***	
Cardiac Disease (N^{SMA}= 71, N^{1-1} = 66)	4.944 (0.272)	4.712 (0.660)	0.232 (0.054–0.409)**	
Asthma / Chronic Obstructive Pulmonary Disease (COPD) (N^{SMA}= 37, N^{1-1} = 29)	4.973 (0.165)	4.759 (0.576)	0.214 (-0.017–0.446)*	
Other Chronic Diseases (N^{SMA}= 8 , N^{1-1} = 19)	5.000 (0.000)	4.947 (0.223)	0.053 (-0.073–0.178)	
**Overall** (N^{SMA}= 1817, N^{1-1} = 1838)	4.899 (0.357)	4.810 (0.579)	0.090 (0.059–0.121)***	

Data are mean (SD). ‡ In each row, the sample sizes N^{SMA} and N^{1-1} denote the number of observations – across all relevant appointments – at the subgroup level in question (e.g., Female or Male), in SMAs and 1-1s respectively. ¶ Satisfaction with Learning was analysed by means of linear regression. 95% confidence intervals were constructed, clustering errors at the patient level. *** p<0.01, ** p<0.05, *p<0.1 – these p values are associated with the treatment effect within each subgroup.

S13 Table: Satisfaction with learning, in prespecified subgroups
