# Supplementary material for: Evidence from the first Shared Medical Appointments (SMAs) randomised controlled trial in India: SMAs increase the satisfaction, knowledge, and medication compliance of patients with glaucoma
Source: PLOS Glob Public Health. 2023 Jul 20;3(7):e0001648. doi: 10.1371/journal.pgph.0001648 (PMC10358908; doi:10.1371/journal.pgph.0001648)
Supplement: S17 Table — (PDF) [file pgph.0001648.s023.pdf]

Prespecified Subgroup‡	SMA	One-On-One	Difference (95% CI) ¶	p value for Interaction
**Gender**				
Female (N^{SMA}= 766, N^{1-1} = 677)	3.299 (1.346)	3.233 (1.495)	0.066 (-0.082–0.214)	0.122
Male (N^{SMA}= 1051, N^{1-1} = 1162)	3.500 (1.310)	3.287 (1.491)	0.214 (0.097–0.331)***	
**Location**				
Rural (N^{SMA}= 709, N^{1-1} = 735)	3.343 (1.447)	3.174 (1.396)	0.169 (0.021–0.316)**	0.713
Urban (N^{SMA}= 1108, N^{1-1} = 1104)	3.462 (1.260)	3.329 (1.544)	0.133 (0.016–0.251)**	
**Education Level**				
Illiterate (N^{SMA}= 191, N^{1-1} = 229)	2.969 (1.337)	2.886 (1.271)	0.082 (-0.172–0.336)	0.563
Primary School (N^{SMA}= 1082, N^{1-1} = 1018)	3.388 (1.245)	3.188 (1.434)	0.201 (0.085–0.316)***	
Secondary School (N^{SMA}= 75, N^{1-1} = 108)	3.600 (1.394)	3.417 (1.499)	0.183 (-0.251–0.618)	
Undergraduate (N^{SMA}= 292, N^{1-1} = 232)	3.568 (1.358)	3.591 (1.468)	-0.022 (-0.269–0.225)	
Postgraduate (N^{SMA}= 177, N^{1-1} = 252)	3.734 (1.323)	3.571 (1.507)	0.163 (-0.109–0.435)	
**Age**				
≤65 (N^{SMA}= 1140, N^{1-1} = 1095)	3.416 (1.373)	3.327 (1.490)	0.089 (-0.030–0.208)	0.121
>65 (N^{SMA}= 677, N^{1-1} = 744)	3.415 (1.285)	3.179 (1.480)	0.236 (0.092–0.381)***	
**Comorbidities**				
Diabetes (N^{SMA}= 680, N^{1-1} = 701)	3.371 (1.299)	3.252 (1.440)	0.118 (-0.027–0.263)	0.004
Hypertension (N^{SMA}= 632, N^{1-1} = 702)	3.453 (1.311)	3.259 (1.515)	0.193 (0.041–0.345)**	
Cardiac Disease (N^{SMA}= 71, N^{1-1} = 66)	3.479 (1.107)	2.924 (1.425)	0.555 (0.110–0.999)**	
Asthma / Chronic Obstructive Pulmonary Disease (COPD) (N^{SMA}= 37, N^{1-1} = 29)	3.378 (1.276)	3.276 (1.841)	0.103 (-0.740–0.945)	
Other Chronic Diseases (N^{SMA}= 8, N^{1-1} = 19)	3.000 (0.551)	3.684 (0.946)	-0.684 (-1.398–0.029)**	
**Overall** (N^{SMA}= 1817, N^{1-1} = 1839)	3.416 (1.340)	3.267 (1.492)	0.149 (0.057–0.241)***	

Data are mean (SD). ‡ In each row, the sample sizes N^{SMA} and N^{1-1} denote the number of observations – across all relevant appointments – at the subgroup level in question (e.g., Female or Male), in SMAs and 1-1s respectively. ¶ Patient Knowledge outcome was analysed by means of linear regression. 95% confidence intervals were constructed, clustering errors at the patient level. ***p<0.01, ** p<0.05, *p<0.1 – these p values are associated with the treatment effect within each subgroup.

S17 Table: Patient knowledge level, in prespecified subgroups
