# Supplementary material for: Evidence from the first Shared Medical Appointments (SMAs) randomised controlled trial in India: SMAs increase the satisfaction, knowledge, and medication compliance of patients with glaucoma
Source: PLOS Glob Public Health. 2023 Jul 20;3(7):e0001648. doi: 10.1371/journal.pgph.0001648 (PMC10358908; doi:10.1371/journal.pgph.0001648)
Supplement: S18 Table — (PDF) [file pgph.0001648.s024.pdf]

Prespecified Subgroup‡	SMA	One-On-One	Difference (95% CI) ¶	p value for Interaction
**Gender**				
Female (N^{SMA}= 766, N^{1-1} = 677)	3.294 (1.298)	3.239 (1.444)	0.054 (-0.091–0.200)	0.026
Male (N^{SMA}= 1051, N^{1-1} = 1162)	3.500 (1.295)	3.287 (1.423)	0.213 (0.099–0.327)***	
**Location**				
Rural (N^{SMA}= 709, N^{1-1} = 735)	3.347 (1.407)	3.170 (1.360)	0.177 (0.031–0.322)**	0.110
Urban (N^{SMA}= 1108, N^{1-1} = 1104)	3.469 (1.212)	3.322 (1.460)	0.147 (0.034–0.260)**	
**Education Level**				
Illiterate (N^{SMA}= 191, N^{1-1} = 229)	2.975 (1.427)	2.881 (1.321)	0.094 (-0.189–0.377)	0.055
Primary School (N^{SMA}= 1082, N^{1-1} = 1018)	3.388 (1.247)	3.188 (1.404)	0.200 (0.086–0.314)***	
Secondary School (N^{SMA}= 75, N^{1-1} = 108)	3.549 (1.420)	3.452 (1.573)	0.098 (-0.379–0.574)	
Undergraduate (N^{SMA}= 292, N^{1-1} = 232)	3.552 (1.390)	3.611 (1.493)	-0.060 (-0.320–0.201)	
Postgraduate (N^{SMA}= 177, N^{1-1} = 252)	3.748 (1.205)	3.562 (1.453)	0.186 (-0.075–0.446)	
**Age**				
≤65 (N^{SMA}= 1140, N^{1-1} = 1095)	3.419 (1.336)	3.324 (1.423)	0.095 (-0.021–0.212)	0.217
>65 (N^{SMA}= 677, N^{1-1} = 744)	3.418 (1.242)	3.176 (1.422)	0.242 (0.102–0.383)***	
**Comorbidities**				
Diabetes (N^{SMA}= 680, N^{1-1} = 701)	3.371 (1.256)	3.252 (1.412)	0.118 (-0.026–0.263)	0.000
Hypertension (N^{SMA}= 632, N^{1-1} = 702)	3.439 (1.255)	3.271 (1.478)	0.168 (0.018–0.318)**	
Cardiac Disease (N^{SMA}= 71, N^{1-1} = 66)	3.411 (1.105)	2.997 (1.407)	0.414 (-0.080–0.908)*	
Asthma / Chronic Obstructive Pulmonary Disease (COPD) (N^{SMA}= 37, N^{1-1} = 29)	3.570 (0.994)	3.032 (1.257)	0.538 (-0.137–1.212)	
Other Chronic Diseases (N^{SMA}= 8, N^{1-1} = 19)	5.292 (0.392)	2.719 (0.254)	2.573 (2.091–3.054)***	
**Overall** (N^{SMA}= 1817, N^{1-1} = 1839)	3.418 (1.301)	3.264 (1.423)	0.154 (0.065–0.243)***	

Data are mean (SD). ‡ In each row, the sample sizes N^{SMA} and N^{1-1} denote the number of observations – across all relevant appointments – at the subgroup level in question (e.g., Female or Male), in SMAs and 1-1s respectively. ¶ Patient Knowledge outcome was analysed by means of linear regression. 95% confidence intervals were constructed, clustering errors at the patient level. We controlled for the patient's biological sex, age, urbanity, education level, and the presence of comorbidities as well as an indicator variable denoting the identity of the doctor. ***p<0.01, ** p<0.05, *p<0.1 – these p values are associated with the treatment effect within each subgroup.

S18 Table: Patient knowledge level, in prespecified subgroups with controls
