# Supplementary material for: Evidence from the first Shared Medical Appointments (SMAs) randomised controlled trial in India: SMAs increase the satisfaction, knowledge, and medication compliance of patients with glaucoma
Source: PLOS Glob Public Health. 2023 Jul 20;3(7):e0001648. doi: 10.1371/journal.pgph.0001648 (PMC10358908; doi:10.1371/journal.pgph.0001648)
Supplement: S27 Table — (PDF) [file pgph.0001648.s033.pdf]

Prespecified Subgroup‡	SMA	One-On-One	Difference (95% CI) ¶	p value for Interaction
**Gender**				
Female (N^{SMA}= 211, N^{1-1} = 185)	15.765 (3.300)	15.486 (3.554)	0.279 (-0.402–0.960)	0.554
Male (N^{SMA}= 287, N^{1-1} = 313)	15.049 (3.447)	15.029 (3.100)	0.020 (-0.507–0.547)	
**Location**				
Rural (N^{SMA}= 190, N^{1-1} = 196)	15.032 (3.390)	14.982 (3.103)	0.049 (-0.601–0.700)	0.707
Urban (N^{SMA}= 308, N^{1-1} = 302)	15.550 (3.397)	15.339 (3.388)	0.211 (-0.329–0.750)	
**Education Level**				
Illiterate (N^{SMA}= 52, N^{1-1} = 64)	15.452 (3.387)	15.094 (3.374)	0.358 (-0.892–1.608)	0.054
Primary School (N^{SMA}= 297, N^{1-1} = 275)	15.466 (3.473)	15.271 (3.279)	0.195 (-0.359–0.750)	
Secondary School (N^{SMA}= 21, N^{1-1} = 28)	15.810 (4.131)	13.750 (3.022)	2.060 (-0.086–4.205)**	
Undergraduate (N^{SMA}= 79, N^{1-1} = 65)	14.671 (2.972)	15.777 (3.332)	-1.106 (-2.157–-0.055)	
Postgraduate (N^{SMA}= 49, N^{1-1} = 66)	15.459 (3.262)	15.045 (3.151)	0.414 (-0.787–1.615)	
**Age**				
≤65 (N^{SMA}= 310, N^{1-1} = 296)	15.684 (3.574)	15.537 (3.454)	0.147 (-0.414–0.707)	0.916
>65 (N^{SMA}= 188, N^{1-1} = 202)	14.806 (3.023)	14.703 (2.945)	0.103 (-0.492–0.698)	
**Comorbidities**				
Diabetes (N^{SMA}= 184, N^{1-1} = 189)	15.071 (3.086)	15.437 (3.217)	-0.366 (-1.008–0.276)	0.723
Hypertension (N^{SMA}= 176, N^{1-1} = 188)	15.068 (2.919)	15.082 (3.098)	-0.014 (-0.634–0.606)	
Cardiac Disease (N^{SMA}= 20, N^{1-1} = 17)	15.325 (3.086)	15.559 (3.613)	-0.234 (-2.496–2.028)	
Asthma / Chronic Obstructive Pulmonary Disease (COPD) (N^{SMA}= 11, N^{1-1} = 8)	14.773 (1.850)	16.062 (3.714)	-1.290 (-4.287–1.707)	
Other Chronic Diseases (N^{SMA}= 2, N^{1-1} = 5)	14.000 (3.550)	15.300 (2.294)	-1.300 (-7.935–5.335)	
**Overall** (N^{SMA}= 498, N^{1-1} = 498)	15.352 (3.400)	15.199 (3.280)	0.154 (-0.262–0.569)	

Data are mean (SD). IOP is measured at the start of each appointment and is therefore unaffected by the treatment in the first trial appointment. We use this value as the baseline level for this variable. ‡ In each row, the sample sizes N^{SMA} and N^{1-1} denote the number of observations – across all relevant appointments – at the subgroup level in question (e.g., Female or Male), in SMAs and 1-1s respectively. ¶ This outcome was analysed by means of linear regression. 95% confidence intervals were constructed using the errors clustered at patient level. *** p<0.01, ** p<0.05, *p<0.1 – these p values are associated with the treatment effect within each subgroup.

S27 Table: Baseline intraocular pressure (IOP) level, in prespecified subgroups
