# Supplementary material for: Evidence from the first Shared Medical Appointments (SMAs) randomised controlled trial in India: SMAs increase the satisfaction, knowledge, and medication compliance of patients with glaucoma
Source: PLOS Glob Public Health. 2023 Jul 20;3(7):e0001648. doi: 10.1371/journal.pgph.0001648 (PMC10358908; doi:10.1371/journal.pgph.0001648)
Supplement: S29 Table — (PDF) [file pgph.0001648.s035.pdf]

Prespecified Subgroup‡	SMA	One-On-One	Difference (95% CI) ¶	p value for Interaction
**Gender**				
Female (N^{SMA}= 211, N^{1-1} = 185)	0.650 (0.145)	0.638 (0.161)	0.012 (-0.019–0.042)	0.413
Male (N^{SMA}= 287, N^{1-1} = 313)	0.690 (0.138)	0.693 (0.130)	-0.004 (-0.025–0.018)	
**Location**				
Rural (N^{SMA}= 190, N^{1-1} = 196)	0.700 (0.128)	0.697 (0.144)	0.003 (-0.024–0.030)	0.818
Urban (N^{SMA}= 308, N^{1-1} = 302)	0.656 (0.149)	0.658 (0.143)	-0.001 (-0.024–0.022)	
**Education Level**				
Illiterate (N^{SMA}= 52, N^{1-1} = 64)	0.688 (0.148)	0.668 (0.152)	0.021 (-0.035–0.076)	0.694
Primary School (N^{SMA}= 297, N^{1-1} = 275)	0.678 (0.148)	0.688 (0.145)	-0.010 (-0.034–0.014)	
Secondary School (N^{SMA}= 21, N^{1-1} = 28)	0.652 (0.125)	0.648 (0.131)	0.004 (-0.070–0.078)	
Undergraduate (N^{SMA}= 79, N^{1-1} = 65)	0.662 (0.131)	0.670 (0.112)	-0.008 (-0.049–0.032)	
Postgraduate (N^{SMA}= 49, N^{1-1} = 66)	0.653 (0.126)	0.628 (0.161)	0.025 (-0.028–0.078)	
**Age**				
≤65 (N^{SMA}= 310, N^{1-1} = 296)	0.660 (0.144)	0.648 (0.150)	0.012 (-0.012–0.035)	0.136
>65 (N^{SMA}= 188, N^{1-1} = 202)	0.694 (0.138)	0.709 (0.127)	-0.015 (-0.041–0.012)	
**Comorbidities**				
Diabetes (N^{SMA}= 184, N^{1-1} = 189)	0.673 (0.133)	0.663 (0.144)	0.010 (-0.018–0.039)	0.252
Hypertension (N^{SMA}= 176, N^{1-1} = 188)	0.655 (0.130)	0.672 (0.142)	-0.017 (-0.045–0.011)	
Cardiac Disease (N^{SMA}= 20, N^{1-1} = 17)	0.665 (0.112)	0.665 (0.180)	0.000 (-0.102–0.102)	
Asthma / Chronic Obstructive Pulmonary Disease (COPD) (N^{SMA}= 11, N^{1-1} = 8)	0.723 (0.100)	0.672 (0.206)	0.051 (-0.115–0.216)	
Other Chronic Diseases (N^{SMA}= 2, N^{1-1} = 5)	0.612 (0.104)	0.540 (0.159)	0.072 (-0.178–0.323)	
**Overall** (N^{SMA}= 498, N^{1-1} = 498)	0.673 (0.143)	0.673 (0.144)	0.000 (-0.018–0.018)	

Data are mean (SD). ONH is measured at the start of each appointment and is therefore unaffected by the treatment in the first trial appointment. We use this value as the baseline level for this variable. ‡ In each row, the sample sizes N^{SMA} and N^{1-1} denote the number of observations – across all relevant appointments – at the subgroup level in question (e.g., Female or Male), in SMAs and 1-1s respectively. ¶ This outcome was analysed by means of linear regression. 95% confidence intervals were constructed using the errors clustered at patient level. *** p<0.01, ** p<0.05, *p<0.1 – these p values are associated with the treatment effect within each subgroup.

S29 Table: Baseline optic nerve head cup-to-disk ratio (ONH) level, in prespecified subgroups
