# Supplementary material for: Evidence from the first Shared Medical Appointments (SMAs) randomised controlled trial in India: SMAs increase the satisfaction, knowledge, and medication compliance of patients with glaucoma
Source: PLOS Glob Public Health. 2023 Jul 20;3(7):e0001648. doi: 10.1371/journal.pgph.0001648 (PMC10358908; doi:10.1371/journal.pgph.0001648)
Supplement: S30 Table — (PDF) [file pgph.0001648.s036.pdf]

Prespecified Subgroup‡	SMA	One-On-One	Difference (95% CI) ¶	p value for Interaction
**Gender**				
Female (N^{SMA}= 211, N^{1-1} = 185)	0.649 (0.147)	0.640 (0.165)	0.009 (-0.022–0.041)	0.131
Male (N^{SMA}= 287, N^{1-1} = 313)	0.689 (0.133)	0.694 (0.124)	-0.006 (-0.026–0.015)	
**Location**				
Rural (N^{SMA}= 190, N^{1-1} = 196)	0.699 (0.127)	0.697 (0.143)	0.002 (-0.026–0.030)	0.200
Urban (N^{SMA}= 308, N^{1-1} = 302)	0.656 (0.147)	0.658 (0.137)	-0.001 (-0.024–0.022)	
**Education Level**				
Illiterate (N^{SMA}= 52, N^{1-1} = 64)	0.686 (0.161)	0.670 (0.151)	0.015 (-0.045–0.076)	0.245
Primary School (N^{SMA}= 297, N^{1-1} = 275)	0.681 (0.141)	0.685 (0.142)	-0.005 (-0.028–0.019)	
Secondary School (N^{SMA}= 21, N^{1-1} = 28)	0.642 (0.138)	0.656 (0.130)	-0.015 (-0.096–0.067)	
Undergraduate (N^{SMA}= 79, N^{1-1} = 65)	0.657 (0.134)	0.676 (0.115)	-0.019 (-0.061–0.024)	
Postgraduate (N^{SMA}= 49, N^{1-1} = 66)	0.651 (0.137)	0.630 (0.155)	0.022 (-0.034–0.077)	
**Age**				
≤65 (N^{SMA}= 310, N^{1-1} = 296)	0.660 (0.142)	0.648 (0.147)	0.012 (-0.012–0.035)	0.342
>65 (N^{SMA}= 188, N^{1-1} = 202)	0.695 (0.136)	0.709 (0.127)	-0.014 (-0.040–0.012)	
**Comorbidities**				
Diabetes (N^{SMA}= 184, N^{1-1} = 189)	0.671 (0.131)	0.665 (0.142)	0.005 (-0.023–0.034)	0.000†
Hypertension (N^{SMA}= 176, N^{1-1} = 188)	0.657 (0.130)	0.671 (0.141)	-0.014 (-0.043–0.015)	
Cardiac Disease (N^{SMA}= 20, N^{1-1} = 17)	0.692 (0.161)	0.633 (0.225)	0.059 (-0.094–0.212)	
Asthma / Chronic Obstructive Pulmonary Disease (COPD) (N^{SMA}= 11, N^{1-1} = 8)	0.684 (0.127)	0.726 (0.200)	-0.042 (-0.227–0.143)	
Other Chronic Diseases† (N^{SMA}= 2, N^{1-1} = 5)	0.613 (0.124)	0.540 (0.151)	n/a	
**Overall** (N^{SMA}= 498, N^{1-1} = 498)	0.673 (0.139)	0.673 (0.140)	0.000 (-0.017–0.018)	

Data are mean (SD). ONH is measured at the start of each appointment and is therefore unaffected by the treatment in the first trial appointment. We use this value as the baseline level for this variable. ‡ In each row, the sample sizes N^{SMA} and N^{1-1} denote the number of observations – across all relevant appointments – at the subgroup level in question (e.g., Female or Male), in SMAs and 1-1s respectively. ¶ This outcome was analysed by means of linear regression. 95% confidence intervals were constructed using the errors clustered at patient level. We controlled for the patient's biological sex, age, urbanity, education level, and the presence of comorbidities as well as an indicator variable denoting the identity of the doctor. *** p<0.01, ** p<0.05, *p<0.1 – these p values are associated with the treatment effect within each subgroup. † Due to lack of outcome variation in some of the subgroups, it was only possible to calculate the chi-square p value for the interaction using the subgroups for which we could derive difference and confidence intervals from regression models. Mean (SD) derived from summary statistics when the model could not have been estimated due to lack of variation in one or two arms of one subgroup and resulted in n/a as the difference in means.

S30 Table: Baseline optic nerve head cup-to-disk ratio (ONH) level, in prespecified subgroups with controls
