# Supplementary material for: Evidence from the first Shared Medical Appointments (SMAs) randomised controlled trial in India: SMAs increase the satisfaction, knowledge, and medication compliance of patients with glaucoma
Source: PLOS Glob Public Health. 2023 Jul 20;3(7):e0001648. doi: 10.1371/journal.pgph.0001648 (PMC10358908; doi:10.1371/journal.pgph.0001648)
Supplement: S31 Table — (PDF) [file pgph.0001648.s037.pdf]

Primary Outcomes	SMA	One-On-One	Difference in Means (95% CI)	p value
To what extent do you agree that your doctor has been fair in his or her dealings with you?	4.873 (0.384)	4.766 (0.564)	0.107 (0.043–0.170)	0.001
How much at ease did you feel during the interactions?	4.977 (0.260)	4.904 (0.628)	0.073 (0.010–0.136)	0.023
How caring was the doctor towards you?	4.846 (0.456)	4.735 (0.611)	0.111 (0.400–0.182)	0.002
How caring was the doctor towards the other patients?	4.791 (0.528)	4.688 (0.688)	0.103 (0.023–0.184)	0.012
Did you spend enough time with your doctor?	4.812 (0.499)	4.675 (0.669)	0.137 (0.059–0.214)	0.001

Data are mean (SD). Outcomes were analysed using linear regression. 95% confidence intervals were constructed with errors clustered at the patient level. All questions are evaluated using a 5-point scale – 1 represents much less than expected, 5 represents much more than expected.

S31 Table: Exit Survey
