# Supplementary material for: Evidence from the first Shared Medical Appointments (SMAs) randomised controlled trial in India: SMAs increase the satisfaction, knowledge, and medication compliance of patients with glaucoma
Source: PLOS Glob Public Health. 2023 Jul 20;3(7):e0001648. doi: 10.1371/journal.pgph.0001648 (PMC10358908; doi:10.1371/journal.pgph.0001648)
Supplement: S32 Table — (PDF) [file pgph.0001648.s038.pdf]

Cut†	2 vs 3 4 5			2 3 vs 4 5			2 3 4 vs 5		
**Sample size**	(n = 3 vs n = 3,653)			(n = 18 vs n = 3,638)			(n = 207 vs n = 3,449)		
**Metric**	**coef.**	**std. error**	**p**	**coef.**	**std. error**	**p**	**coef.**	**std. error**	**p**
**Without controls**									
**SMA**	0.682	1.225	0.578	0.948	0.536	0.077	0.586	0.160	0.000
**With controls**									
**SMA**	9.345	3.099	0.003	0.846	0.529	0.110	0.608	0.162	0.000
**Age**	0.832	0.281	0.003	-0.017	0.032	0.597	-0.012	0.009	0.184
**Male**	18.023	1.912	0.000	-0.150	0.522	0.774	-0.066	0.173	0.703
**Second Doctor**	19.749	2.486	0.000	-0.198	0.641	0.757	0.080	0.184	0.664
**Education Level**									
Primary School	-1.780	1.136	0.117	-0.009	0.692	0.990	-0.184	0.257	0.474
Secondary School	-6.729	3.455	0.051	0.012	1.138	0.992	0.111	0.442	0.801
Undergraduate	-22.490	9.336	0.016	1.006	1.344	0.454	-0.197	0.324	0.542
Postgraduate	8.603	3.185	0.007	0.266	1.005	0.791	-0.032	0.355	0.928
**Comorbidities**									
Diabetes	-22.274	2.747	0.000	0.050	0.571	0.930	-0.315	0.158	0.046
Hypertension	-23.953	3.604	0.000	0.262	0.739	0.723	0.268	0.162	0.099
Cardiac Disease	-30.997	6.851	0.000	-0.555	1.084	0.609	-0.403	0.311	0.195
Asthma / Chronic Obstructive	0.000	n/a	n/a	14.612	0.519	0.000	-0.584	0.481	0.225
Other Chronic Diseases	0.000	n/a	n/a	0.000	n/a	n/a	15.632	0.432	0.000

† 1. Very satisfied, 2. Satisfied, 3. Neutral, 4. Dissatisfied, 5. Very dissatisfied

“n/a” represents that the model could not have been estimated due to lack of variation in one or two arms, and resulted in “n/a” as the standard error and p-value.

S32 Table: Satisfaction with the appointment, generalized ordered logit model
