# Supplementary material for: Evidence from the first Shared Medical Appointments (SMAs) randomised controlled trial in India: SMAs increase the satisfaction, knowledge, and medication compliance of patients with glaucoma
Source: PLOS Glob Public Health. 2023 Jul 20;3(7):e0001648. doi: 10.1371/journal.pgph.0001648 (PMC10358908; doi:10.1371/journal.pgph.0001648)
Supplement: S33 Table — (PDF) [file pgph.0001648.s039.pdf]

| Cut† | 1 vs 2 3 4 5 | | | 1 2 vs 3 4 5 | | | 1 2 3 vs 4 5 | | | 1 2 3 4 vs 5 | | |
|---|---|---|---|---|---|---|---|---|---|---|---|---|
| Sample size | (n = 3 vs n = 3,653) | | | (n = 6 vs n = 3,649) | | | (n = 37 vs n = 3,618) | | | (n = 111 vs n = 3,544) | | |
| Metric | coef. | std. error | p | coef. | std. error | p | coef. | std. error | p | coef. | std. error | p |
| **Without controls** | | | | | | | | | | | | |
| **SMA** | 0 | n/a | n/a | 15.163 | 0.410 | 0.000 | 0.857 | 0.361 | 0.018 | 0.830 | 0.215 | 0.000 |
| **With controls** | | | | | | | | | | | | |
| **SMA** | 0.000 | n/a | n/a | 11.632 | 1.049 | 0.000 | 0.900 | 0.381 | 0.018 | 0.800 | 0.216 | 0.000 |
| **Age** | 0.194 | 0.076 | 0.010 | 0.005 | 0.117 | 0.964 | -0.022 | 0.021 | 0.295 | -0.017 | 0.010 | 0.108 |
| **Male** | -28.054 | 1.186 | 0.000 | -0.437 | 2.786 | 0.875 | -0.169 | 0.473 | 0.720 | -0.115 | 0.221 | 0.602 |
| **Second Doctor** | 2.306 | 1.229 | 0.061 | 0.524 | 2.286 | 0.819 | -0.067 | 0.427 | 0.875 | 0.355 | 0.228 | 0.119 |
| **Education Level** | | | | | | | | | | | | |
| Primary School | -5.944 | 4.239 | 0.161 | 15.090 | 2.150 | 0.000 | -1.162 | 0.814 | 0.153 | -0.009 | 0.317 | 0.977 |
| Secondary School | 0.000 | n/a | n/a | 0.000 | n/a | n/a | 10.405 | 0.840 | 0.000 | 1.184 | 0.770 | 0.124 |
| Undergraduate | 0.000 | n/a | n/a | 11.575 | 4.732 | 0.014 | 1.009 | 1.375 | 0.463 | 0.564 | 0.429 | 0.188 |
| Postgraduate | 0.000 | n/a | n/a | 0.000 | n/a | n/a | 11.638 | 0.879 | 0.000 | 0.380 | 0.442 | 0.390 |
| **Comorbidities** | | | | | | | | | | | | |
| Diabetes | 9.618 | 6.490 | 0.138 | 7.197 | 2.157 | 0.001 | -0.119 | 0.425 | 0.780 | -0.403 | 0.206 | 0.050 |
| Hypertension | 9.618 | n/a | n/a | 7.542 | 1.959 | 0.000 | -0.122 | 0.349 | 0.726 | 0.031 | 0.207 | 0.882 |
| Cardiac Disease | 0.000 | n/a | n/a | 9.110 | 1.777 | 0.000 | 0.502 | 1.061 | 0.636 | 0.126 | 0.509 | 0.805 |
| Asthma / Chronic Obstructive | 0.000 | n/a | n/a | 0.000 | n/a | n/a | 10.524 | 0.546 | 0.000 | 0.634 | 1.042 | 0.543 |
| Other Chronic Diseases | 0.000 | n/a | n/a | 0.000 | n/a | n/a | 0.000 | n/a | n/a | 10.528 | 0.478 | 0.000 |

† 1. Fully, 2. Almost fully, 3. Somewhat, 4. Not very well, 5. Not at all
“n/a” represents that the model could not have been estimated due to lack of variation in one or two arms, and resulted in “n/a” as the standard error and p-value.

**S33 Table: Satisfaction with Doubts Addressed, generalized ordered logit model**
