# Supplementary material for: Evidence from the first Shared Medical Appointments (SMAs) randomised controlled trial in India: SMAs increase the satisfaction, knowledge, and medication compliance of patients with glaucoma
Source: PLOS Glob Public Health. 2023 Jul 20;3(7):e0001648. doi: 10.1371/journal.pgph.0001648 (PMC10358908; doi:10.1371/journal.pgph.0001648)
Supplement: S34 Table — (PDF) [file pgph.0001648.s040.pdf]

Cut†	1 vs 2 3 4 5			1 2 vs 3 4 5			1 2 3 vs 4 5			1 2 3 4 vs 5		
Sample size	(n = 2 vs n = 3,653)			(n = 6 vs n = 3,649)			(n = 126 vs n = 3,529)			(n = 399 vs n = 3,256)		
Metric	coef.	std. error	p	coef.	std. error	p	coef.	std. error	p	coef.	std. error	p
**Without controls**												
**SMA**	0	n/a	n/a	15.136	0.410	0.000	0.895	0.207	0.000	0.603	0.113	0.000
**With controls**												
**SMA**	0.000	n/a	n/a	14.314	2.447	0.000	0.894	0.215	0.000	0.613	0.113	0.000
**Age**	0.268	0.047	0.000	0.215	0.191	0.260	-0.013	0.011	0.211	-0.008	0.006	0.188
**Male**	19.793	1.571	0.000	1.902	1.845	0.303	-0.199	0.233	0.394	-0.136	0.126	0.283
**Second Doctor**	-49.127	2.826	0.000	-1.820	2.892	0.529	0.333	0.210	0.114	0.163	0.127	0.200
**Education Level**												
Primary School	-2.746	n/a	n/a	-12.924	2.220	0.000	-1.419	0.527	0.007	-0.692	0.231	0.003
Secondary School	0.000	n/a	n/a	0.455	1.815	0.802	-1.431	0.629	0.023	-0.708	0.347	0.041
Undergraduate	0.000	n/a	n/a	1.065	1.405	0.448	-1.452	0.571	0.011	-0.798	0.272	0.003
Postgraduate	0.000	n/a	n/a	3.502	2.740	0.201	-1.441	0.556	0.010	-0.535	0.274	0.051
**Comorbidities**												
Diabetes	24.816	1.300	0.000	-3.336	2.799	0.233	0.281	0.243	0.247	0.126	0.130	0.333
Hypertension	19.232	1.917	0.000	-0.431	0.817	0.598	-0.416	0.222	0.061	0.002	0.129	0.988
Cardiac Disease	0.000	n/a	n/a	6.392	2.815	0.023	-0.092	0.607	0.879	-0.264	0.271	0.329
Asthma / Chronic Obstructive	0.000	n/a	n/a	11.804	2.159	0.000	0.954	1.124	0.396	-0.019	0.426	0.964
Other Chronic Diseases	0.000	n/a	n/a	0.000	n/a	n/a	11.003	0.803	0.000	1.271	0.868	0.143

† 1. Much more than expected, 2. More than expected, 3. As much as expected, 4. Less than expected, 5. Much less than expected
"n/a" represents that the model could not have been estimated due to lack of variation in one or two arms, and resulted in "n/a" as the standard error and p-value.

S34 Table: Satisfaction with Learning, generalized ordered logit model
