# Supplementary material for: Evidence from the first Shared Medical Appointments (SMAs) randomised controlled trial in India: SMAs increase the satisfaction, knowledge, and medication compliance of patients with glaucoma
Source: PLOS Glob Public Health. 2023 Jul 20;3(7):e0001648. doi: 10.1371/journal.pgph.0001648 (PMC10358908; doi:10.1371/journal.pgph.0001648)
Supplement: S35 Table — (PDF) [file pgph.0001648.s041.pdf]

Cut†	1 vs 2 3 4 5			1 2 vs 3 4 5			1 2 3 vs 4 5			1 2 3 4 vs 5		
Sample size	(n = 1 vs n = 3,654)			(n = 2 vs n = 3,653)			(n = 18 vs n = 3,637)			(n = 64 vs n = 3,591)		
Metric	coef.	std. error	p	coef.	std. error	p	coef.	std. error	p	coef.	std. error	p
**Without controls**												
**SMA**	-4112.34	n/a	n/a	16.868	1.008	0.000	0.867	0.531	0.102	0.215	0.256	0.401
**With controls**												
**SMA**	-83.202	n/a	n/a	20.671	31.990	0.518	0.678	0.850	0.425	0.256	0.249	0.304
**Age**	0.591	0.148	0.000	0.253	1.728	0.884	-0.048	0.026	0.063	-0.045	0.015	0.003
**Male**	-83.202	n/a	n/a	17.125	33.668	0.611	0.940	0.865	0.277	0.558	0.250	0.026
**Second Doctor**	-2.438	n/a	n/a	0.357	2.965	0.904	1.001	0.812	0.218	0.399	0.282	0.157
**Education Level**												
Primary School	-2.438	n/a	n/a	-11.993	58.322	0.837	0.748	1.241	0.547	-0.524	0.397	0.187
Secondary School	0.000	n/a	n/a	0.000	n/a	n/a	18.376	1.463	0.000	0.784	1.070	0.464
Undergraduate	0.000	n/a	n/a	1.487	n/a	n/a	0.812	1.844	0.660	0.331	0.619	0.593
Postgraduate	0.000	n/a	n/a	-6.656	n/a	n/a	1.264	1.782	0.478	0.581	0.818	0.477
**Comorbidities**												
Diabetes	-2.438	n/a	n/a	-34.008	4.579	0.000	-0.006	0.682	0.994	0.149	0.274	0.586
Hypertension	-83.202	n/a	n/a	25.122	42.781	0.557	-0.403	0.692	0.560	-0.006	0.268	0.983
Cardiac Disease	0.000	n/a	n/a	0.000	n/a	n/a	16.111	1.053	0.000	0.931	1.002	0.353
Asthma / Chronic Obstructive	0.000	n/a	n/a	0.000	n/a	n/a	16.954	1.334	0.000	0.122	0.981	0.901
Other Chronic Diseases	0.000	n/a	n/a	0.000	n/a	n/a	0.000	n/a	n/a	16.501	0.467	0.000

† 1. Fully, 2. Almost fully, 3. Somewhat, 4. Not very well, 5. Not at all

"n/a" represents that the model could not have been estimated due to lack of variation in one or two arms, and resulted in "n/a" as the standard error and p-value.

S35 Table: Satisfaction with Understanding Instructions, generalized ordered logit model
