# Supplementary material for: Evidence from the first Shared Medical Appointments (SMAs) randomised controlled trial in India: SMAs increase the satisfaction, knowledge, and medication compliance of patients with glaucoma
Source: PLOS Glob Public Health. 2023 Jul 20;3(7):e0001648. doi: 10.1371/journal.pgph.0001648 (PMC10358908; doi:10.1371/journal.pgph.0001648)
Supplement: S36 Table — (PDF) [file pgph.0001648.s042.pdf]

| Cut† | 2 vs 3 4 5 | | | 2 3 vs 4 5 | | | 2 3 4 vs 5 | | |
|---|---|---|---|---|---|---|---|---|---|
| **Sample size** | (n = 2 vs n = 3,654) | | | (n = 6 vs n = 3,650) | | | (n = 38 vs n = 3,618) | | |
| **Metric** | **coef.** | **std. error** | **p** | **coef.** | **std. error** | **p** | **coef.** | **std. error** | **p** |
| **Without controls** | | | | | | | | | |
| **SMA** | 15.115 | 0.707 | 0.000 | 0.682 | 0.865 | 0.430 | 0.094 | 0.332 | 0.776 |
| **With controls** | | | | | | | | | |
| **SMA** | 50.103 | n/a | n/a | -6.550 | 4.127 | 0.113 | 0.023 | 0.337 | 0.945 |
| **Age** | -2.257 | 0.154 | 0.000 | -0.115 | 0.089 | 0.194 | -0.019 | 0.022 | 0.393 |
| **Male** | 105.894 | n/a | n/a | -29.922 | n/a | n/a | 0.614 | 0.395 | 0.120 |
| **Second Doctor** | 62.804 | n/a | n/a | -31.537 | 4.555 | 0.000 | -0.044 | 0.428 | 0.917 |
| **Education Level** | | | | | | | | | |
| Primary School | -25.559 | n/a | n/a | -46.152 | n/a | n/a | 0.220 | 0.505 | 0.662 |
| Secondary School | 0.000 | n/a | n/a | 3.298 | n/a | n/a | 0.644 | 1.134 | 0.570 |
| Undergraduate | 22.793 | 87.203 | 0.794 | -41.621 | 4.192 | 0.000 | -0.039 | 0.626 | 0.951 |
| Postgraduate | 0.000 | n/a | n/a | 6.132 | n/a | n/a | 0.380 | 0.740 | 0.608 |
| **Comorbidities** | | | | | | | | | |
| Diabetes | 35.252 | 38.866 | 0.364 | 0.767 | 1.502 | 0.610 | -0.527 | 0.347 | 0.129 |
| Hypertension | -30.318 | n/a | n/a | 0.388 | 1.878 | 0.836 | 0.535 | 0.363 | 0.140 |
| Cardiac Disease | 0.000 | n/a | n/a | 0.000 | n/a | n/a | 27.604 | n/a | n/a |
| Asthma / Chronic Obstructive | 33.031 | 44.387 | 0.457 | -36.579 | n/a | n/a | 27.227 | 1.044 | 0.000 |
| Other Chronic Diseases | 0.000 | n/a | n/a | 0.000 | n/a | n/a | 35.904 | n/a | n/a |

† 1. Very likely, 2. Likely, 3. Somewhat likely, 4. Unlikely, 5. Very unlikely

"n/a" represents that the model could not have been estimated due to lack of variation in one or two arms, and resulted in "n/a" as the standard error and p-value.

**S36 Table: Intention to Return, generalized ordered logit model**
