# Supplementary material for: Evidence from the first Shared Medical Appointments (SMAs) randomised controlled trial in India: SMAs increase the satisfaction, knowledge, and medication compliance of patients with glaucoma
Source: PLOS Glob Public Health. 2023 Jul 20;3(7):e0001648. doi: 10.1371/journal.pgph.0001648 (PMC10358908; doi:10.1371/journal.pgph.0001648)
Supplement: S37 Table — (PDF) [file pgph.0001648.s043.pdf]

Cut†	0 vs 1 2 3 4 5			0 1 vs 2 3 4 5			0 1 2 vs 3 4 5			0 1 2 3 vs 4 5			0 1 2 3 4 vs 5		
**Sample size**	(n = 19 vs n = 3,637)			(n = 158 vs n = 3,498)			(n = 889 vs n = 2,767)			(n = 1,990 vs n = 1,666)			(n = 3,010 vs n = 646)		
**Metric**	**coef.**	**std. error**	**p**	**coef.**	**std. error**	**p**	**coef.**	**std. error**	**p**	**coef.**	**std. error**	**p**	**coef.**	**std. error**	**p**
**Without controls**															
**SMA**	0.094	0.505	0.853	0.472	0.180	0.009	0.315	0.091	0.001	0.216	0.080	0.007	0.120	0.100	0.232
**With controls**															
**SMA**	0.184	0.543	0.735	0.515	0.185	0.005	0.321	0.091	0.000	0.228	0.079	0.004	0.122	0.100	0.224
**Age**	-0.026	0.017	0.120	-0.027	0.011	0.011	-0.009	0.005	0.101	-0.011	0.005	0.014	-0.011	0.006	0.069
**Male**	0.096	0.798	0.905	0.274	0.200	0.172	-0.047	0.100	0.637	0.066	0.089	0.461	0.014	0.116	0.902
**Second Doctor**	-0.083	0.548	0.880	0.269	0.182	0.139	0.246	0.093	0.008	0.151	0.084	0.071	0.106	0.108	0.327
**Education Level**															
Primary School	1.626	0.687	0.018	0.583	0.227	0.010	0.623	0.126	0.000	0.636	0.136	0.000	0.303	0.185	0.101
Secondary School	16.615	0.467	0.000	1.304	0.526	0.013	0.957	0.284	0.001	0.977	0.220	0.000	0.433	0.294	0.140
Undergraduate	1.527	1.121	0.173	0.961	0.381	0.012	0.985	0.174	0.000	1.035	0.180	0.000	0.839	0.223	0.000
Postgraduate	2.071	1.215	0.088	1.348	0.407	0.001	1.376	0.203	0.000	1.187	0.181	0.000	0.840	0.225	0.000
**Comorbidities**															
Diabetes	0.959	0.630	0.128	-0.126	0.175	0.471	-0.067	0.099	0.499	-0.082	0.086	0.335	-0.118	0.103	0.252
Hypertension	0.111	0.588	0.851	0.108	0.177	0.540	0.090	0.103	0.379	0.119	0.089	0.183	0.146	0.104	0.162
Cardiac Disease	-1.098	0.893	0.219	0.115	0.517	0.823	-0.370	0.231	0.109	-0.314	0.205	0.126	-0.048	0.220	0.828
Asthma /Chronic Obstructive	12.929	0.645	0.000	-0.894	0.499	0.073	-0.075	0.322	0.816	0.163	0.313	0.603	0.058	0.426	0.892
Other Chronic Diseases	0.000	n/a	n/a	14.888	0.624	0.000	0.860	0.393	0.029	0.207	0.460	0.653	0.214	0.582	0.714

† 0-5 denotes score of each patient from the knowledge survey.
"n/a" represents that the model could not have been estimated due to lack of variation in one or two arms, and resulted in "n/a" as the standard error and p-value.

S37 Table: Knowledge Level, generalized ordered logit model
