# Supplementary material for: Evidence from the first Shared Medical Appointments (SMAs) randomised controlled trial in India: SMAs increase the satisfaction, knowledge, and medication compliance of patients with glaucoma
Source: PLOS Glob Public Health. 2023 Jul 20;3(7):e0001648. doi: 10.1371/journal.pgph.0001648 (PMC10358908; doi:10.1371/journal.pgph.0001648)
Supplement: Attachment — Submitted filename: Response to Reviewers.docx [file pgph.0001648.s044.docx]

From: PLOS Global Public Health <em@editorialmanager.com>

Date: 17 March 2023 at 08:10:04 GMT+5:30

To: Kamalini Ramdas <kramdas@london.edu>

Subject: PLOS Global Public Health Decision: Revision required [PGPH-D-23-00116] - [EMID:dbacb43c2393ec24]

Reply-To: PLOS Global Public Health <globalpubhealth@plos.org>

CC: madhukar.pai@mcgill.ca

PGPH-D-23-00116

Evidence from the First Shared Medical Appointments (SMAs) Trial in India: SMAs Increase the Satisfaction, Knowledge, and Medication Compliance of Patients with Glaucoma

PLOS Global Public Health

Dear Dr. Ramdas,

Thank you for submitting your manuscript to PLOS Global Public Health. After careful consideration, we feel that it has merit but does not fully meet PLOS Global Public Health’s publication criteria as it currently stands. Therefore, we invite you to submit a revised version of the manuscript that addresses the points raised during the review process.

As you can see below, I received two divergent reviews on your manuscript, with Reviewer #2 raising several concerns about the trial methodology and reporting. So, please ensure your revision adequately addresses their concerns.

Please submit your revised manuscript by Apr 30 2023 11:59PM. If you will need more time than this to complete your revisions, please reply to this message or contact the journal office at globalpubhealth@plos.org. When you're ready to submit your revision, log on to https://www.editorialmanager.com/pgph/ and select the 'Submissions Needing Revision' folder to locate your manuscript file.

Please include the following items when submitting your revised manuscript:

A rebuttal letter that responds to each point raised by the editor and reviewer(s). You should upload this letter as a separate file labeled 'Response to Reviewers'.

A marked-up copy of your manuscript that highlights changes made to the original version. You should upload this as a separate file labeled 'Revised Manuscript with Track Changes'.

An unmarked version of your revised paper without tracked changes. You should upload this as a separate file labeled 'Manuscript'.

Guidelines for resubmitting your figure files are available below the reviewer comments at the end of this letter.

We look forward to receiving your revised manuscript.

Kind regards,

Madhukar Pai, MD, PhD

Editor-In-Chief

PLOS Global Public Health

[Note: HTML markup is below. Please do not edit.]

Dear Prof. Pai,

Thank you so much for the opportunity to revise and resubmit our paper, and for the thoughtful and detailed reviews. We have addressed every concern raised and we believe that the manuscript is greatly improved.

We look forward to hearing from you.

Sincerely,

The Authors

Journal Requirements:

1. Please include a complete copy of PLOS’ questionnaire on inclusivity in global research in your revised manuscript. Our policy for research in this area aims to improve transparency in the reporting of research performed outside of researchers’ own country or community. The policy applies to researchers who have travelled to a different country to conduct research, research with Indigenous populations or their lands, and research on cultural artefacts. The questionnaire can also be requested at the journal’s discretion for any other submissions, even if these conditions are not met. Please find more information on the policy and a link to download a blank copy of the questionnaire here: https://journals.plos.org/plosone/s/best-practices-in-research-reporting. Please upload a completed version of your questionnaire as Supporting Information when you resubmit your manuscript.

Thank you for the opportunity to revise and resubmit our manuscript for further consideration for publication in PLOS Global Public Health. We have completed and uploaded the questionnaire in accordance with the policy.

2. Please include an explanation for the retrospective CT registration and confirmation that all related CTs are registered.

This 3.5-year trial was registered with Clinical Trial Registry of India (CTRI) on 22 November, 2016, which is 133 days after the trial commenced on 12 July, 2016. This retrospective CT registration approach was in line with CTRI procedures at the time. In April 2018, CTRI changed its policies to require pre-registration.

3. During your revisions, please confirm whether the wording in the title is correct and update it in the manuscript file and online submission information if needed. Specifically, the article title should specify that the study was a randomised trial, in accordance with the CONSORT checklist. Please also change your manuscript article type to 'Clinical Trial' when you resubmit your manuscript.

Thank you. We have changed our manuscript article type to ‘Clinical Trial’ in our resubmission. We also have changed the title in accordance with the CONSORT checklist. The article is now titled “Evidence from the First Shared Medical Appointments (SMAs) Randomised Controlled Trial in India: SMAs Increase the Satisfaction, Knowledge, and Medication Compliance of Patients with Glaucoma.”

4. Since your data is not available for proprietary reasons, please explain via email why the data is not available. Please also include the contact information for the third party organization that should be contacted should other researchers want to request access to this data and please include the full citation of where the data can be found. We also request that you verify with us via email that any researcher will be able to obtain the data set in the same manner that you have obtained it. If you feel you are unwilling or unable to adhere to this policy, please explain your reasons by return email and your exemption request will be escalated to the editor for approval. Your exemption request will be handled independently and will not hold up the peer review process, but will need to be resolved should your manuscript be accepted for publication. One of the Editorial team will be in touch if they require more information.

As reported in our study protocol in the S2 Appendix, our study participants consented to have their potentially identifying data shared only with the researchers from London Business School and Harvard Business School. Any deviations from the current protocol requires approval of the Institutional Ethics Committee, Aravind Eye Hospital, Thavalakuppam, Pondicherry. The de-identified dataset supporting this research can be requested from the Institutional Ethics Committee, Aravind Eye Hospital, Thavalakuppam, Pondicherry ([pdy.irb-office@pondy.aravind.org](mailto:pdy.irb-office@pondy.aravind.org)).

5. Please provide separate figure files in .tif or .eps format and remove the embedded figures from the manuscript file.

For more information about figure files please see our guidelines:

https://journals.plos.org/globalpublichealth/s/figures

https://journals.plos.org/globalpublichealth/s/figures#loc-file-requirements

Thank you. Figure 1.tif and Figure 2.tif files are provided as separate files. In our resubmission, we also removed the embedded Figure 1 and Figure 2 from the manuscript file.

Reviewers' comments:

Reviewer's Responses to Questions

Comments to the Author

1. Does this manuscript meet PLOS Global Public Health’s publication criteria? Is the manuscript technically sound, and do the data support the conclusions? The manuscript must describe methodologically and ethically rigorous research with conclusions that are appropriately drawn based on the data presented.

Reviewer #1: Yes

Reviewer #2: No

2. Has the statistical analysis been performed appropriately and rigorously?

Reviewer #1: Yes

Reviewer #2: No

3. Have the authors made all data underlying the findings in their manuscript fully available (please refer to the Data Availability Statement at the start of the manuscript PDF file)?

The PLOS Data policy requires authors to make all data underlying the findings described in their manuscript fully available without restriction, with rare exception. The data should be provided as part of the manuscript or its supporting information, or deposited to a public repository. For example, in addition to summary statistics, the data points behind means, medians and variance measures should be available. If there are restrictions on publicly sharing data—e.g. participant privacy or use of data from a third party—those must be specified.

Reviewer #1: Yes

Reviewer #2: No

4. Is the manuscript presented in an intelligible fashion and written in standard English?

PLOS Global Public Health does not copyedit accepted manuscripts, so the language in submitted articles must be clear, correct, and unambiguous. Any typographical or grammatical errors should be corrected at revision, so please note any specific errors here.

Reviewer #1: Yes

Reviewer #2: Yes

5. Review Comments to the Author

Please use the space provided to explain your answers to the questions above. You may also include additional comments for the author, including concerns about dual publication, research ethics, or publication ethics. (Please upload your review as an attachment if it exceeds 20,000 characters)

Reviewer #1: This is a very innovative and interesting study. Overall the paper is well written and well structured. There is adequate context to introduce the topic of SMA and a good description of methodology.

**Comments on Number of patients in a SMA group**

• Line 162 mentioned that participants will be attending with 4 other participants (the mean value), whereas Line 203 mentions 5 and then 2 to 6. It maybe better to clarify in Line 162 that this is the mean value.

Thank you for pointing this out. In Iine 162, by 4 other participants we meant 5 participants in total. Patients experienced SMAs in groups of five during their first appointment – i.e., they attended their appointments with four other patients. However, group size in the subsequent SMAs varied from 2-6 patients (Mean=4.262, SD=0.991), due to scheduling constraints. We have clarified this point in the revised manuscript.

• Can you provide justification for this grouping? Why were they grouped only with 2 to 6 patients? Why not more?

Based on our pre-trial pilots, the need to seat patients comfortably in our consultation room, and coverage by camera for recording the consultation (especially facial recognition), we decided on an optimal group size of 5 patients. During trial appointment 1, we had 5 patients in each group. For the subsequent trial appointments (trial 2, 3, 4) we tried to maintain the same groups with 5 patients in each group. However due to personal reasons, patients could not always make it to their scheduled appointments. Hence, we ended up with having varying group sizes, with 2-6 patients in each group. The revised manuscript now describes this dynamic on page 7.

• Was there a difference in the experience and knowledge between groups that had 2 members vs groups that had 6 members?

As noted above, patients experienced SMAs in groups of five during their first appointment, but group size in subsequent SMAs varied from 2-6 patients due to different scheduling constraints. As we do not have exogenous variation in group size, we are unable to determine whether group size causally affects patient outcomes. However, we conducted a correlational analysis to check the existence of an association between patient outcomes and group size and found that there was no significant correlation between group size and our focal patient outcome variables except for knowledge levels. The correlational analysis indicated that patients in smaller SMA groups exhibited greater knowledge about glaucoma following their appointments. This could mean that smaller groups are more effective than larger ones at transmitting information or that more knowledgeable patients are more likely to reschedule their appointments, such that they wound up in smaller groups. We have left this analysis out of the paper because unobservable factors (such as those related to which types of patients were more likely to reschedule) could be confounds for group size in our study and thus undermine causal inference, but we believe investigating optimal group size is a promising direction for future research.

Comments on Number of investigators

• Can you provide justification for choosing only 2 investigators in Line 193?

We wanted to include investigators who are well experienced in glaucoma care provision and moreover, were well-versed with Tamil, the local language of the region. The glaucoma department at the Aravind Eye Hospital Pondicherry has five consultants with varying levels of glaucoma care experience, and who are from different regions of the country and thus vary in proficiency with the language. We chose the two most-experienced consultants, each of whom has been involved in glaucoma care provision in the region for more than two decades. This approach balanced care experience across providers and enabled us to avoid language barriers during the trial. In the revised manuscript, we allude to this choice on page 6.

• Was there a difference in the knowledge/satisfaction among patients seen by SK vs those seen by RV?

One out of five satisfaction measures and patient knowledge were statistically different for SK and RV. However, assignment of patient groups to SMAs vs. 1-1s was randomized, and therefore doctor characteristics are orthogonal to treatment assignment. In the revised manuscript, we have added a controlled analysis to the S1 Appendix (see Table S3), and we note that the results are substantively similar after controlling for the patient’s biological sex, age, urbanity, the presence of comorbidities, and education level, as well as an indicator variable denoting the identity of the doctor.

Others:

The use of ‘Aravind’ in paragraphs from Line 436 to 448 gives the impression that you are talking about the entire Hospital (Aravind Eye Hospital) rather than ‘the trial’/’our study’

Thank you for raising this. With the revision, we have distinguished carefully between the Aravind Eye Care System, which we refer to by the acronym AECS, and the Aravind Eye Hospital, Pondicherry, where we conducted our trial.

Reviewer #2: In the Introduction section, the authors suggest that in sub-Saharan Africa, half of the healthcare provision is private, and the poorest billion people incur catastrophic out-of-pocket healthcare expenses. The study site, Puducherry, is a relatively affluent site, and the results obtained from very poor sub-Saharan settings cannot be extrapolated to Puducherry. Furthermore, the authors investigated glaucoma, a disease that does not typically result in catastrophic healthcare expenses. Additionally, the authors suggest that even in Sub-Saharan Africa, half of the healthcare is provided by the private sector, and they also mention poorly staffed and crowded government outpatient departments where patients are often turned away without receiving treatment. However, this sweeping generalization is no longer correct.

Thank you for raising this. Our intention was not to extrapolate from sub-Saharan Africa to Pondicherry, but rather to point out that there is a dire need for care delivery innovation in LLMICs, some of which are in sub-Saharan Africa. We agree, however, that this could confuse readers, and we have thus removed the reference to sub-Saharan Africa, referenced data from India – the country of our trial, and refer more generally to LLMICs throughout the paper without citing specific countries.

As we describe in the Introduction section of the revised manuscript, glaucoma is a chronic condition that requires regular follow-up appointments, which many patients cannot afford. As such, as we describe in the Discussion section, we believe that the insights from our trial can be applied beyond Pondicherry to other LLMICs, and for conditions beyond glaucoma, where efficiency and efficacy can be enhanced through shared delivery.

On line 100, the authors mention that in the US, a routine one-on-one appointment for cardiac preventive care can take 30 minutes, whereas a cardiologist may see 8-10 patients in a day. However, this example is not applicable in India, neither in the private nor public sector. It would have been more appropriate if the authors had used large public hospitals, for-profit hospitals, and individual doctors to indicate the number of patients they see and the time they devote to each patient. In India, it is uncommon for an outpatient to receive 30 minutes of attention.

There appears to be some misunderstanding regarding how our example on cardiac preventive care has been interpreted. In the manuscript, we state that “In the US, a routine one-on-one appointment for cardiac preventive care can take 30 minutes, whereas a cardiologist *may see 8-10 patients for this purpose in a 90-minute SMA*”, not “8-10 patients in a day”, as you indicate above. We are unable to use an Indian example because to our knowledge, prior to our trial, SMAs had not been implemented in India. Our objective was to suggest that SMAs, where they have been implemented, have the potential to improve productivity, and we have clarified the language in that section of the exposition to emphasize this point.

Moreover, to further address the spirit of your comment, we have incorporated duration data from our trial in the first paragraph of the discussion section of the revised manuscript.

Several crucial factors that could have affected the study results are not mentioned by the authors on page 5, line 168. These factors include patients' baseline education level, socioeconomic status, and proximity to the hospital. In addition, information is missing regarding the median hospital registration-consultation time and the median time spent per patient by each consultant in the hospital. It is also unclear how many patients attend the glaucoma clinic daily, the number of consultants and residents available in the outpatient department, and the median waiting time for patients in the hospital OPD.

Thank you for raising this. In the revised manuscript, we reference regressions presented in the revised S1 appendix that include controls for patient’s baseline education level, urbanity (whether the patient lives in a rural or urban location), biological sex, age, the presence of comorbidities, and the identity of the doctor (see Tables S3-S22 in the S1 Appendix). We note that all results are substantively similar after incorporating these controls.

Moreover, as we now describe on page 5 in the Methods section, the Aravind Eye Hospital, Pondicherry’s glaucoma clinic, where we conducted our trial, has five glaucoma consultants and four glaucoma fellows who collectively serve approximately 180 patients per day. In March 2023, the Aravind Eye Hospital, Pondicherry’s glaucoma clinic’s median time from registration to exit was 237 minutes, median waiting time was 154 minutes, and median consultation time was 6 minutes.

Page 6, Line 197: Our intervention determined whether groups of patients whose appointments were scheduled proximally were seen separately (in one-on-one appointments) or together (in an SMA). This defeats the purpose of randomization because patients have been preselected.

During the first appointment, we randomized at the group level, rather than at the individual level, because doing so enabled us to use identical scheduling procedures for patients in both arms and ensure consistent experiences beyond the experimental manipulation. However, we maintain assignment to the control or treatment arm of the study at the individual level. Patients who were in a group that was randomly assigned to one arm or the other during the first appointment maintain their randomly-assigned arm throughout the trial. As we describe in the revised manuscript, If the need arose to reschedule a patient due to changes in patient’s or doctor’s availabilities, the coordinator would reschedule the patient, as well as other enrolled patients, to fill in gaps in groups. Managing patients in both arms in groups enabled us to use identical scheduling and reminder procedures to ensure consistent experiences beyond the experimental manipulation. Failure to do so would have created a “performance bias”, i.e., systematic differences in the care that is provided, or in exposure to factors other than the interventions of interest, across the arms (Mansournia 2017).

This sentence is not clear: “but group size in follow-up SMAs varied from 2-6 patients (M=4.262, SD=0.991)” Please spell out “M”- is it mean or median?

Thank you. “M” represented mean throughout the original draft of the paper, but in the revision, we now spell out “mean” in the text.

Statistical analysis

Based on prior research [25] and a power analysis that we conducted using data from the pilot study, we proposed an initial target sample size of 1,000 patients (with 500 in each arm), to ensure a study with 90% power (β=0.1) and the ability to detect differences between sample means with 99% confidence (α=0.01). It is not clear how the authors calculated the sample size based on these numbers. We need to have the estimates of the control and the intervention arms. It is also not clear what the phrase “difference between sample means” refers to. What is the mean in the control arm and the intervention arm? Were the data normally distributed? Why not median?

As we mention in our study protocol in the S2 Appendix, we determined the sample size for our trial by using effect sizes in line with prior research on shared medical appointments (Edelman et al., 2012), and conducting power analyses using data gathered prior to the start of our trial, on knowledge levels and follow-up rates of patients who experienced pilot SMAs and regular one-on-one appointments.

As we now describe in the revised manuscript, using a 10-point knowledge survey administered to 50 patients attending pilot SMAs and 50 patients attending regular one-on-one appointments prior to the start of our trial, the mean knowledge in the SMAs arm was 6.68 (SD = 2.00) and the mean knowledge in the one-on-one arm was 6.18 (SD = 2.07) using the power method (Chow, et al. 2007). The mean difference in knowledge levels was 0.5. Using α =0.01 and β=0.10 we estimated the required sample size to be 493 patients in each arm. Using a similar method, we also examined follow up rate for patients in pilot SMAs and regular one-on-one appointments prior to our trial start and obtained a similar sample size. Based on these analyses we chose an initial target sample size of 1,000 (with 500 patients in each condition). As we had specified that we would do in our protocol, we used trial data collected in the first month of the trial to further calibrate the sample size and decided to continue with 1,000 as our target sample size (500 in each arm).

To the point about the distribution of the data, by the central limit theorem, for sample sizes greater than 5 or 10 per group, the distribution of the sample means will be approximately normally distributed regardless of the original distribution. To test for differences in sample means, the distribution of sample means needs to be normally distributed, not the distribution of the underlying data (Norman 2010). As our sample sizes were large enough, we have used tests that compare means rather than medians, as is customary in the literature (Fagerland 2012).

On page 9, line 263, the authors state that linear regression was used to analyse continuous outcomes. However, it is unclear whether the data were normally distributed, which is important in determining whether means or medians should be used to test differences. Additionally, the authors used logistic regression for discrete outcomes, but they did not explain how they computed the odds ratios and 95% confidence intervals or how they performed the analysis.

As noted above, by the central limit theorem, for sample sizes greater than 5 or 10 per group, the distribution of the sample means will be approximately normally distributed regardless of the original distribution. For standard t-tests or simple linear regressions, normality of the distribution of means is required instead of the normality of dependent or independent variables (Norman 2010). It is also proven that in a large sample, the use of a linear regression technique, even if the dependent variable violates the “normality assumption” rule, remains valid by the law of large numbers and central limit theorem (Wooldridge 2015). Therefore, as noted above, we use tests of differences in means rather than medians, as is customary in the literature (Fagerland 2012). We also note that the appropriateness of this approach extends to models estimated with clustered standard errors (Hansen and Lee 2019).

Moreover, we have updated the Statistical Analysis section of the paper to clarify how we estimated linear, logistic, and generalised ordered logit regressions. As we now note, continuous outcomes were analysed by means of linear regression and binary outcomes were analysed by means of logistic regression. For both regression types, dependent variables were patient outcomes, and the predictor was a dummy variable that denoted whether the patient was a part of the SMA treatment. Predicted means for each arm of the trial are reported using the “margins” command of STATA after running these regressions. Standard errors were clustered at the patient level. Results are presented as mean differences. We note in the paper and show in Table S3 in the S1 Appendix, that all results are substantively similar when we control for the patient’s biological sex, age, urbanity, education level, and the presence of comorbidities as well as an indicator variable denoting the identity of the doctor. For ordinal outcomes, while for ease of exposition we used linear regression, we acknowledge in the revised paper that such models have some limitations, and as we demonstrate in the revised S1 appendix, our results are robust to the use of generalized ordered logit regressions for the treatment effect on our ordinal outcomes (see Tables S32-S37 in the S1 Appendix).

As a robustness test of our results in Table 2 of the manuscript, we also calculated the average primary outcome value for each patient across his/her all appointments, and then ran t-tests rather than running linear or logistic regressions. All results were substantively similar to the ones presented in Table 2 in the manuscript. Below is the summary of the results.

| **Primary Outcomes** | **SMA** | **One-On-One** | **Difference in Means** | **p value** |
| --- | --- | --- | --- | --- |
|  |  |  | **(95% CI) ¶** |  |
|  |  |  |  |  |
| Satisfaction with the Appointment | 4.955 (0.129) | 4.913 (0.195) | 0.042 (0.022,0.063) | <0.0001 |
| Satisfaction with Doubts Addressed | 4.977 (0.096) | 4.935 (0.191) | 0.041 (0.022,0.060) | <0.0001 |
| Satisfaction with Learning | 4.900 (0.193) | 4.803 (0.322) | 0.097 (0.064,0.130) | <0.0001 |
| Satisfaction with Understanding Instructions | 4.981 (0.084) | 4.973 (0.112) | 0.008 (-0.004,0.020) | 0.200 |
| Knowledge Level | 3.387 (0.724) | 3.250 (0.792) | 0.137 (0.042,0.231) | 0.005 |
| Medication Compliance Rate† | 0.967 (0.123) | 0.948 (0.149) | 0.019 (-0.006,0.017) | 0.035 |
| Intention to Return | 4.989 (0.061) | 4.984 (0.113) | 0.006 (0.001,0.036) | 0.337 |
| Probability of Returning within 30 Days† | 0.861 (0.252) | 0.876 (0.224) | -0.015 (-0.045,0.015) | 0.335 |
| Data are mean (SD). ¶ Outcomes were analysed by means of t-tests at the individual level. † We examined data from 2nd, 3th and 4th appointment of the trial for Medication Compliance Rate and Probability of Returning within 30 Days outcomes. For these outcomes, values in the 1^st^ appointment are treatment independent. For other measures, we use data from all four trial appointments. | | | | |
|  |  |  |  |  |
| **Primary outcomes, t-tests** |  |  |  |  |
|  |  |  |  |  |

Five different subgroup analyses were performed based on patients’ sex, age, urban vs. rural residence, education, and comorbidities. The treatment effect within each subgroup was examined by including appropriate treatment-by-subgroup interaction variables in the regression models. Pairwise comparisons of the predictive means were reported after running these regressions to calculate the treatment effect within each subgroup with a 95% confidence interval. A chi-squared test was used to determine whether the effects of SMAs and one-on-one appointments differed across subgroups in each analysis (see Tables S5-S22 in the S1 Appendix). We also note in the revised paper and show in Appendix Tables S5-S22 in the S1 Appendix that all results of the subgroup analyses results are substantively similar when we control for the patient’s biological sex, age, urbanity, education level, the presence of comorbidities as well as an indicator variable denoting the identity of the doctor. All analyses were generated using STATA software version IC 14.2.

The authors also mentioned performing subgroup analyses based on patients' sex, age, urban vs. rural residence, education, and comorbidities, but it is unclear whether they collected data on these variables and how the severity of comorbidities was assessed.

Thank you for seeking this clarification. We have added a new sentence to the Procedures section of the manuscript to clarify that we also captured demographic data for each patient – age, gender, urban/rural residence and education level, as well as medical information, such as glaucoma type and the presence of relevant comorbidities. Summary statistics by trial arm for the demographic and medical information variables, which are provided in Table 1, indicate that we focused on the presence of relevant comorbidities instead of the severity level of those comorbidities.

Table 1 on page 11 should have presented proportions in absolute numbers instead of decimal points.

Thank you for your suggestion. We have updated the proportions in Table 1 accordingly.

The authors reported a difference in patients' overall satisfaction with the appointment, but it is unclear whether this difference is clinically meaningful.

Thank you for raising this. We directly acknowledge this in the discussion section of the revised manuscript, noting that these significant improvements in satisfaction were detected despite high baseline satisfaction levels in the control group across all measured dimensions (M>4.807/5.000 in all cases). To the extent that satisfaction could increase significantly against the backdrop of an already-high baseline, we believe the satisfaction effects we document are more, rather, than less striking.

Furthermore, since Glaucoma is a chronic condition necessitating life-long follow up visits, we conjecture that even incremental increases in appointment satisfaction should help motivate patients to engage in follow-up visits and comply with physician advice. We allude to this possibility in our conclusion, but we also acknowledge that we cannot pinpoint the exact underlying mechanisms that drive observed differences in this study.

Although the authors stated the study's specific aims, objectives, and hypotheses in the CONSORT checklist of information, they did not mention them specifically in the paper's introduction.

Following the guidance in the CONSORT form, we specify the objectives of our study in the Introduction on page 4 of the revised paper as follows:

“Our goal was to assess how SMAs affect two important aspects of patients’ experience (knowledge gained and satisfaction) and two key behavioural outcomes (follow-up rates and medication compliance rates), while also examining two clinical outcomes (change in intraocular pressure (ΔIOP) and change in optic nerve head cup-to-disc ratio (ΔONH)) for patients with primary glaucoma as secondary outcomes. Additionally, we examined physician time per patient and aimed to explore how patients’ perceptions of shared appointments changed as they gained more experience with the format, by analysing verbal and nonverbal engagement measures obtained via video recording of every appointment.”

The study design lacks clarity regarding how the computer-generated sets of random allocations were created, who created them, or whether they were sealed in consecutively numbered opaque envelopes. Blinding during intervention and outcome measurement stages was not adequately specified.

In the randomisation and masking section of the revised paper, we note:

“Enrolled patients were randomly assigned in groups of five to either the treatment arm (SMAs) or the control arm (one-on-one appointments) by means of a random number generator, which ensured allocation concealment [26]. Throughout the trial, medical appointments for patients in the treatment and control arms were scheduled to occur at different times, so as to avoid bias due to patients in one condition observing the experiences of patients in the other condition. As ours was a service delivery intervention, it was not possible to mask patients and study doctors on our research team to group assignment.”

The authors did not provide details on the logistic regression model's development, such as which variables were included in the initial model and how the final model was identified and evaluated.

Thank you for this feedback. We have updated the statistical analysis section of the paper and now note, “Continuous outcomes were analysed using linear regression and binary outcomes were analysed using logistic regression. For both regression types, dependent variables were patient outcomes and the predictor was a dummy variable that denoted whether the patient was a part of the SMA treatment. We present our outcomes as predicted means (SD) for both trial arms. Standard errors were clustered at the patient level. Results are presented as mean differences.”

Following the guidance of the review team, we have also included a controlled set of analyses in the Appendix. As we describe in the exposition, “We note, and show in Table S3-S22 in the S1 Appendix that all results are substantively similar when we control for the patient’s biological sex, age, urbanity, education level, the presence of comorbidities as well as an indicator variable denoting the identity of the doctor.”

For these controlled analyses, we included all the control variables we collected and specified in our protocol. We did not undertake a process through which we iteratively evaluated control variables for inclusion. All included control variables are described in the paper and in the study protocol, which is provided in the S2 appendix.

Overall, the study design, analysis, and presentation of results lack quality, with essential elements such as enrollment, randomization, concealment of the randomization sequence, and blinding not adequately addressed. Additionally, the statistical analysis is inadequate, with essential steps for analyzing data and performing linear or logistic regression not correctly followed. Therefore, based on these limitations, the manuscript should be rejected.

We hope that with the changes and clarifications referenced above that you agree that the manuscript has sufficiently improved to warrant publication. We’re grateful to you, the other reviewer, and the editor for your thoughtful feedback, which has helped us strengthen the manuscript, and we look forward to your decision.

6. PLOS authors have the option to publish the peer review history of their article (what does this mean?). If published, this will include your full peer review and any attached files.

Do you want your identity to be public for this peer review? If you choose “no”, your identity will remain anonymous but your review may still be made public.

For information about this choice, including consent withdrawal, please see our Privacy Policy.

Reviewer #1: No

Reviewer #2: No

**References**

Chow, S. C., Wang, H., & Shao, J. (2007). Sample size calculations in clinical research. CRC press

Edelman, D., McDuffie, J. R., Oddone, E., Gierisch, J. M., Nagi, A., & Williams, J. W. (2012). Shared medical appointments for chronic medical conditions: a systematic review.

Fagerland, M. W. (2012). t-tests, non-parametric tests, and large studies—a paradox of statistical practice?. BMC medical research methodology, 12(1), 1-7.

Hansen, B. E., & Lee, S. (2019). Asymptotic theory for clustered samples. Journal of econometrics, 210(2), 268-290.

Mansournia, M. A., Higgins, J. P., Sterne, J. A., & Hernán, M. A. (2017). Biases in randomized trials: a conversation between trialists and epidemiologists. Epidemiology (Cambridge, Mass.), 28(1), 54.

Norman, G. (2010). Likert scales, levels of measurement and the “laws” of statistics. Advances in health sciences education, 15, 625-632.

Wooldridge, J. M. (2015). *Introductory econometrics: A modern approach*. Cengage learning.
